# Radioactive Labeling of Milk-Derived Exosomes with ^99m^Tc and In Vivo Tracking by SPECT Imaging

**DOI:** 10.3390/nano10061062

**Published:** 2020-05-30

**Authors:** María Isabel González, Pilar Martín-Duque, Manuel Desco, Beatriz Salinas

**Affiliations:** 1 Unidad de Medicina y Cirugía Experimental, Instituto de Investigación Sanitaria Gregorio Marañón, 28007 Madrid, Spain; migonzalez@hggm.es (M.I.G.); bsalinas@hggm.es (B.S.); 2 Unidad de Imagen Avanzada, Centro Nacional de Investigaciones Cardiovasculares (CNIC), 28029 Madrid, Spain; 3 Instituto Aragonés de Ciencias de la Salud (IACS/IIS Aragón), 50009 Zaragoza, Spain; mpmartind@gmail.com; 4 Fundación Agencia Aragonesa para la Investigación y el Desarrollo (ARAID), 50018 Zaragoza, Spain; 5 Centro de Investigación en Red Bioingeniería, Biomateriales y Nanomedicina (CIBER-BBN), 28029 Madrid, Spain; 6 Departamento de Bioingeniería e Ingeniería Aeroespacial, Universidad Carlos III de Madrid, 28911 Madrid, Spain; 7 Centro de Investigación Biomédica en Red de Salud Mental (CIBERSAM), 28029 Madrid, Spain

**Keywords:** exosomes, natural nanoparticles, SPECT imaging, biodistribution, tracking

## Abstract

Over the last decade, exosomes from diverse biological sources have been proposed as new natural platforms in drug delivery. Translation of these nanometric tools to clinical practice requires deep knowledge of their pharmacokinetic properties and biodistribution. The pharmacokinetic properties of exosomes are sometimes evaluated using biochemical and histological techniques that are considerably invasive. As an alternative, we present radiochemical labeling of milk-derived exosomes based on reduced ^99m^Tc (IV) without modifying biological and physicochemical properties. This approach enables longitudinal tracking of natural exosomes by non-invasive single photon emission computed tomography (SPECT) imaging and the evaluation of their pharmacokinetic properties according to the route of administration.

## 1. Introduction

The emerging field of nanomedicine holds great promise in the development of drug delivery systems (DDS) with targeted treatment based on controlled release, especially in the field of oncology [1,2,3]. One of the most favored nanosystems is liposomes, which are spherical vesicles consisting of one or more lipid bilayer membrane(s) encapsulating an aqueous medium. While there is extensive literature on the use of liposomes as DDS in the preclinical field, translation to clinical practice is limited, mainly owing to their inability to evade the host immune system, instability, and toxicity [4]. Natural exosomes and exosome-like systems, on the other hand, are emerging as promising new structures based on the fact that they are similar to liposomes in terms of morphology and size. In addition, they are involved in cell–cell communication, immune response, and tumor progression [5,6,7]. These nanostructures are the smallest cellular vesicles reported to date. They range in size from 50 to 150 nm and are cup-shaped [8]. The lipid bilayer structure present in exosomes enables self-assembly of both hydrophilic and lipophilic substances such as doxorubicin, paclitaxel, antifungal drugs, and analgesics [9,10,11,12,13]. Moreover, depending on their characteristics and origin, their specific tropism can be exploited to steer them towards diseased tissues or organs [14,15]. These biological and physicochemical properties enable exosomes to act as “Trojan horses” for therapeutic agents, thus enhancing their transport to target tissue and increasing their effectiveness. In addition, the biocompatibility and minimal-to-no inherent toxicity of exosomes overcome the limitations observed with most synthetic DDS, thus making them an ideal nanoplatform in the development of new DDS [16].

Use of natural exosomes of non-human origin (such as milk) as nanocarriers has been widely tested in preclinical studies owing to their suitability, scalability, lack of toxicity, and low cost [17,18,19]. However, several limitations still need to be addressed before their translation to clinical practice. These include their largely unexplored natural behavior after exogenous administration and the lack of knowledge about their pharmacokinetic properties as DDS. Such properties are crucial for optimization of dosimetry, where the administration route plays a fundamental role, especially in tissue selectivity and biodistribution [14,20].

Molecular imaging is a well-known, non-invasive technique that enables in vivo assessment of cells, biomolecules, and new therapeutic approaches [21,22] and obviates the removal of tissue samples or organs from their natural environment, thus reducing animal sacrifice and tedious histological assays [23].

Consequently, this technique is extremely useful for the in vivo tracking of exosomes, which is oriented towards the evaluation of pharmacokinetic properties and optimization of dosimetry. Previous studies have assessed in vivo administration routes based on optical imaging after labeling exosomes using fluorescent dyes or genetic engineering techniques [20,24]. Nevertheless, this approach is limited by the inherent background generated by natural biomolecules, such as hemoglobin [25,26,27], or the instability of the probe which leads to incorrect data resulting from the non-specific signal of the free dyes [23]. Furthermore, translation of optical imaging to humans is largely hampered by the limited penetration of light [28]. Nuclear imaging techniques (positron emission tomography (PET) and single photon emission computed tomography (SPECT) may overcome some of these difficulties and seem to be promising approaches owing to their high sensitivity, easy quantification, and wide availability of different radionuclides [23].

This study was the first to evaluate in vivo tracking of natural milk exosomes by nuclear imaging (SPECT) based on labeling with radioactive technetium (^99m^Tc (IV)). The methodology applied enabled us to carry out a complete pharmacokinetic assessment of the radioactive tracer, [^99m^Tc]-Exo, in healthy mice in order to optimize its dosimetry as a DDS. We also studied differences in the natural behavior of the exosomes as a function of the more usual administration routes in the preclinical field (intravenous and intraperitoneal injection and intranasal instillation).

## 2. Materials and Methods 

### 2.1. Isolation of Milk-Derived Exosomes

Exosomes were isolated from commercial fresh pasteurized semi-skimmed goat milk (El Cantero de Letur, Albacete, Spain) by successive centrifugations at 4 °C in 30 mL polycarbonate centrifuge tubes, using a Ja 30,50 Ti rotor (Beckman Coulter Instruments, Brea, CA, USA). Briefly, milk was centrifuged for 10 min at 5,000× *g*, 35 min at 13,000× *g*, and 15 min at 35,000× *g* in order to remove contaminants such as fat globules (MFGs) and cell debris. Additionally, microbial rennet was used to precipitate milk casein. The supernatant was ultracentrifuged at 100,000× *g* and 4 °C for 70 min to precipitate the exosomes (50–150 nm). The resultant pellet was washed three times with phosphate-buffered saline (PBS 1X) and then purified by size exclusion chromatography (SEC) using PD-10 columns (GE Healthcare Bio-Sciences AB, Chicago, IL, USA). Pure exosomes were ultracentrifuged again at 100,000× *g* for 90 min, and the exosome pellet was dispersed in 100 µL of PBS 1X. The protein content of the final sample was quantified using the Coomassie–Bradford assay and stored in aliquots at −20 °C until use.

### 2.2. Protein Content

Protein content was quantified using the Bradford–Coomassie colorimetric assay. A standard calibration curve was obtained using known concentrations of bovine serum albumin (from 2000 µg/mL to 8 µg/mL) and fitting a third-order polynomial equation (*R*^2^ > 0.99), employing MATLAB software (MathWorks Inc., Natick, MA, USA). This standard calibration curve was used to determine the protein content of the exosome samples with 10 µL of exosome solution. Absorbance at 595 nm was measured using a Synergy™ HT Multi-Mode Microplate Reader (Biotek Instruments Inc., Winooski, VT, USA).

### 2.3. Radiolabeling of Goat Milk Exosomes with Reduced Technetium (^99m^Tc (IV))

Unless otherwise stated, all reagents were purchased from Sigma–Aldrich (St. Louis, MO, USA) and used without further purification. Commercial sodium pertechnetate, [^99m^Tc] NaTcO_4_, was obtained from a TEKCIS™ Technetium ^99m^Tc Generator (Curium Pharma, Madrid, Spain). For radiolabeling of exosomes, [^99m^Tc] NaTcO_4_ (30 µL, 5 mCi) was initially reduced in the presence of 20 µL SnCl_2_ in HAc (10%). To determine the optimal reaction conditions, several concentrations of SnCl_2_ were tested (0.0004 M, 0.002 M, 0.004 M, 0.006 M, 0.008 M, and 0.01 M). The reduction reaction was carried out for 5 min at 37 °C under an N_2_ atmosphere and further neutralized with NaOH (10 µL, 2.8 N).

Exosomes isolated at different concentrations (10 µg, 30 µg, and 75 µg; 50 µL) were finally radiolabeled with a solution of ^99m^Tc (IV) in a thermomixer at 37 °C with shaking for 30 min. The resulting products were purified using exosome spin columns (Invitrogen, Carlsbad, CA, USA). The radiochemical yield of the reaction was evaluated by iTLC analysis (silica-gel; mobile phase = 90:10 MeOH:H_2_O; Merck, Germany). The radiopurity of the nanotracer was measured using high-performance liquid chromatography (HPLC, Agilent 1200 series; 0.2 mL/min; 254 nm; mobile phase = PBS 1X; Agilent Technologies, Santa Clara, CA, USA).

Reaction yields were calculated by measuring the activity of purified radiolabeled exosomes using a Genesys LTI gamma counter (Laboratory Technologies Inc., Elburn, IL, USA).

### 2.4. Radiolabeling of Milk Exosomes with Commercial Pertechnetate (^99m^Tc (VII))

The possibility of active labeling based on the iodide symporter (NIS) was assessed by radiolabeling milk exosomes with commercial pertechnetate (^99m^Tc (VII)). Thirty microliters of sodium pertechnetate (5 mCi; Curium Pharma, Madrid, Spain) was mixed with 20 µL of HAc (10%) for 5 min at 37 °C and shaken under an N_2_ atmosphere. Then, the solution was neutralized with NaOH (10 µL of 2.8 N), and over 30 µg of goat milk exosomes (50 µL) was added. The reaction was maintained at 37 °C with shaking for 30 min. The final product was purified by exosome spin columns (Invitrogen, Carlsbad, CA, USA). The radiochemical yield of the reaction was evaluated by iTLC analysis (silica-gel; mobile phase = 90:10 MeOH:H_2_O; Merck, Germany).

The reaction yield was calculated by measuring the activity of the purified product using a Genesys LTI gamma counter (Laboratory Technologies Inc., Elburn, IL, USA).

### 2.5. High-Performance Liquid Chromatography (HPLC)

High-performance liquid chromatography (HPLC) was performed using an Agilent 1200 series HPLC system. Radio-HPLC was performed using an identical Agilent system which was also equipped with a Gina Scan-RAM Radio-TLC/HPLC detector (Microbeam, Spain). Analytic runs were performed on a Yarra SEC-3000 column (300 × 7.8 mm; Phenomenex Inc., Torrance, CA, USA). The solvent system was PBS 1X for the quality control of the radiotracers with a gradient of 100% between 0 and 80 min and a flow of 0.2 mL/min. Data were processed using Gina Star (Microbeam S.A., Madrid, Spain).

### 2.6. Transmission Electron Microscopy (TEM)

Nanovesicles suspensions of both non-radioactive and radiolabeled exosomes were deposited onto a formvar carbon-coated copper grid and stained with uranyl acetate at room temperature. A JEOL JEM-1010 transmission electron microscope (JEOL USA Inc., Peabody, MA, USA) from ICTS Centro Nacional de Microscopía Electrónica (Universidad Complutense de Madrid, Spain) was employed to observe the samples.

### 2.7. Dynamic Light Scattering (DLS) Analysis

Aliquots of the corresponding exosomes were passed through a 0.44 µm filter, and the size distribution was analyzed using a Zetasizer Nano device (Malvern Panalytical, Malvern, UK). The parameters selected were protein as the sample material, 25 °C, 120 seconds of equilibration time, and water as dispersant. Disposable cuvettes DTS0012 (Brand, Germany) were used as measurement cells.

### 2.8. In Vitro Stability Studies

The in vitro stability of radiolabeled exosomes was assessed by incubating 150 µCi of [^99m^Tc]-Exo in PBS 1X from 1 h to 48 h at 37 °C. Aliquots of 3 µl of solution were analyzed at each time point by iTLC on a silica-gel plate (Merck, Germany) and in a mobile phase of 90:10 MeOH:H_2_O.

A Genesys gamma counter (Laboratory Technologies Inc., Elburn, IL, USA) was used to measure the activity of the iTLC regions.

### 2.9. Ethics Statement

The animal experiments complied with the ARRIVE guidelines and were in accordance with the EU Directive (2010/63/EU) for animal experiments. The Hospital General Universitario Gregorio Marañón Animal Care and Use Committee approved all the procedures (PROEX 097/16).

### 2.10. Blood Half-Life

Blood half-life was determined by measuring activity in serial blood samples. Specifically, [^99m^Tc]-Exo tracer was administered to healthy female Balb/C mice (14–18 weeks old, 20–25 g in weight, *n* = 3) via intranasal instillation (30 µL in PBS, 110–190 µCi, 9 ± 2 µg), intraperitoneal injection (300 µL, 350–490 µCi, 24 ± 5 µg), or intravenous tail injection (300 µL, 100–170 µCi, 19 ± 8 µg). Except for the intranasal route, tracer was administered under 2% sevoflurane anesthesia (O_2_ 100%; 200–400 cc/min; Zoetis, Belgium). Blood samples were extracted from the saphenous vein in awake mice at several time points post-injection. This blood was weighed, and radioactivity was measured on a Wallac Wizard 1480-011 Automatic Gamma Counter (Perkin Elmer, Waltham, MA, USA). Measurements in counts per minute were normalized to the mean% ID/g of tissue. Data were modeled as a two-compartmental kinetic model and analyzed by two-phase decay nonlinear regression with Prism 6.0c (GraphPad Software, La Jolla, CA, USA).

### 2.11. In Vivo SPECT/CT Imaging

The SPECT (single photon emission computed tomography) and CT (computed tomography) scans were acquired from healthy female Balb/C mice (14–18 weeks old, 20–25 g in weight, n = 6 per administration route) after administration of the [^99m^Tc]-Exo tracer by intranasal instillation (30 µL in PBS, 140–170 µCi, 12 ± 4 µg), intraperitoneal injection (300 µL, 190–340 µCi, 19 ± 5 µg), or intravenous tail injection (300 µL, 310–350 µCi, 18 ± 9 µg). Images were acquired with the animals under 2% sevoflurane anesthesia (O_2_ 100%; 200–400 cc/min; Zoetis, Belgium).

Longitudinal in vivo tracking of the radiolabeled exosomes was carried out using SPECT (MiLabs USPECT II, Utrecht, The Netherlands) and CT (PET/CT SuperArgus, SEDECAL Molecular Imaging, Madrid, Spain) small animal imaging scanners. The animals were placed in the prone position and the field of view was adjusted to the area of interest. Once the radiotracer was administered, 18 frames of 5 min per animal were acquired and another 60 min scan was performed 24 h post-injection. A multi-pinhole 1.0 collimator was used for acquisition of the SPECT images, and, for the radionuclide ^99m^Tc, an energy window ranging from 126 to 154 KeV was chosen. The number of counts acquired ranged between 1.5 M and 350 K in the worst cases (24 h studies). The OS-EM reconstruction was performed with a 0.75 mm^3^ voxel size, 16 subsets, and 2 iterations using proprietary software (MiLabs, Utrecht, The Netherlands). For correcting disperse events, 2 windows of 20% were applied to the left and right of the radioactive technetium peak, and reconstruction was completed under a Gaussian blurring postfilter with a FWHM of between 0.85 and 1 mm. For acquisition of anatomical images, the CT parameters selected were 40 KeV, 340 µA, 360 projections, and 2 × 2 binning. In addition, images (0.12 mm^3^) were reconstructed using the software from the PET/CT system (SEDECAL Molecular Imaging, Madrid, Spain). No scatter or attenuation corrections were applied.

Both SPECT and CT images were co-registered with the MMWKS software package (SEDECAL Molecular Imaging, Madrid, Spain) [29] using 3 fiducial markers previously charged with contrast and radioactive agents.

### 2.12. Ex Vivo Biodistribution Studies

Biodistribution experiments were conducted on healthy female Balb/C mice (14–18 weeks old, 20–25 g in weight, *n* = 3 per administration route). The radioactive exosome [^99m^Tc]-Exo preparation (250–300 µCi in 300 µL of a PBS 1X solution for the injections and 145–160 µCi in 30 µl of PBS for the intranasal instillation) was administered via the lateral tail vein, intraperitoneally, or intranasally, and the nanotracer was allowed to circulate for 24 h in the 3 groups. After this time, mice were sacrificed and their organs harvested (i.e., brain, trachea/thyroid, lungs, heart, stomach, liver, spleen, kidneys, intestines, muscle, and skin, as well as feces). The activity (exosome content) in the tissue of interest was measured on a Wallac Wizard 1480-011 Automatic Gamma Counter (Perkin Elmer, Waltham, MA, USA) and expressed as mean% ID/g of tissue.

### 2.13. Autoradiography

Healthy female Balb/C mice (14–18 weeks old, 20–25 g in weight, *n* = 3 per administration route) received 16 ± 2 µg/100–210 µCi in 300 µL (PBS 1X) of [^99m^Tc]-Exo via intraperitoneal or intravenous injection, and 10 ± 4 µg/160–200 µCi in 30 µL of PBS via intranasal instillation. The mice were sacrificed after 24 h of circulation (90 min in the case of intranasal administration). Liver and brain tissue were excised, and series of 100–200 µm sections were sliced and mounted on plastic slides. Digital autoradiography was performed to determine radiotracer distribution by placing samples on the plate (BAS-MP 2025, Fujifilm, Tokyo, Japan) for 18 h at room temperature. Phosphor imaging plates were read at a pixel resolution of 200 µ with a Bio-Imaging Analyzer BAS-500 plate reader (Fujifilm, Tokyo, Japan). Tissue intensity was quantified using Fiji ImageJ Software (U.S. National Institutes of Health, Bethesda, MD, USA).

### 2.14. Data Analysis

Data were processed using Prism 6.0c (GraphPad Software, La Jolla, CA, USA).

## 3. Results

### 3.1. Protein Content

The most common method for estimating the number of exosomes per aliquot is currently quantification of protein content in the exosomal dispersion [30]. The Bradford–Coomassie colorimetric assay yielded 197 ± 64 µg of exosomes per 60 mL of goat milk.

### 3.2. Synthesis of Radioactive Exosomes ([^99m^Tc]-Exo) and Optimization

**Figure 1:**
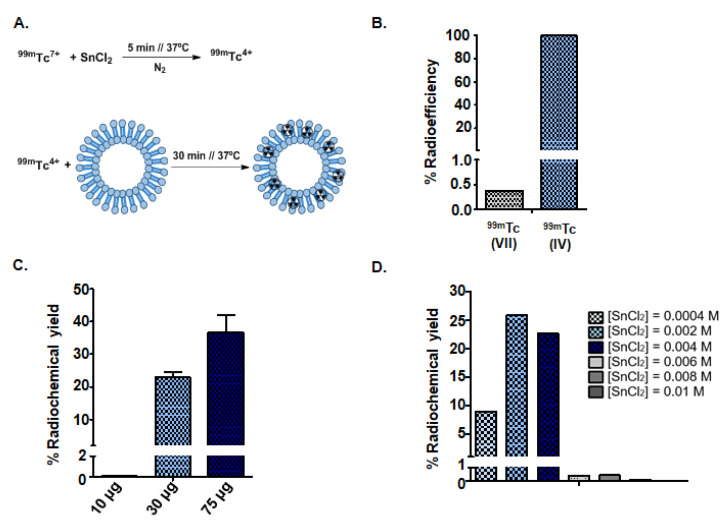
Chemical optimization of goat milk exosome radiolabeling conditions: (**A**) Schematic representation of the reaction. (**B**) Radioefficiency of exosomes labeled with ^99m^Tc (VII) vs. ^99m^Tc (IV). (**C**) Effect of the exosome protein concentration in the radiochemical yield. (**D**) Determination of optimum concentration of SnCl_2_ as a reducing agent.

Reducing conditions were optimized using a standard range of 4 × 10^−4^ M–1 × 10^−2^ M of SnCl_2_ as recommended in the literature [31]. We also tested several concentrations to achieve robust parameters for radiolabeling, reaching optimal conditions that generated the lowest degradation and aggregation effects in the sample (Figure 1A). Lower SnCl_2_ concentrations were not sufficient to reduce the radioisotope and achieved low radiochemical yields (8.95%). Conversely, higher concentrations led to degradation of the exosomes which was confirmed by the TEM image and resulted in a radiolabeling yield of 0.10%. The SnCl_2_ concentrations around 0.002 mM showed the highest radiochemical yield values (25.85%) and the TEM image demonstrated that the initial exosome morphology remained unaltered. Moreover, we realized that the reaction yields also depended on the number of exosomes. The highest yields of radiolabeling (37.00 ± 9.00%) were obtained for 75 µg of exosomes (Figure 1C,D).

Finally, we evaluated the effect of oxidation state using commercial ^99m^Tc (VII) (pertechnetate) and reduced ^99m^Tc (IV). Significant differences were observed (Figure 1B): the radio efficiency achieved using the reduced form of technetium was 99.5%, as measured using iTLC analysis. However, exosomes did not incorporate ^99m^Tc (VII) (radio efficiency of 0.4%).

### 3.3. Physicochemical Characterization

#### 3.3.1. Size and Morphology

**Figure 2:**
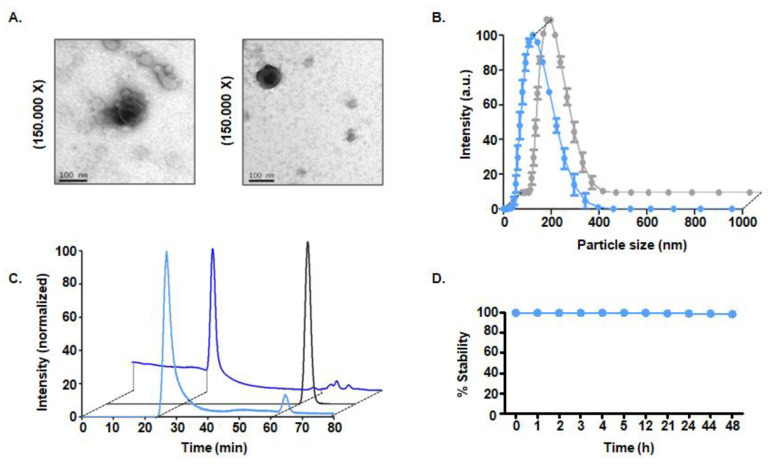
Physicochemical characterization of [^99m^Tc]-Exo. (**A**) Transmission electron microscopy images of non-labeled goat milk exosomes (left) and radiolabeled exosomes (right). (**B**) Hydrodynamic size measured using dynamic light scattering (non-labeled exosomes in blue and radiolabeled exosomes in grey). (**C**) Radioactive HPLC chromatogram of [^99m^Tc]-Exo (light blue), free ^99m^Tc (grey), and UV HPLC chromatogram of non-labeled goat milk exosomes (dark blue). (**D**) Longitudinal in vitro stability study of [^99m^Tc]-Exo.

Assessment of exosome morphology using TEM showed a monodisperse population of the goat milk nanovesicles, with a common morphology consisting of a “cup-shaped” structure that was similar in both radiolabeled and control samples. These nanoparticles presented a core size of approximately 100 nm before and after labeling. Consistently, DLS analysis confirmed a hydrodynamic size of 122.00 ± 1.00 nm for non-labeled exosomes and 114.00 ± 8.00 nm for radiolabeled exosomes (Figure 2A,B).

#### 3.3.2. Radiopurity of [^99m^Tc]-Exo Tracer

The purity of the [^99m^Tc]-Exo was determined by HPLC (Figure 2C), which confirmed a radiopurity > 95%. The main peak in the radioactive chromatogram of [^99m^Tc]-Exo was observed at 24.9 min, matching the retention time of pure non-labeled exosomes in the UV chromatogram (254 nm) (Figure 2C).

#### 3.3.3. In Vitro Stability Studies

The high stability of the new radiotracer—95% even after 48 h—was confirmed in vitro (Figure 2D).

### 3.4. Pharmacokinetic Study and In Vivo Tracking 

#### 3.4.1. Intravenous Administration

**Figure 3:**
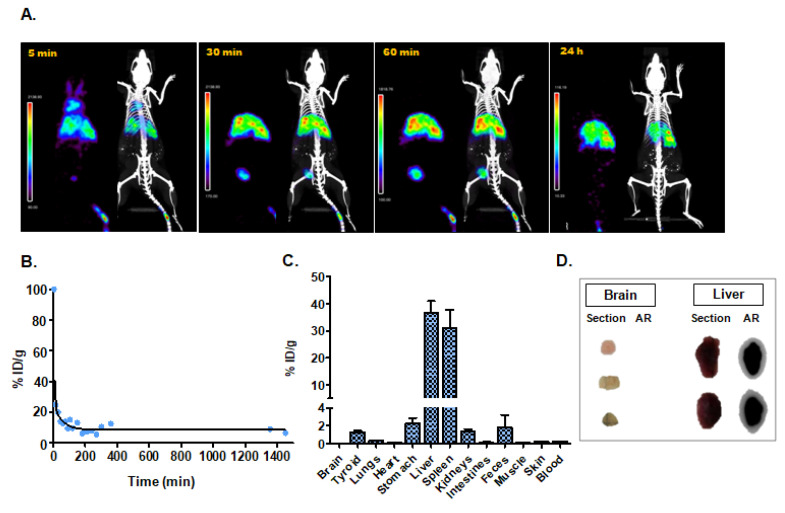
Assessment of intravenous administration. (**A**) In vivo single photon emission computed tomography (SPECT) and computed tomography (CT) imaging of [^99m^Tc]-Exo 5 min, 30 min, 60 min, and 24 h post-injection. (**B**) In vivo blood half-life. (**C**) Ex vivo biodistribution study 24 h after injection. (**D**) Histological evaluation by digital autoradiography (AR) and digital picture (section) of brain and liver.

Small animal SPECT/CT studies were carried out after administering radioactively labeled [^99m^Tc]-Exo to healthy mice. Longitudinal tracking revealed fast blood clearance of these nanotracers. SPECT images at 5 min post-injection still showed circulation of the radioexosomes in the aorta and lungs, and images at 10 min indicated the beginning of accumulation in the liver. The high activity accumulation observed in bladder at 30 min and 60 min confirms a fast-urinary excretion. Spleen and liver uptake were confirmed after 1 h, and no significant changes in the exosome distribution were observed in further acquisitions at 3 h, 5 h, and 24 h (Figure 3A). The in vivo blood half-life study matched the results of SPECT imaging, showing quick blood clearance (Figure 3B) (Table 1). Ex vivo analysis of the biodistribution of [^99m^Tc]-Exo 24 h after its administration also confirmed the data observed in vivo by SPECT (Figure 3C) (Table 1). These data are consistent with ex vivo autoradiography findings (Figure 3D). Histological sections of liver showed a clear delineation of hepatic tissue with [^99m^Tc]-Exo which was not the case for brain tissue with a radioexosome signal in liver tissue that was 10× higher than in brain tissue (Figure 3D).

#### 3.4.2. Intraperitoneal Administration

**Figure 4:**
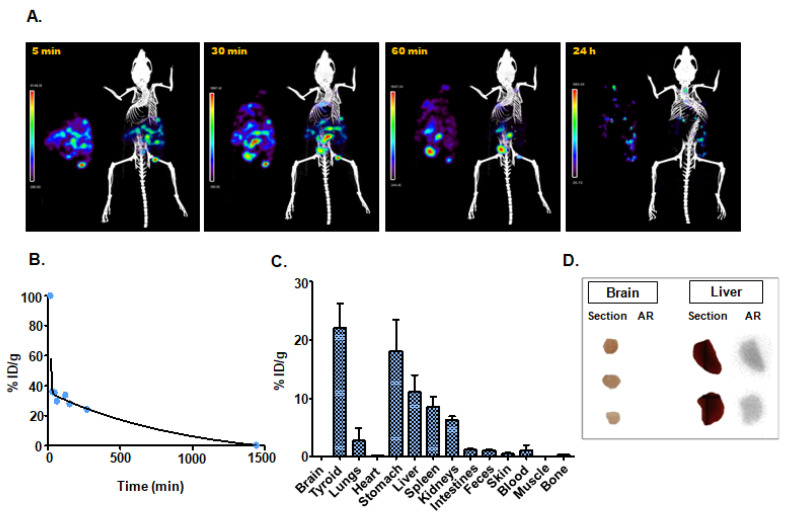
Intraperitoneal administration: (**A**) In vivo SPECT/CT imaging of [^99m^Tc]-Exo 5 min, 30 min, 60 min, and 24 h post-injection. (**B**) In vivo blood half-life. (**C**) Ex vivo biodistribution study 24 h after injection. (**D**) Histological evaluation by digital autoradiography (AR) and digital picture (section) of brain and liver.

The SPECT imaging acquired at 5 min post-injection of [^99m^Tc]-Exo in healthy mice showed the main localization of the nanotracer in the abdominal cavity. Differences in the biodistribution of the radioactive exosomes were observed over time, with high accumulation in the spleen at 30 min post-injection and in the thyroid region at 3 h post-injection (Figure 4A). In vivo measurement of the blood circulation time of the radiotracer showed values 4 fold higher than those observed with intravenous administration (Figure 4B) (Table 1). Ex vivo biodistribution studies confirmed the accumulation of exosomes observed with SPECT imaging (Figure 4C) (Table 1), and autoradiography showed [^99m^Tc]-Exo uptake in liver histological sections with no accumulation in brain. The activity of [^99m^Tc]-Exo in liver was 2 fold higher than in brain tissue (Figure 4D).

#### 3.4.3. Intranasal Instillation

**Figure 5:**
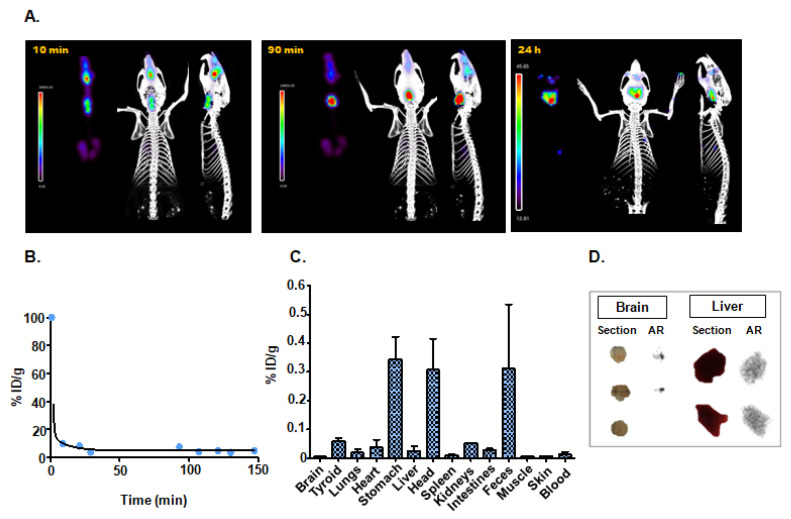
Intranasal administration: (**A**) in vivo SPECT/CT imaging of [^99m^Tc]-Exo 10 min, 90 min, and 24 h post-injection; (**B**) in vivo blood half-life; (**C**) ex vivo biodistribution study 24 h after injection; (**D**) histological evaluation by digital autoradiography (AR) and digital picture (section) of brain and liver.

The SPECT/CT and ex vivo studies were carried out 10 min, 90 min, and 24 h after administration of exosomes. The in vivo SPECT/CT image at 10 min post-injection showed the presence of activity in the nasal cavity and trachea, as well as in the lungs, with no significant changes at 24 h (Figure 5A). Measurements in blood samples confirmed a very short blood circulation half-life (Figure 5B) (Table 1), and ex vivo biodistribution supported the data observed in nuclear images, suggesting the elimination of the nanotracer through the digestive system (Figure 5C) (Table 1).

Although SPECT did not reveal brain uptake in vivo at any time point during the study, the higher sensitivity achieved with autoradiography enabled us to detect the presence of radioactive exosomes in the brain, specifically in the cerebellum. In this case, the signal intensity of [^99m^Tc]-Exo in brain tissue was two-fold higher than in liver tissue (Figure 5D).

## 4. Discussion

Over the last decade, extracellular vesicles, particularly exosomes, have emerged as new nanometric DDSs [10,11,12,15]. These are remarkably interesting because of their natural origin, which gives them an inherent cell/tissue targeting mechanism, as well as their lack of toxicity and suitable biocompatibility for possible translation to clinical practice. Although this promising application has been extensively verified, with some exosome-based therapy systems already being assessed in clinical trials [32,33], knowledge of the pharmacokinetic properties of the nanosystems remains incomplete. In this context, molecular imaging is an ideal tool owing to its non-invasive character as well as its high sensitivity and specificity.

To our knowledge, ours is the first study to assess direct labeling of natural exosomes with the radioisotope ^99m^Tc (IV) in an ionic salt form (^99m^TcCl_4_) using a novel and straightforward methodology. The radiolabeling procedure was optimized using goat milk exosomes because of their scalability and robustness. Previous studies [34,35] achieved radioactive labeling of exosome-like nanovesicles with radioactive technetium using the complex ^99m^Tc-HMPAO or ^99m^Tc-tricarbonyl. These approaches required commercial kits with more expensive and complex radioactive precursors that imply longer reaction times and more complex chemistry for the incorporation of the radionuclide. In contrast, our approach obviates the need for chelators and reduces the impact of the chemical reaction on the exosome structure while providing a suitable reaction yield, high purity, and long-term stability. In addition, the use of milk exosomes enabled us to obtain higher amounts of exosomes than with exosome-like vesicles. Furthermore, our reaction was carried out under mild conditions (pH 7, 37 °C) so as to avoid non-biological conditions that could alter the properties of the exosomes.

The complete the characterization of the radioactive exosomes obtained proved that our radiochemical approach did not change the physicochemical properties of the original exosomes such as size and morphology; this is essential for their further use as DDSs. With respect to the mechanism for incorporating the radioisotope into the exosomes, the absence of radiolabeling with ^99m^Tc (VII) pertechnetate enabled us to rule out biological active transport based on a sodium-dependent iodide transporter, NIS [36]. In fact, the high reaction yields achieved using the ionic form of technetium, ^99m^Tc (IV), point to passive surface labeling, as reported in previous studies on synthetic liposomes [37,38,39], where the radionuclide remained in the hydrophobic by chelation with the phosphonate groups of the membrane [40,41]. Based on these data we interpret that the incorporation and retention of the ^99m^Tc (IV) into the exosomal membrane is based on the coordination with the phosphonate groups that form the lipids present in the membrane. The high stability of this passive incorporation was confirmed by in vitro studies (in PBS 1X) of the degradation of radioactive exosomes, even after days. Due to the comparable molecular weight of plasma proteins and exosomes we did not attempt similar studies employing mouse plasma. This approach significantly outperformed previous exosome-labeling studies [20], where the instability of the modified exosome led to the release of the probe signaling component, in this case a dye, and produced high levels of unspecific signal in their in vivo application.

Although previous works have widely employed nuclear imaging for the evaluation of in vivo distribution of extracellular vesicles [42,43], this is the first time that this approach is employed in the evaluation of the effect of the administration route in the pharmacokinetic of the exosomes by means of SPECT/CT. The results obtained comparing three different administration routes (intravenous, intraperitoneal, and intranasal) showed noticeable differences, in agreement with previous ex vivo studies using similar exosomes with optical labeling [10].

Intravenous administration confirmed fast blood clearance (<4 min) and predominantly hepatic uptake, probably due to active uptake by hepatocytes or Kupffer cells. This natural uptake of exosomes in liver and spleen observed in vivo by SPECT/CT imaging, as well as in ex vivo studies with autoradiography and a gamma counter, has been thoroughly reported in other ex vivo studies using exosomes of different origins [10,20,44], as well as with synthetic nanoparticles that are similar to liposomes in morphology and size [45]

In contrast, intraperitoneal administration led to circulation times three- to four-fold longer than those observed with the intravenous route, probably because of their slower incorporation into the bloodstream. In this case, our in vivo imaging and ex vivo biodistribution studies confirmed the existence of abdominal activity for at least 1 h, with lower liver uptake. This route also presented higher uptakes values of [^99m^Tc]-Exo in lung tissue than the intravenous route, probably owing to aggregation of nanovesicles, as occurs with other synthetic nanoparticles [46]. A significant difference between these routes is the presence of thyroid uptake, which suggests the presence of free Tc, similar to previous studies that showed the natural uptake of other ionic technetium complexes (pertechnetate) in thyroid tissue due to NIS [47,48]. As the stability of [^99m^Tc]-Exo was verified both in vitro and in vivo, the results point to possible exosome degradation related to their peritoneal absorption, which should be carefully considered before using this route for drug delivery, as the cargo could be released prematurely, i.e., before reaching the target.

Finally, intranasal instillation showed probe accumulation in the mouse head (nose), probably due to the retention of the radioactive [^99m^Tc]-Exo during the breeding of the animal. In vivo SPECT imaging failed to detect radioactive exosomes in brain tissue. Nevertheless, the higher sensitivity of autoradiography enabled us to confirm the existence of uptake in brain tissue, similar to results previously reported with optical imaging [49].

Our study was subject to a series of limitations stemming from the use of goat milk exosomes. From a chemical standpoint, the synthesis conditions were optimized for the radiolabeling of these exosomes, although the methodology may need to be re-adjusted for other cell-derived exosomes in order to maintain or improve the radiochemical yield, purity, and stability achieved with milk-derived exosomes. From a biological standpoint, pharmacokinetic results can only be applicable to milk-derived exosomes, since it is well known that the exosomal origin induces molecular differences in membrane composition, which modulates their specific tissue tropism [38].

The complete assessment presented in our study confirms the success of our novel radioactive labeling method, which does not alter the original physicochemical properties of milk-derived exosomes and enables the in vivo study of their pharmacokinetic behavior by the non-invasive technique SPECT/CT. Our results also demonstrate the crucial role of the administration route in the biodistribution of the exosomes. In addition, our approach enabled us to confirm pharmacokinetic similarities between goat milk exosomes and synthetic liposomes [16], thus supporting the use of milk-derived exosomes as a natural substitute for synthetic nanoparticles.

## Figures and Tables

**Table 1:** Summary of pharmacokinetic properties of [^99m^Tc]-Exo for the different administration routes.

Administration Route	T_1/2_ (min)	Ex Vivo Biodistribution (Main Organs; % ID/g)
Intravenous injection	3.84	Liver (36.6 ± 7.5); Spleen (31.1 ± 11.6); Stomach (2.1 ± 0.9)
Intraperitoneal injection	15.97	Thyroid (22.0 ± 7.2); Stomach (18.1 ± 9.3); Liver (11.1 ± 5.0)
Intranasal instillation	0.77	Stomach (0.34 ± 0.11); Head (0.31 ± 0.15); Feces (0.31 ± 0.39)

## Supplementary Materials

The following are available online at https://www.mdpi.com/2079-4991/10/6/1062/s1: Table S1: Experimental data from Figure 1B. Radioefficiency of exosomes labeled with ^99m^Tc (VII) vs. ^99m^Tc (IV), Table S2: Experimental data from Figure 1C. Effect of the exosome protein concentration in the radiochemical yield, Table S3: Experimental data from Figure 1D. Determination of optimum concentration of SnCl_2_ as a reducing agent, Table S4: Experimental data from Figure 2D. Longitudinal in vitro stability study of [^99m^Tc]-Exo, Table S5: Experimental data from Figure 3C. Ex vivo biodistribution study 24 h after injection, Table S6: Experimental data from Figure 3D. In vivo blood half-life, Table S7: Experimental data from Figure 4C. Ex vivo biodistribution study 24 h after injection, Table S8: Experimental data from Figure 4D. In vivo blood half-life, Table S9: Experimental data from Figure 5C. Ex vivo biodistribution study 24 h after injection, Table S10: Experimental data from Figure 5D. In vivo blood half-life, Table S11: Experimental data from Figure 2B. Hydrodynamic size measured using dynamic light scattering, Table S12. Experimental data from Figure 2C. Radioactive HPLC chromatogram.
