# Supplementary material for: Radioactive Labeling of Milk-Derived Exosomes with 99mTc and In Vivo Tracking by SPECT Imaging
Source: Nanomaterials (Basel). 2020 May 30;10(6):1062. doi: 10.3390/nano10061062 (PMC7352469; doi:10.3390/nano10061062)
Supplement: Supplementary file 1 [file nanomaterials-10-01062-s001.zip › Supplementary files_Figure 2B_DLS available data.pdf]

Table S11. Experimental data from Figure 2B. Hydrodynamic size measured using dynamic light scattering:

**Particle size** (nm)	**1 NORMALIZED Non-labeled exosomes** (intensity, a.u.)	**2 NORMALIZED Non-labeled exosomes** (intensity, a.u.)	**3 NORMALIZED Non-labeled exosomes** (intensity, a.u.)	**1 NORMALIZED Radiolabeled exosomes** (intensity, a.u.)	**2 NORMALIZED Radiolabeled exosomes** (intensity, a.u.)	**3 NORMALIZED Radiolabeled exosomes** (intensity, a.u.)
0.4	0	0	0	0	0	0
0.463	0	0	0	0	0	0
0.536	0	0	0	0	0	0
0.621	0	0	0	0	0	0
0.719	0	0	0	0	0	0
0.833	0	0	0	0	0	0
0.965	0	0	0	0	0	0
1.12	0	0	0	0	0	0
1.29	0	0	0	0	0	0
1.5	0	0	0	0	0	0
1.74	0	0	0	0	0	0
2.01	0	0	0	0	0	0
2.33	0	0	0	0	0	0
2.7	0	0	0	0	0	0
3.12	0	0	0	0	0	0
3.62	0	0	0	0	0	0
4.19	0	0	0	0	0	0
4.85	0	0	0	0	0	0
5.61	0	0	0	0	0	0
6.5	0	0	0	0	0	0
7.53	0	0	0	0	0	0
8.72	0	0	0	0	0	0
10.1	0	0	0	0	0	0
11.7	0	0	0	0	0	0
13.545	0	0	0	0	0	0
15.686	0	0	0	0	0	0
18.166	0	0	0	0	0	0
21.037	1.355421687	0	0	0	0	0.496899225
24.363	2.801204819	0	0	0	0	0.639534884
28.214	2.876506024	0	0	0	0	0.124031008
32.674	1.72439759	0	0	0	0	0.061860465
37.84	1.129518072	1.026869759	0	2.682926829	0	2.441860465
43.821	3.576807229	7.427691254	3.220858896	10.48780488	4.130434783	9.147286822
50.748	11.04668675	20.06674653	12.01687117	23.82113821	15.07246377	21.00775194
58.771	24.20933735	37.4636317	26.56441718	41.46341463	32.46376812	37.51937984
68.061	42.03313253	56.87147014	45.21472393	60.81300813	53.62318841	56.66666667
78.82	61.9503012	75.17542358	65.08435583	78.7804878	74.63768116	75.50387597
91.28	80.50451807	89.62861544	82.79141104	92.68292683	91.30434783	90.69767442
105.709	94.12650602	98.22864967	95.18404908	100	100	99.2248062
122.42	100	100	100	99.18699187	99.27536232	100
141.772	96.72439759	95.01112442	96.29601227	91.8699187	89.13043478	91.47286822
164.183	84.67620482	84.22043471	84.66257669	77.39837398	71.5942029	76.20155039
190.137	66.02409639	69.30515146	67.11656442	59.26829268	49.85507246	56.20155039
220.194	44.36746988	52.34468595	46.77147239	40.08130081	28.55072464	35.27131783

255.002	23.99096386	35.5211364	27.2392638	22.68292683	11.8115942	17.28682171
295.312	8.802710843	20.81122711	11.87883436	9.674796748	2.384057971	5.108527132
341.995	1.09186747	9.686804724	2.82208589	2.268292683	0	0
396.058	0	2.84956358	0	0	0	0
458.666	0	0.077015232	0	0	0	0
531.172	0	0	0	0	0	0
615.139	0	0	0	0	0	0
712.379	0	0	0	0	0	0
824.992	0	0	0	0	0	0
955.406	0	0	0	0	0	0
1106.435	0	0	0	0	0	0
1281.34	0	0	0	0	0	0
1483.893	0	0	0	0	0	0
1718.466	0	0	0	0	0	0
1990.119	0	0	0	0	0	0
2304.716	0	0	0	0	0	0
2669.043	0	0	0	0	0	0
3090.964	0	0	0	0	0	0
3579.581	0	0	0	0	0	0
4145.438	0	0	0	0	0	0
4800.746	0	0	0	0	0	0
5559.644	0	0	0	0	0	0
6438.508	0	0	0	0	0	0
7456.302	0	0	0	0	0	0
8634.988	0	0	0	0	0	0
10000	0	0	0	0	0	0
824.992	0	0	0	0	0	0
955.406	0	0	0	0	0	0
1106.435	0	0	0	0	0	0
1281.34	0	0	0	0	0	0
1483.893	0	0	0	0	0	0
1718.466	0	0	0	0	0	0
1990.119	0	0	0	0	0	0
2304.716	0	0	0	0	0	0
2669.043	0	0	0	0	0	0
3090.964	0	0	0	0	0	0
3579.581	0	0	0	0	0	0
4145.438	0	0	0	0	0	0
4800.746	0	0	0	0	0	0
5559.644	0	0	0	0	0	0
6438.508	0	0	0	0	0	0
7456.302	0	0	0	0	0	0
8634.988	0	0	0	0	0	0
10000	0	0	0	0	0	0
