# Supplementary material for: Radioactive Labeling of Milk-Derived Exosomes with 99mTc and In Vivo Tracking by SPECT Imaging
Source: Nanomaterials (Basel). 2020 May 30;10(6):1062. doi: 10.3390/nano10061062 (PMC7352469; doi:10.3390/nano10061062)
Supplement: Supplementary file 1 [file nanomaterials-10-01062-s001.zip › Supplementary files_Figure 2C_HPLC available data.pdf]

Table S12. Experimental data from FIG 2C. Radioactive HPLC chromatogram:

**Time** (min)	**[99mTc]-Exo** (Intensity, normalized)	**free 99mTc** (Intensity, normalized)	**Non-labeled exosomes** (Intensity, normalized)
0	0.06565659	0.2431713	-0.1385713
0.01666667	0.05371903	0.139599	-0.1524285
0.03333333	0.07759415	0.1125734	-0.04849996
0.05	0.06565659	0.166611	0
0.06666667	0.03581268	0.09906964	0.02771427
0.08333334	0.05371903	0.1531027	0.07621423
0.1	0.08953171	0.1215834	0.1039285
0.1166667	0.03581268	0.1170762	0.09007137
0.1333333	0.04178147	0.1936275	0.07621423
0.15	0.06565659	0.1170807	0.07621423
0.1666667	0.05371903	0.1486	0.07621423
0.1833333	0.0298439	0.09906514	0.0623571
0.2	0.0298439	0.09005962	0.04849996
0.2166667	0.07162537	0.09906514	0.0623571
0.2333333	0.03581268	0.1440972	0.0623571
0.25	0.04775025	0.1080706	0.05542853
0.2666667	0.02387512	0.1260817	0.05542853
0.2833333	0.06565659	0.166611	0.0415714
0.3	0.04775025	0.1936365	0.0415714
0.3166667	0.0298439	0.1350917	0.05542853
0.3333333	0.01790634	0.1305889	0.0415714
0.35	0.04178147	0.1305889	0.0415714
0.3666667	0.02387512	0.1395945	0.0623571
0.3833333	0.04775025	0.175621	0.0623571
0.4	0.08356293	0.09456238	0.0415714
0.4166667	0.03581268	0.166611	0.02771427
0.4333333	0.08953171	0.1576055	0.0415714
0.45	0.04178147	0.1395945	0.03464283
0.4666667	0.04775025	0.1125779	0.0207857
0.4833333	0.0298439	0.1035679	0.006928566
0.5	0.0298439	0.166611	-0.01385713
0.5166667	0.04775025	0.1666155	-0.0415714
0.5333334	0.0298439	0.1350917	-0.05542853
0.55	0.03581268	0.1125779	-0.05542853
0.5666667	0.04178147	0.1350917	-0.05542853
0.5833333	0.03581268	0.1711182	-0.05542853
0.6	0.0298439	0.1350917	-0.0623571
0.6166667	0.08356293	0.1080751	-0.06928567
0.6333333	0.0298439	0.1486	-0.0623571
0.65	0.005968781	0.1711182	-0.04849996
0.6666667	0.03581268	0.08555687	-0.05542853

0.6833333	0.04775025	0.1125734	-0.06928567
0.7	0.04775025	0.1215834	-0.07621423
0.7166666	0.05371903	0.1486	-0.0623571
0.7333333	0.06565659	0.1170807	-0.07621423
0.75	0.04775025	0.1486	-0.1039285
0.7666667	0.04775025	0.1440972	-0.09699993
0.7833334	0.04178147	0.1260862	-0.07621423
0.8	0.04178147	0.1215834	-0.07621423
0.8166667	0.06565659	0.1260862	-0.09699993
0.8333333	0.03581268	0.1080706	-0.1108571
0.85	0.04775025	0.1350872	-0.09007137
0.8666667	0.01193756	0.1395945	-0.08314279
0.8833333	0.04775025	0.1395945	-0.1177856
0.9	0.05968781	0.1260817	-0.1385713
0.9166667	0.05371903	0.1801238	-0.1454999
0.9333333	0.01790634	0.1395945	-0.1454999
0.95	0.03581268	0.1305889	-0.1524285
0.9666666	0.04178147	0.1170807	-0.1662856
0.9833333	0.04178147	0.1125734	-0.1732142
1	0.0298439	0.1170807	-0.159357
1.016667	0.0298439	0.1125734	-0.1524285
1.033333	0.01790634	0.1170762	-0.1524285
1.05	0.04178147	0.1350917	-0.1524285
1.066667	0.0298439	0.1170807	-0.159357
1.083333	0.0298439	0.1305889	-0.1801427
1.1	0.04178147	0.1260817	-0.1870713
1.116667	0.01193756	0.1170807	-0.2009284
1.133333	0.0298439	0.1170762	-0.2147856
1.15	0.05968781	0.1711137	-0.2286427
1.166667	0.05371903	0.1711182	-0.2355713
1.183333	0.04178147	0.15761	-0.2286427
1.2	0.05968781	0.09906514	-0.2147856
1.216667	0.0298439	0.157601	-0.2286427
1.233333	0.02387512	0.1441017	-0.2424998
1.25	0.05371903	0.1260862	-0.2286427
1.266667	0.01790634	0.1215834	-0.2147856
1.283333	0.04775025	0.1576055	-0.2286427
1.3	0.05371903	0.1531027	-0.256357
1.316667	0.03581268	0.1215834	-0.2494284
1.333333	0.04775025	0.1801238	-0.2286427
1.35	0.0298439	0.1350917	-0.2355713
1.366667	0.03581268	0.1531027	-0.256357
1.383333	0.005968781	0.1441017	-0.2771427
1.4	0.05371903	0.09906514	-0.2840712
1.416667	0.04775025	0.1486	-0.2632855
1.433333	0.04775025	0.1486	-0.256357
1.45	0.04775025	0.1035679	-0.2702141

1.466667	0.04775025	0.1260862	-0.2771427
1.483333	0.05371903	0.1305844	-0.2702141
1.5	0.03581268	0.1486	-0.2771427
1.516667	0.04775025	0.1350917	-0.2840712
1.533333	0.0298439	0.1350917	-0.2702141
1.55	0.03581268	0.08555687	-0.2771427
1.566667	0.05371903	0.1080706	-0.3048569
1.583333	0.03581268	0.1395945	-0.3394998
1.6	0.0298439	0.09906514	-0.3533569
1.616667	0.06565659	0.166611	-0.3602855
1.633333	0.03581268	0.1440972	-0.3602855
1.65	0.04775025	0.1260862	-0.3602855
1.666667	0.0298439	0.1531027	-0.367214
1.683333	0.01790634	0.1440972	-0.367214
1.7	0.01790634	0.1215834	-0.3602855
1.716667	0.01193756	0.166611	-0.3602855
1.733333	0.01790634	0.1350917	-0.3879997
1.75	0.01790634	0.1576055	-0.4018569
1.766667	0.05968781	0.1576055	-0.4018569
1.783333	0.04178147	0.1350917	-0.415714
1.8	0.05968781	0.1395945	-0.4364997
1.816667	0.03581268	0.166611	-0.4434282
1.833333	0.05968781	0.1531072	-0.4364997
1.85	0.04178147	0.09456238	-0.4364997
1.866667	0.01790634	0.1440972	-0.4364997
1.883333	0.01790634	0.1621082	-0.4364997
1.9	0.03581268	0.1125779	-0.4434282
1.916667	0.04775025	0.1215834	-0.4503568
1.933333	0.04775025	0.1260817	-0.4434282
1.95	0.0298439	0.1215834	-0.4503568
1.966667	0.05371903	0.1035724	-0.4503568
1.983333	0.04178147	0.1035679	-0.4503568
2	0.04775025	0.1486	-0.4572854
2.016667	0.03581268	0.1350917	-0.464214
2.033333	0.01790634	0.1260817	-0.4711425
2.05	0.05968781	0.1350917	-0.4988568
2.066667	0.05371903	0.1486	-0.4988568
2.083333	0.02387512	0.1621127	-0.5127139
2.1	0.04775025	0.09005962	-0.5404282
2.116667	0.04775025	0.1260862	-0.5612139
2.133333	0.04775025	0.1125734	-0.5889282
2.15	0.0298439	0.1035679	-0.6097139
2.166667	0.0298439	0.1486	-0.5958567
2.183333	0.03581268	0.1125779	-0.575071
2.2	0.02387512	0.08555687	-0.5889282
2.216667	0.02387512	0.1260817	-0.5958567
2.233333	0.02387512	0.08105411	-0.5889282

2.25	0.0298439	0.1215789	-0.6097139
2.266667	0.04775025	0.1531027	-0.6304995
2.283333	0.01790634	0.1981393	-0.6443567
2.3	0.01790634	0.09906514	-0.6651424
2.316667	0.04775025	0.1125734	-0.6859281
2.333333	0.04178147	0.1486	-0.6859281
2.35	0.0298439	0.1350917	-0.6928567
2.366667	0.06565659	0.1215834	-0.6859281
2.383333	0.05968781	0.166611	-0.6859281
2.4	0.06565659	0.1305889	-0.6997852
2.416667	0.05968781	0.139599	-0.6928567
2.433333	0.04775025	0.1080706	-0.6928567
2.45	0.04775025	0.1260817	-0.734428
2.466667	0.04775025	0.1440972	-0.7690709
2.483333	0.03581268	0.193632	-0.782928
2.5	0.0298439	0.1801283	-0.7759994
2.516667	0.04775025	0.1260862	-0.7690709
2.533333	0.04775025	0.1350917	-0.7690709
2.55	0.04178147	0.08555687	-0.7759994
2.566667	0.04178147	0.1756165	-0.7967851
2.583333	0.02387512	0.1576055	-0.8244994
2.6	0.04178147	0.1170807	-0.8452851
2.616667	0.03581268	0.1440972	-0.8452851
2.633333	0.0298439	0.1305889	-0.8522137
2.65	0.0298439	0.1711182	-0.8660708
2.666667	0.04775025	0.1801238	-0.8591422
2.683333	0.0298439	0.1486	-0.8591422
2.7	0.05371903	0.1441017	-0.8799279
2.716667	0.0298439	0.1125734	-0.9076422
2.733333	0.04178147	0.1260862	-0.9284279
2.75	0.0298439	0.0720486	-0.9422851
2.766667	0.04775025	0.157601	-0.9630708
2.783333	0.03581268	0.1531072	-0.9769279
2.8	0.02387512	0.193632	-0.9838564
2.816667	0.03581268	0.1350917	-0.990785
2.833333	0.01790634	0.1621127	-1.011571
2.85	0.03581268	0.1305889	-1.025428
2.866667	0.02387512	0.1531027	-1.025428
2.883333	0.04178147	0.1260862	-1.018499
2.9	0.01193756	0.1170762	-1.004642
2.916667	0.005968781	0.1305889	-1.018499
2.933333	0.0298439	0.1350917	-1.039285
2.95	0.04775025	0.1486045	-1.039285
2.966667	0.04178147	0.1260862	-1.046214
2.983333	0.0298439	0.08555687	-1.053142
3	0.04178147	0.1350872	-1.066999
3.016667	0.05371903	0.1531072	-1.073928

3.033333	0.03581268	0.1170762	-1.087785
3.05	0.03581268	0.15761	-1.129356
3.066667	0.02387512	0.1305889	-1.157071
3.083333	0.04775025	0.09906514	-1.170928
3.1	0.0298439	0.1215834	-1.177856
3.116667	0.03581268	0.1576055	-1.170928
3.133333	0.02387512	0.1080751	-1.170928
3.15	0.03581268	0.1170762	-1.184785
3.166667	0.0298439	0.1440972	-1.198642
3.183333	0.03581268	0.1080751	-1.212499
3.2	0.03581268	0.1035679	-1.233285
3.216667	0.04178147	0.1440972	-1.254071
3.233333	0.01790634	0.1305889	-1.254071
3.25	0.04775025	0.1260817	-1.254071
3.266667	0.02387512	0.1080706	-1.288713
3.283333	0.02387512	0.1170762	-1.330285
3.3	0.01790634	0.1170762	-1.337213
3.316667	0.005968781	0.1305889	-1.30257
3.333333	0.04178147	0.13959	-1.295642
3.35	0.06565659	0.1711137	-1.330285
3.366667	0.04178147	0.15761	-1.351071
3.383333	0.04775025	0.1576055	-1.357999
3.4	0.04178147	0.1080751	-1.371856
3.416667	0.03581268	0.1531027	-1.385713
3.433333	0.01193756	0.1215834	-1.385713
3.45	0.05968781	0.1395945	-1.39957
3.466667	0.04178147	0.1486	-1.427285
3.483333	0.03581268	0.1260817	-1.44807
3.5	0.04775025	0.1711182	-1.461928
3.516667	0.04178147	0.1260862	-1.44807
3.533333	0.03581268	0.1215834	-1.44807
3.55	0.04775025	0.1215834	-1.461928
3.566667	0.06565659	0.09005962	-1.461928
3.583333	0.005968781	0.1035679	-1.482713
3.6	0.04178147	0.1395945	-1.49657
3.616667	0.04178147	0.1440972	-1.475785
3.633333	0.02387512	0.1170807	-1.461928
3.65	0.04178147	0.1576055	-1.475785
3.666667	0.0298439	0.1531072	-1.503499
3.683333	0.0298439	0.1215834	-1.517356
3.7	0.01790634	0.1350917	-1.524285
3.716667	0.06565659	0.1260862	-1.538142
3.733333	0.0298439	0.1260862	-1.551999
3.75	0.03581268	0.06754585	-1.551999
3.766667	0.0298439	0.1350917	-1.558927
3.783333	0.03581268	0.1350917	-1.558927
3.8	0.05371903	0.1576055	-1.558927

3.816667	0.02387512	0.1260862	-1.572785
3.833333	0.05371903	0.1576055	-1.586642
3.85	0.05968781	0.1395945	-1.59357
3.866667	0.04178147	0.07655136	-1.572785
3.883333	0.0298439	0.1215789	-1.565856
3.9	0.02387512	0.1035679	-1.600499
3.916667	0.0298439	0.09005962	-1.621285
3.933333	0.04775025	0.1621082	-1.607427
3.95	0.04775025	0.1035724	-1.607427
3.966667	0.0298439	0.1215834	-1.628213
3.983333	0.04178147	0.1350917	-1.648999
4	0.0298439	0.1215834	-1.635142
4.016667	0.06565659	0.1531027	-1.635142
4.033333	0.01790634	0.1395945	-1.662856
4.05	0.04775025	0.08105411	-1.676713
4.066667	0.05968781	0.09906514	-1.662856
4.083333	0.01193756	0.1305889	-1.669785
4.1	0.01193756	0.1440972	-1.683642
4.116667	0.0298439	0.09005962	-1.676713
4.133333	0.04178147	0.09906514	-1.676713
4.15	0.0298439	0.1215789	-1.676713
4.166667	0.04178147	0.1486	-1.676713
4.183333	0.005968781	0.1305889	-1.662856
4.2	0.0298439	0.1170807	-1.635142
4.216667	0.01790634	0.1125734	-1.614356
4.233333	0.04178147	0.1305889	-1.607427
4.25	0.04178147	0.1711137	-1.614356
4.266667	0.02387512	0.1035724	-1.635142
4.283333	0.03581268	0.1215789	-1.64207
4.3	0.03581268	0.1621082	-1.635142
4.316667	0.0298439	0.1440972	-1.628213
4.333333	0.07162537	0.1260862	-1.64207
4.35	0.02387512	0.1486	-1.64207
4.366667	0.06565659	0.1350917	-1.64207
4.383333	0.0298439	0.1080751	-1.635142
4.4	0.07162537	0.1440972	-1.600499
4.416667	0.03581268	0.1621082	-1.565856
4.433333	0.04178147	0.1170807	-1.558927
4.45	0.05968781	0.1801238	-1.565856
4.466667	0.02387512	0.1395945	-1.572785
4.483333	0.03581268	0.1350917	-1.579713
4.5	0.04178147	0.09906514	-1.565856
4.516667	0.0298439	0.09456238	-1.558927
4.533333	0.03581268	0.1305844	-1.572785
4.55	0.02387512	0.1305889	-1.600499
4.566667	0.03581268	0.1215789	-1.614356
4.583333	0.01790634	0.1260862	-1.621285

4.6	0.05968781	0.1350917	-1.614356
4.616667	0.02387512	0.1350917	-1.600499
4.633333	0.02387512	0.1440972	-1.579713
4.65	0.05371903	0.1350917	-1.565856
4.666667	0.03581268	0.1350917	-1.558927
4.683333	0.05968781	0.1215789	-1.54507
4.7	0.01790634	0.1215834	-1.538142
4.716667	0.05371903	0.1170762	-1.538142
4.733333	0.05968781	0.1035679	-1.54507
4.75	0.04178147	0.1305889	-1.54507
4.766667	0.04178147	0.1395945	-1.572785
4.783333	0.04775025	0.09005962	-1.579713
4.8	0.01790634	0.1801192	-1.572785
4.816667	0.05371903	0.1260862	-1.565856
4.833333	0.0298439	0.1756165	-1.565856
4.85	0.05968781	0.1125779	-1.558927
4.866667	0.01790634	0.1531027	-1.551999
4.883333	0.05968781	0.1621127	-1.551999
4.9	0.07162537	0.1170807	-1.551999
4.916667	0.0298439	0.1395945	-1.54507
4.933333	0.0298439	0.1395945	-1.538142
4.95	0.04775025	0.1621082	-1.531213
4.966667	0.005968781	0.1215834	-1.531213
4.983333	0.04775025	0.1125734	-1.531213
5	0.0298439	0.1080706	-1.531213
5.016667	0.04178147	0.09906514	-1.524285
5.033333	0.0298439	0.1260817	-1.517356
5.05	0.05968781	0.1486	-1.517356
5.066667	0.04178147	0.1125779	-1.524285
5.083333	0.05371903	0.1621082	-1.517356
5.1	0.03581268	0.1080706	-1.517356
5.116667	0.06565659	0.1125734	-1.524285
5.133333	0.04178147	0.1440972	-1.531213
5.15	0.01790634	0.1215834	-1.538142
5.166667	0.02387512	0.1215789	-1.517356
5.183333	0.0298439	0.1350917	-1.489642
5.2	0.07759415	0.1260862	-1.489642
5.216667	0.05968781	0.1395945	-1.503499
5.233333	0.02387512	0.1621127	-1.517356
5.25	0.05968781	0.1035679	-1.510427
5.266667	0.0298439	0.1260817	-1.503499
5.283333	0.04775025	0.1080706	-1.49657
5.3	0.04178147	0.1395945	-1.503499
5.316667	0.03581268	0.1395945	-1.531213
5.333333	0.01790634	0.1035679	-1.538142
5.35	0.06565659	0.1170762	-1.524285
5.366667	0.04178147	0.1486	-1.517356

5.383333	0.0298439	0.1035679	-1.524285
5.4	0.01790634	0.1305889	-1.538142
5.416667	0.0298439	0.1576055	-1.551999
5.433333	0.03581268	0.1846265	-1.565856
5.45	0.07162537	0.1125779	-1.565856
5.466667	0.04178147	0.1621082	-1.565856
5.483333	0.04775025	0.1080706	-1.565856
5.5	0.0298439	0.1305844	-1.572785
5.516667	0.04775025	0.1531027	-1.586642
5.533333	0.0298439	0.1395945	-1.586642
5.55	0.03581268	0.139599	-1.586642
5.566667	0.06565659	0.1576055	-1.565856
5.583333	0.0298439	0.1215834	-1.558927
5.6	0.03581268	0.1395945	-1.572785
5.616667	0.02387512	0.1801238	-1.586642
5.633333	0.03581268	0.1305889	-1.600499
5.65	0.01790634	0.1125779	-1.621285
5.666667	0.02387512	0.1350872	-1.635142
5.683333	0.05968781	0.1441017	-1.648999
5.7	0.0298439	0.09456238	-1.648999
5.716667	0.02387512	0.1080706	-1.655927
5.733333	0.02387512	0.1125734	-1.662856
5.75	0.03581268	0.1531027	-1.669785
5.766667	0.0298439	0.1531072	-1.683642
5.783333	0.05371903	0.09005962	-1.704427
5.8	0.02387512	0.1350872	-1.718284
5.816667	0.04775025	0.1395945	-1.711356
5.833333	0.0298439	0.1260862	-1.718284
5.85	0.01790634	0.1576055	-1.725213
5.866667	0.0298439	0.1395945	-1.725213
5.883333	0.07162537	0.1440972	-1.718284
5.9	0.05968781	0.1395945	-1.725213
5.916667	0.0298439	0.1440972	-1.759856
5.933333	0.05371903	0.1350917	-1.780642
5.95	0.04775025	0.1486045	-1.773713
5.966667	0.04178147	0.15761	-1.759856
5.983333	0.03581268	0.1125779	-1.752927
6	0.03581268	0.166611	-1.766784
6.016667	0.04775025	0.1170807	-1.780642
6.033333	0.04178147	0.1170807	-1.808356
6.05	0.0298439	0.09005962	-1.815284
6.066667	0.05371903	0.1260817	-1.808356
6.083333	0.05371903	0.1260817	-1.815284
6.1	0.04178147	0.1981393	-1.842999
6.116667	0.04775025	0.1305889	-1.849927
6.133333	0.05968781	0.1576055	-1.849927
6.15	0.03581268	0.1440972	-1.849927

6.166667	0.0298439	0.1125734	-1.870713
6.183333	0.04775025	0.1440972	-1.891499
6.2	0.05371903	0.1215834	-1.898427
6.216667	0.02387512	0.1395945	-1.891499
6.233333	0.0298439	0.1621127	-1.877642
6.25	0.04178147	0.1531027	-1.88457
6.266667	0.03581268	0.1260862	-1.905356
6.283333	0.05968781	0.1035679	-1.919213
6.3	0.05371903	0.1621082	-1.946927
6.316667	0.0298439	0.1215834	-1.960784
6.333333	0.03581268	0.1350917	-1.967713
6.35	0.04775025	0.1576055	-1.98157
6.366667	0.03581268	0.1441017	-1.995427
6.383333	0.0298439	0.1035679	-2.009284
6.4	0.08356293	0.1350872	-2.023141
6.416667	0.03581268	0.09906514	-2.03007
6.433333	0.04178147	0.1305844	-2.023141
6.45	0.04178147	0.1215834	-2.009284
6.466667	0.04775025	0.1395945	-2.016213
6.483333	0.02387512	0.1486	-2.050856
6.5	0.04775025	0.1035679	-2.07857
6.516667	0.0298439	0.1215789	-2.085499
6.533333	0.005968781	0.1260862	-2.092427
6.55	0.02387512	0.1486	-2.120141
6.566667	0.04178147	0.1350917	-2.133998
6.583333	0.03581268	0.13959	-2.12707
6.6	0.02387512	0.1125779	-2.154784
6.616667	0.06565659	0.1440927	-2.17557
6.633333	0.03581268	0.1215834	-2.182498
6.65	0.02387512	0.1170807	-2.168641
6.666667	0.02387512	0.1215834	-2.161713
6.683333	0.01790634	0.1260862	-2.168641
6.7	0.03581268	0.1350917	-2.196356
6.716667	0.0298439	0.1035679	-2.217141
6.733333	0.01193756	0.1486	-2.22407
6.75	0.04178147	0.1215789	-2.230999
6.766667	0.01790634	0.1621082	-2.244856
6.783333	0.05371903	0.1215834	-2.265641
6.8	0.05371903	0.1711137	-2.279498
6.816667	0.04775025	0.1125779	-2.258713
6.833333	0.04178147	0.1170762	-2.244856
6.85	0.05371903	0.09456238	-2.258713
6.866667	0.02387512	0.1576055	-2.265641
6.883333	0.05371903	0.1125734	-2.279498
6.9	0.0298439	0.1260862	-2.300284
6.916667	0.04775025	0.1260862	-2.314141
6.933333	0.02387512	0.166611	-2.341856

6.95	0.05968781	0.139599	-2.355713
6.966667	0.02387512	0.1260817	-2.348784
6.983333	0.05968781	0.1486	-2.355713
7	0.05968781	0.1215834	-2.362641
7.016667	0.04178147	0.1080706	-2.376498
7.033333	0.02387512	0.1711182	-2.404212
7.05	0.01790634	0.1125779	-2.404212
7.066667	0.0298439	0.1305889	-2.397284
7.083333	0.04178147	0.1125734	-2.411141
7.1	0.04178147	0.1576055	-2.424998
7.116667	0.01193756	0.1801238	-2.438855
7.133333	0.04178147	0.1395945	-2.459641
7.15	0.04178147	0.1801238	-2.480427
7.166667	0.03581268	0.1215834	-2.508141
7.183333	0.04178147	0.1395945	-2.508141
7.2	0.05968781	0.09005962	-2.51507
7.216667	0.0298439	0.1621082	-2.528927
7.233333	0.0298439	0.1215834	-2.535855
7.25	0.04775025	0.09005962	-2.51507
7.266667	0.07162537	0.1170762	-2.487355
7.283333	0.04775025	0.1711137	-2.473498
7.3	0.04775025	0.1801283	-2.494284
7.316667	0.01790634	0.09906514	-2.51507
7.333333	0.04775025	0.1305889	-2.521998
7.35	0.04178147	0.1440972	-2.542784
7.366667	0.08356293	0.1350917	-2.556641
7.383333	0.05371903	0.166611	-2.56357
7.4	0.03581268	0.1486045	-2.556641
7.416667	0.0298439	0.1531027	-2.556641
7.433333	0.02387512	0.1215834	-2.577427
7.45	0.03581268	0.1576055	-2.618998
7.466667	0.04775025	0.1125734	-2.632855
7.483333	0.03581268	0.1531072	-2.639784
7.5	0.04775025	0.1486	-2.625927
7.516667	0.04178147	0.139599	-2.618998
7.533333	0.04775025	0.1801238	-2.653641
7.55	0.02387512	0.1350917	-2.702141
7.566667	0.04775025	0.1215834	-2.709069
7.583333	0.0298439	0.1801238	-2.702141
7.6	0.05371903	0.1215834	-2.715998
7.616667	0.05371903	0.1215789	-2.729855
7.633333	0.05371903	0.1666155	-2.715998
7.65	0.01790634	0.1531072	-2.695212
7.666667	0.05371903	0.1395945	-2.702141
7.683333	0.03581268	0.1621082	-2.743712
7.7	0.03581268	0.1395945	-2.75757
7.716667	0.04775025	0.1486	-2.750641

7.733333	0.04178147	0.1215834	-2.750641
7.75	0.02387512	0.09906514	-2.764498
7.766667	0.03581268	0.1035679	-2.792212
7.783333	0.0298439	0.1215834	-2.806069
7.8	0.0298439	0.1576055	-2.812998
7.816667	0.0298439	0.1035724	-2.819927
7.833333	0.06565659	0.1215789	-2.806069
7.85	0.07162537	0.1260862	-2.819927
7.866667	0.03581268	0.1260862	-2.847641
7.883333	0.05371903	0.1395945	-2.861498
7.9	0.01790634	0.1035679	-2.861498
7.916667	0.04178147	0.1170762	-2.861498
7.933333	0.05968781	0.1440972	-2.847641
7.95	0.05968781	0.1170807	-2.833784
7.966667	0.01193756	0.1125734	-2.840712
7.983333	0.04178147	0.184622	-2.875355
8	0.05371903	0.1305889	-2.896141
8.016666	0.05968781	0.1080706	-2.889212
8.033334	0.04775025	0.1035679	-2.875355
8.05	0.05371903	0.1395945	-2.889212
8.066667	0.0298439	0.1035724	-2.903069
8.083333	0.05968781	0.1035679	-2.875355
8.1	0.04775025	0.1576055	-2.847641
8.116667	0.05371903	0.1215834	-2.854569
8.133333	0.05968781	0.1395945	-2.889212
8.15	0.06565659	0.1395945	-2.916926
8.166667	0.0298439	0.1350917	-2.951569
8.183333	0.03581268	0.1395945	-2.979284
8.2	0.03581268	0.1486	-2.979284
8.216666	0.05968781	0.1486045	-2.972355
8.233334	0.03581268	0.1080706	-2.979284
8.25	0.07759415	0.1305889	-2.993141
8.266666	0.07162537	0.08555687	-2.986212
8.283334	0.0298439	0.1350917	-2.958498
8.3	0.05968781	0.1756165	-2.958498
8.316667	0.04178147	0.1035679	-3.000069
8.333333	0.04775025	0.1305889	-3.013927
8.35	0.03581268	0.1440972	-2.993141
8.366667	0.04178147	0.1756165	-3.006998
8.383333	0.08356293	0.1441017	-3.041641
8.4	0.04775025	0.0720486	-3.034712
8.416667	0.07162537	0.1035679	-3.013927
8.433333	0.03581268	0.09906514	-3.000069
8.45	0.05968781	0.1576055	-2.993141
8.466666	0.08356293	0.1486	-3.000069
8.483334	0.04178147	0.1035679	-3.027784
8.5	0.03581268	0.1395945	-3.069355

8.516666	0.06565659	0.1350917	-3.103998
8.533334	0.04775025	0.1711137	-3.103998
8.55	0.02387512	0.1350917	-3.090141
8.566667	0.05371903	0.08105411	-3.110926
8.583333	0.06565659	0.1440972	-3.138641
8.6	0.03581268	0.1846265	-3.152498
8.616667	0.06565659	0.1215834	-3.145569
8.633333	0.06565659	0.1080706	-3.138641
8.65	0.05968781	0.1440972	-3.138641
8.666667	0.02387512	0.1576055	-3.131712
8.683333	0.08953171	0.1531027	-3.131712
8.7	0.0298439	0.1350917	-3.152498
8.716666	0.03581268	0.1440972	-3.173283
8.733334	0.08953171	0.1305889	-3.159426
8.75	0.05968781	0.09005962	-3.152498
8.766666	0.04775025	0.1215789	-3.159426
8.783334	0.05968781	0.1350917	-3.166355
8.8	0.02387512	0.1035724	-3.166355
8.816667	0.04178147	0.1350917	-3.173283
8.833333	0.03581268	0.1305889	-3.18714
8.85	0.04178147	0.175621	-3.194069
8.866667	0.05371903	0.08555687	-3.18714
8.883333	0.02387512	0.1260817	-3.166355
8.9	0.05371903	0.1350917	-3.159426
8.916667	0.04775025	0.0720486	-3.173283
8.933333	0.04775025	0.166611	-3.200998
8.95	0.04775025	0.1260862	-3.235641
8.966666	0.04178147	0.1305844	-3.249498
8.983334	0.05371903	0.1486045	-3.249498
9	0.05968781	0.1486	-3.242569
9.016666	0.05968781	0.1170807	-3.242569
9.033334	0.03581268	0.1440972	-3.256426
9.05	0.06565659	0.1125734	-3.277212
9.066667	0.04178147	0.1170762	-3.297998
9.083333	0.05371903	0.1215789	-3.291069
9.1	0.05968781	0.1486	-3.291069
9.116667	0.01790634	0.1080706	-3.297998
9.133333	0.05371903	0.1305889	-3.304926
9.15	0.02387512	0.09456238	-3.311855
9.166667	0.02387512	0.09456238	-3.318783
9.183333	0.005968781	0.1576055	-3.325712
9.2	0.04775025	0.1260862	-3.325712
9.216666	0.05968781	0.09906514	-3.33264
9.233334	0.04178147	0.1125734	-3.339569
9.25	0.05968781	0.1215834	-3.339569
9.266666	0.05371903	0.08105411	-3.353426
9.283334	0.02387512	0.1260862	-3.374212

9.3	0.04775025	0.09456238	-3.388069
9.316667	0.04775025	0.1260817	-3.388069
9.333333	0.02387512	0.1350917	-3.388069
9.35	0.05968781	0.1260817	-3.394998
9.366667	0.04775025	0.1080751	-3.388069
9.383333	0.05371903	0.09005962	-3.388069
9.4	0.04775025	0.1531027	-3.408855
9.416667	0.05968781	0.1350917	-3.42964
9.433333	0.01193756	0.1440972	-3.436569
9.45	0.04775025	0.1395945	-3.450426
9.466666	0.0298439	0.1215834	-3.471212
9.483334	0.04178147	0.1170762	-3.471212
9.5	0.02387512	0.1350917	-3.47814
9.516666	0.02387512	0.1395945	-3.498926
9.533334	0.0298439	0.1305889	-3.512783
9.55	0.05968781	0.1440972	-3.498926
9.566667	0.03581268	0.1170762	-3.47814
9.583333	0.01790634	0.1260862	-3.485069
9.6	0.05371903	0.1215834	-3.498926
9.616667	0.01193756	0.1260862	-3.512783
9.633333	0.01790634	0.166611	-3.505855
9.65	0.02387512	0.1711182	-3.498926
9.666667	0.04178147	0.08555687	-3.519712
9.683333	0.04178147	0.1305889	-3.547426
9.7	0.06565659	0.1260862	-3.547426
9.716666	0.0298439	0.1486	-3.52664
9.733334	0.01790634	0.1170807	-3.505855
9.75	0.04178147	0.1305889	-3.505855
9.766666	0.04178147	0.1395945	-3.519712
9.783334	0.02387512	0.1576055	-3.540498
9.8	0.01790634	0.1440972	-3.57514
9.816667	0.0298439	0.1350917	-3.582069
9.833333	0.0298439	0.1350917	-3.582069
9.85	0.0298439	0.1035679	-3.588997
9.866667	0.04178147	0.1035679	-3.588997
9.883333	0.05968781	0.1305889	-3.588997
9.9	0.0298439	0.1170762	-3.588997
9.916667	0.07759415	0.1350917	-3.602854
9.933333	0.03581268	0.1260862	-3.62364
9.95	0.03581268	0.15761	-3.62364
9.966666	0.03581268	0.1440972	-3.630569
9.983334	0.05371903	0.1035679	-3.651355
10	0.02387512	0.184622	-3.644426
10.01667	0.02387512	0.1170807	-3.637497
10.03333	0.04775025	0.1395945	-3.644426
10.05	0.04178147	0.1215834	-3.637497
10.06667	0.0298439	0.1170762	-3.62364

10.08333	0.02387512	0.1440972	-3.62364
10.1	0.01790634	0.1035679	-3.637497
10.11667	0.04775025	0.1125734	-3.651355
10.13333	0.04178147	0.1801238	-3.679069
10.15	0.0298439	0.1170807	-3.692926
10.16667	0.04775025	0.1756165	-3.67214
10.18333	0.05371903	0.1170807	-3.658283
10.2	0.05371903	0.1035679	-3.679069
10.21667	0.04775025	0.1666155	-3.706783
10.23333	0.03581268	0.1350917	-3.72064
10.25	0.04775025	0.1486	-3.727569
10.26667	0.0298439	0.1260817	-3.727569
10.28333	0.03581268	0.1350917	-3.727569
10.3	0.03581268	0.1305889	-3.727569
10.31667	0.01790634	0.1395945	-3.741426
10.33333	0.02387512	0.1350917	-3.734497
10.35	0.05371903	0.1440972	-3.727569
10.36667	0.02387512	0.1801238	-3.734497
10.38333	0.0298439	0.1711182	-3.741426
10.4	0.05371903	0.1080706	-3.76914
10.41667	0.05371903	0.1440972	-3.776069
10.43333	0.04775025	0.09005962	-3.755283
10.45	0.02387512	0.1125734	-3.727569
10.46667	0.0298439	0.09906514	-3.734497
10.48333	0.03581268	0.1305889	-3.748354
10.5	0.05968781	0.1801192	-3.748354
10.51667	0.04178147	0.1035724	-3.734497
10.53333	0.05968781	0.1305889	-3.72064
10.55	0.03581268	0.1350917	-3.734497
10.56667	0.04178147	0.1440972	-3.76914
10.58333	0.03581268	0.166611	-3.803783
10.6	0.0298439	0.1350917	-3.810712
10.61667	0.05371903	0.1080706	-3.803783
10.63333	0.0298439	0.1125734	-3.803783
10.65	0.05371903	0.1440972	-3.810712
10.66667	0.03581268	0.1936365	-3.803783
10.68333	0.04178147	0.1215834	-3.776069
10.7	0.0298439	0.1215834	-3.76914
10.71667	0.02387512	0.09005962	-3.782997
10.73333	0.05968781	0.1260817	-3.789926
10.75	0.01790634	0.1350917	-3.782997
10.76667	0.04775025	0.1170807	-3.796854
10.78333	0.02387512	0.1350917	-3.803783
10.8	0.01790634	0.1035679	-3.796854
10.81667	0.04178147	0.1305844	-3.796854
10.83333	0.04775025	0.1440972	-3.810712
10.85	0.05968781	0.202642	-3.803783

10.86667	0.0298439	0.1531072	-3.803783
10.88333	0.02387512	0.1486045	-3.81764
10.9	0.03581268	0.1350917	-3.81764
10.91667	0.01193756	0.1125779	-3.81764
10.93333	0.05968781	0.1170762	-3.824569
10.95	0.05968781	0.1576055	-3.831497
10.96667	0.05371903	0.1531072	-3.845354
10.98333	0.0298439	0.1260862	-3.859211
11	0.03581268	0.1080706	-3.859211
11.01667	0.0298439	0.1440972	-3.86614
11.03333	0.01790634	0.1080706	-3.886926
11.05	0.05371903	0.1170762	-3.900783
11.06667	0.03581268	0.1125734	-3.900783
11.08333	0.07162537	0.1080706	-3.900783
11.1	0.05968781	0.166611	-3.907712
11.11667	0.04775025	0.1305889	-3.921569
11.13333	0.0298439	0.1260817	-3.921569
11.15	0.03581268	0.1350917	-3.900783
11.16667	0.04178147	0.1215834	-3.900783
11.18333	0.03581268	0.1756165	-3.907712
11.2	0.06565659	0.1215834	-3.907712
11.21667	0.05371903	0.1305889	-3.900783
11.23333	0.07162537	0.1215789	-3.91464
11.25	0.06565659	0.1215834	-3.921569
11.26667	0.05371903	0.1260817	-3.928497
11.28333	0.02387512	0.1125734	-3.935426
11.3	0.04178147	0.1486	-3.942354
11.31667	0.06565659	0.1125779	-3.935426
11.33333	0.04178147	0.1305844	-3.900783
11.35	0.03581268	0.1531072	-3.886926
11.36667	0.0298439	0.1035724	-3.893854
11.38333	0.04775025	0.1350872	-3.907712
11.4	0.0298439	0.1395945	-3.928497
11.41667	0.02387512	0.1125779	-3.942354
11.43333	0.0298439	0.1891293	-3.935426
11.45	0.02387512	0.1305889	-3.921569
11.46667	0.01193756	0.1711182	-3.91464
11.48333	0.05968781	0.1080706	-3.935426
11.5	0.03581268	0.1486	-3.96314
11.51667	0.04178147	0.1486045	-3.983926
11.53333	0.04178147	0.175621	-3.983926
11.55	0.02387512	0.1666155	-3.956212
11.56667	0.0298439	0.1080706	-3.935426
11.58333	0.04178147	0.09906514	-3.935426
11.6	0.05968781	0.1395945	-3.942354
11.61667	0.05968781	0.1305889	-3.949283
11.63333	0.01193756	0.1666155	-3.949283

11.65	0.0298439	0.1350917	-3.970069
11.66667	0.06565659	0.08105411	-3.990854
11.68333	0.05968781	0.1440927	-4.004712
11.7	0.03581268	0.15761	-4.025497
11.71667	0.04178147	0.1395945	-4.032426
11.73333	0.05371903	0.09456238	-4.018569
11.75	0.03581268	0.1035679	-3.990854
11.76667	0.04775025	0.1260862	-3.990854
11.78333	0.02387512	0.1531027	-3.997783
11.8	0.03581268	0.1260862	-4.004712
11.81667	0.03581268	0.1486	-4.018569
11.83333	0.02387512	0.1486045	-4.018569
11.85	0.03581268	0.1576055	-4.01164
11.86667	0.05968781	0.1486	-3.990854
11.88333	0.04178147	0.1395945	-3.970069
11.9	0.05371903	0.1260862	-3.990854
11.91667	0.03581268	0.1891293	-4.01164
11.93333	0.0298439	0.09456238	-4.01164
11.95	0.02387512	0.1395945	-4.01164
11.96667	0.0298439	0.1305889	-4.018569
11.98333	0.02387512	0.1350917	-4.018569
12	0.04178147	0.1350917	-3.997783
12.01667	0.04775025	0.1846265	-3.997783
12.03333	0.0298439	0.1440972	-4.01164
12.05	0.07759415	0.1305889	-4.053211
12.06667	0.05371903	0.1305889	-4.080925
12.08333	0.05371903	0.1440972	-4.073997
12.1	0.05968781	0.1441017	-4.046283
12.11667	0.03581268	0.09005962	-4.025497
12.13333	0.05371903	0.1576055	-4.039354
12.15	0.01790634	0.1305889	-4.039354
12.16667	0.0298439	0.1260862	-4.039354
12.18333	0.08356293	0.1440972	-4.053211
12.2	0.0298439	0.1576055	-4.046283
12.21667	0.03581268	0.1260862	-4.039354
12.23333	0.04178147	0.1260862	-4.053211
12.25	0.04178147	0.1395945	-4.073997
12.26667	0.03581268	0.1395945	-4.080925
12.28333	0.0298439	0.1621082	-4.080925
12.3	0.04775025	0.1621127	-4.087854
12.31667	0.0298439	0.1395945	-4.094783
12.33333	0.0298439	0.1170807	-4.101711
12.35	0.0298439	0.1666155	-4.10864
12.36667	0.02387512	0.1621082	-4.080925
12.38333	0.0298439	0.1215834	-4.053211
12.4	0.03581268	0.1486	-4.039354
12.41667	0.0298439	0.1440972	-4.06014

12.43333	0.05371903	0.1395945	-4.080925
12.45	0.01790634	0.1260817	-4.080925
12.46667	0.07162537	0.1305889	-4.067069
12.48333	0.05371903	0.1621082	-4.067069
12.5	0.02387512	0.09906964	-4.087854
12.51667	0.04775025	0.1486	-4.094783
12.53333	0.03581268	0.09005962	-4.115569
12.55	0.03581268	0.1260817	-4.129426
12.56667	0.05968781	0.175621	-4.136354
12.58333	0.03581268	0.1350917	-4.136354
12.6	0.02387512	0.1621127	-4.129426
12.61667	0.03581268	0.1350917	-4.115569
12.63333	0.07162537	0.1711182	-4.101711
12.65	0.04178147	0.1170807	-4.115569
12.66667	0.04775025	0.1486	-4.129426
12.68333	0.05968781	0.1440972	-4.129426
12.7	0.07162537	0.1035679	-4.136354
12.71667	0.05371903	0.09906514	-4.143283
12.73333	0.04178147	0.1440972	-4.136354
12.75	0.05371903	0.1350917	-4.115569
12.76667	0.06565659	0.1260862	-4.094783
12.78333	0.06565659	0.1215834	-4.115569
12.8	0.03581268	0.1305889	-4.143283
12.81667	0.03581268	0.1260862	-4.150211
12.83333	0.02387512	0.1215789	-4.15714
12.85	0.0298439	0.1486	-4.150211
12.86667	0.06565659	0.1125779	-4.150211
12.88333	0.03581268	0.1576055	-4.15714
12.9	0.05371903	0.1666155	-4.164068
12.91667	0.08356293	0.1440972	-4.170997
12.93333	0.07162537	0.09906514	-4.164068
12.95	0.07162537	0.1486	-4.129426
12.96667	0.07759415	0.09005962	-4.122497
12.98333	0.06565659	0.1395945	-4.15714
13	0.07759415	0.1531027	-4.177926
13.01667	0.04178147	0.1350917	-4.170997
13.03333	0.04775025	0.1395945	-4.15714
13.05	0.06565659	0.1440972	-4.170997
13.06667	0.05371903	0.09005962	-4.20564
13.08333	0.02387512	0.1621082	-4.219497
13.1	0.02387512	0.1531072	-4.20564
13.11667	0.04178147	0.1440972	-4.191783
13.13333	0.03581268	0.1035724	-4.177926
13.15	0.04775025	0.1125734	-4.170997
13.16667	0.0298439	0.09906514	-4.164068
13.18333	0.04178147	0.1486	-4.170997
13.2	0.0298439	0.1215834	-4.184854

13.21667	0.04178147	0.1440972	-4.191783
13.23333	0.05968781	0.1260862	-4.170997
13.25	0.03581268	0.1170807	-4.143283
13.26667	0.01193756	0.1170762	-4.129426
13.28333	0.04178147	0.1395945	-4.129426
13.3	0.04775025	0.09456238	-4.129426
13.31667	0.02387512	0.1080706	-4.143283
13.33333	0.01193756	0.1125734	-4.177926
13.35	0.0298439	0.1621082	-4.20564
13.36667	0.05968781	0.1035724	-4.198711
13.38333	0.04178147	0.1891293	-4.20564
13.4	0.04178147	0.1350917	-4.212568
13.41667	0.04775025	0.1666155	-4.184854
13.43333	0.05968781	0.1395945	-4.164068
13.45	0.05371903	0.1080706	-4.191783
13.46667	0.04775025	0.04502756	-4.219497
13.48333	0.01790634	0.1530982	-4.212568
13.5	0.07162537	0.1170807	-4.212568
13.51667	0.0298439	0.1305844	-4.20564
13.53333	0.05371903	0.1125734	-4.198711
13.55	0.08356293	0.1711182	-4.20564
13.56667	0.07162537	0.1666155	-4.226426
13.58333	0.05371903	0.1305889	-4.219497
13.6	0.06565659	0.166611	-4.198711
13.61667	0.04775025	0.1080751	-4.198711
13.63333	0.05371903	0.1260817	-4.212568
13.65	0.04775025	0.1305889	-4.233354
13.66667	0.03581268	0.1395945	-4.25414
13.68333	0.04775025	0.175621	-4.25414
13.7	0.05371903	0.1080751	-4.25414
13.71667	0.04775025	0.1576055	-4.25414
13.73333	0.04775025	0.1486045	-4.240283
13.75	0.06565659	0.1395945	-4.233354
13.76667	0.04775025	0.1305889	-4.226426
13.78333	0.06565659	0.1170807	-4.226426
13.8	0.05371903	0.1125734	-4.240283
13.81667	0.04775025	0.1891248	-4.247211
13.83333	0.04775025	0.08555687	-4.25414
13.85	0.04775025	0.09456238	-4.247211
13.86667	0.06565659	0.1260817	-4.219497
13.88333	0.05968781	0.1215789	-4.212568
13.9	0.03581268	0.175621	-4.226426
13.91667	0.04178147	0.1756165	-4.247211
13.93333	0.04178147	0.1305889	-4.25414
13.95	0.02387512	0.1080706	-4.261068
13.96667	0.0298439	0.1486	-4.267997
13.98333	0.0298439	0.09456238	-4.281854

14	0.04178147	0.1350917	-4.281854
14.01667	0.06565659	0.1440972	-4.288783
14.03333	0.0298439	0.1035679	-4.295711
14.05	0.05371903	0.1440972	-4.295711
14.06667	0.05968781	0.1260862	-4.295711
14.08333	0.03581268	0.1215789	-4.295711
14.1	0.04775025	0.1711182	-4.288783
14.11667	0.04178147	0.1621082	-4.288783
14.13333	0.04178147	0.1260862	-4.295711
14.15	0.03581268	0.1711137	-4.295711
14.16667	0.1014693	0.1170807	-4.288783
14.18333	0.04178147	0.1170807	-4.295711
14.2	0.03581268	0.1621127	-4.316497
14.21667	0.0298439	0.1170807	-4.330354
14.23333	0.0298439	0.1215834	-4.344211
14.25	0.05968781	0.1080706	-4.358068
14.26667	0.05968781	0.1350872	-4.371925
14.28333	0.09550049	0.1440972	-4.371925
14.3	0.03581268	0.1035679	-4.358068
14.31667	0.05968781	0.1170762	-4.337283
14.33333	0.04775025	0.1486	-4.344211
14.35	0.03581268	0.1305889	-4.35114
14.36667	0.06565659	0.1305889	-4.371925
14.38333	0.03581268	0.1305889	-4.385783
14.4	0.04775025	0.1395945	-4.39964
14.41667	0.005968781	0.1260862	-4.406568
14.43333	0.05371903	0.1215834	-4.39964
14.45	0.06565659	0.1035679	-4.39964
14.46667	0.05371903	0.1305889	-4.413497
14.48333	0.04178147	0.2026375	-4.406568
14.5	0.04775025	0.15761	-4.385783
14.51667	0.05968781	0.1666155	-4.364997
14.53333	0.05371903	0.1260862	-4.371925
14.55	0.03581268	0.1125734	-4.39964
14.56667	0.06565659	0.1215789	-4.413497
14.58333	0.04775025	0.1440972	-4.406568
14.6	0.02387512	0.1531027	-4.413497
14.61667	0.03581268	0.1486	-4.427354
14.63333	0.03581268	0.1395945	-4.434283
14.65	0.06565659	0.1260862	-4.427354
14.66667	0.0298439	0.1305889	-4.39964
14.68333	0.04775025	0.1486	-4.385783
14.7	0.04775025	0.1350917	-4.378854
14.71667	0.01193756	0.1260862	-4.39964
14.73333	0.0298439	0.1531027	-4.420425
14.75	0.04178147	0.1395945	-4.441211
14.76667	0.01790634	0.1395945	-4.44814

14.78333	0.09550049	0.1486	-4.434283
14.8	0.04775025	0.1621127	-4.420425
14.81667	0.0298439	0.1711182	-4.420425
14.83333	0.01193756	0.1395945	-4.420425
14.85	0.07162537	0.1440972	-4.441211
14.86667	0.04775025	0.1260862	-4.461997
14.88333	0.0298439	0.1035724	-4.468925
14.9	0.01790634	0.1440927	-4.468925
14.91667	0.04178147	0.1260862	-4.455068
14.93333	0.04775025	0.1305889	-4.441211
14.95	0.05371903	0.1395945	-4.44814
14.96667	0.05371903	0.1080706	-4.475854
14.98333	0.02387512	0.1260862	-4.503568
15	0.02387512	0.1260862	-4.510497
15.01667	0.01790634	0.09456238	-4.510497
15.03333	0.0298439	0.1350917	-4.517426
15.05	0.04775025	0.1080706	-4.503568
15.06667	0.0298439	0.1035679	-4.482782
15.08333	0.02387512	0.1035679	-4.489711
15.1	0.07162537	0.1215834	-4.503568
15.11667	0.0298439	0.1486	-4.503568
15.13333	0.01790634	0.1170762	-4.489711
15.15	0.05371903	0.1260817	-4.455068
15.16667	0.04178147	0.1305889	-4.455068
15.18333	0.0298439	0.1035679	-4.475854
15.2	0.05968781	0.1305844	-4.489711
15.21667	0.03581268	0.08555687	-4.503568
15.23333	0.04775025	0.1531027	-4.503568
15.25	0.05371903	0.1260862	-4.49664
15.26667	0.05371903	0.1350917	-4.49664
15.28333	0.0298439	0.1440972	-4.489711
15.3	0.05371903	0.1035679	-4.489711
15.31667	0.05371903	0.1486	-4.503568
15.33333	0.05371903	0.15761	-4.524354
15.35	0.06565659	0.1305889	-4.54514
15.36667	0.04178147	0.1395945	-4.558997
15.38333	0.0298439	0.1260862	-4.538211
15.4	0.05968781	0.1711137	-4.510497
15.41667	0.04178147	0.1125779	-4.503568
15.43333	0.01193756	0.08555687	-4.503568
15.45	0.04775025	0.1170762	-4.517426
15.46667	0.03581268	0.1891293	-4.538211
15.48333	0.05371903	0.1170807	-4.538211
15.5	0.03581268	0.1305889	-4.510497
15.51667	0.0298439	0.1215834	-4.482782
15.53333	0.04775025	0.1846265	-4.489711
15.55	0.03581268	0.09456688	-4.49664

15.56667	0.02387512	0.1350917	-4.510497
15.58333	0.05371903	0.1080706	-4.524354
15.6	0.04178147	0.09906514	-4.531282
15.61667	0.04775025	0.1260817	-4.538211
15.63333	0.03581268	0.1711182	-4.524354
15.65	0.03581268	0.1305889	-4.503568
15.66667	0.0298439	0.1080706	-4.482782
15.68333	0.02387512	0.1260817	-4.475854
15.7	0.05968781	0.1531027	-4.503568
15.71667	0.05968781	0.1395945	-4.517426
15.73333	0.02387512	0.1711182	-4.503568
15.75	0.06565659	0.1350917	-4.49664
15.76667	0.04178147	0.1621127	-4.503568
15.78333	0.07759415	0.1260862	-4.510497
15.8	0.02387512	0.1260862	-4.503568
15.81667	0.0298439	0.1531027	-4.49664
15.83333	0.03581268	0.1260862	-4.49664
15.85	0.0298439	0.1531027	-4.49664
15.86667	0.04178147	0.09456238	-4.482782
15.88333	0.07759415	0.1395945	-4.489711
15.9	0.04775025	0.1486	-4.49664
15.91667	0.03581268	0.1305889	-4.49664
15.93333	0.02387512	0.1395945	-4.482782
15.95	0.02387512	0.1125734	-4.475854
15.96667	0.02387512	0.08105411	-4.468925
15.98333	0.04178147	0.1260817	-4.475854
16	0.03581268	0.1531072	-4.49664
16.01667	0.0298439	0.1215834	-4.517426
16.03333	0.04178147	0.1891293	-4.538211
16.05	0.05968781	0.09005962	-4.531282
16.06667	0.03581268	0.1350872	-4.524354
16.08333	0.05968781	0.1215834	-4.524354
16.1	0.02387512	0.1350917	-4.517426
16.11667	0.05371903	0.1531027	-4.503568
16.13333	0.04178147	0.1080706	-4.503568
16.15	0.0298439	0.1170807	-4.49664
16.16667	0.05371903	0.1305844	-4.489711
16.18333	0.04775025	0.1666155	-4.503568
16.2	0.04775025	0.1531072	-4.510497
16.21667	0.07162537	0.1125779	-4.489711
16.23333	0.0298439	0.1711182	-4.489711
16.25	0.03581268	0.1305889	-4.503568
16.26667	0.05371903	0.1440972	-4.503568
16.28333	0.03581268	0.1486	-4.510497
16.3	0.05371903	0.1170807	-4.524354
16.31667	0.04775025	0.1486	-4.531282
16.33333	0.05371903	0.1260862	-4.552068

16.35	0.005968781	0.1576055	-4.54514
16.36667	0.04775025	0.1215834	-4.531282
16.38333	0.02387512	0.1305889	-4.524354
16.4	0.05968781	0.15761	-4.531282
16.41667	0.05371903	0.1395945	-4.524354
16.43333	0.0298439	0.1305889	-4.524354
16.45	0.04775025	0.08555687	-4.531282
16.46667	0.04178147	0.1531027	-4.531282
16.48333	0.04775025	0.1125779	-4.531282
16.5	0.04178147	0.166611	-4.538211
16.51667	0.0298439	0.1441017	-4.531282
16.53333	0.05371903	0.1125734	-4.503568
16.55	0.04178147	0.1260862	-4.489711
16.56667	0.04178147	0.1395945	-4.482782
16.58333	0.03581268	0.1305889	-4.489711
16.6	0.05968781	0.1305889	-4.49664
16.61667	0.04178147	0.1215834	-4.503568
16.63333	0.04178147	0.1440972	-4.517426
16.65	0.05968781	0.1711182	-4.503568
16.66667	0.06565659	0.1531027	-4.49664
16.68333	0.03581268	0.1441017	-4.489711
16.7	0.05371903	0.15761	-4.482782
16.71667	0.04775025	0.1350917	-4.475854
16.73333	0.04178147	0.09906514	-4.468925
16.75	0.02387512	0.08105411	-4.475854
16.76667	0.04178147	0.1440972	-4.489711
16.78333	0.03581268	0.1395945	-4.503568
16.8	0.0298439	0.09005962	-4.503568
16.81667	0.08356293	0.1350872	-4.489711
16.83333	0.03581268	0.166611	-4.468925
16.85	0.01790634	0.1350962	-4.461997
16.86667	0.05968781	0.1486	-4.489711
16.88333	0.05968781	0.1170807	-4.517426
16.9	0.04775025	0.1305844	-4.517426
16.91667	0.03581268	0.1350917	-4.524354
16.93333	0.05968781	0.1305889	-4.503568
16.95	0.04775025	0.1035679	-4.49664
16.96667	0.03581268	0.1486	-4.482782
16.98333	0.05968781	0.1531027	-4.455068
17	0.05968781	0.15761	-4.44814
17.01667	0.03581268	0.1260862	-4.461997
17.03333	0.04178147	0.09906514	-4.468925
17.05	0.02387512	0.1440972	-4.461997
17.06667	0.04178147	0.1260862	-4.455068
17.08333	0.05371903	0.1305889	-4.475854
17.1	0.05371903	0.1531027	-4.489711
17.11667	0.01790634	0.1215834	-4.468925

17.13333	0.005968781	0.1531027	-4.455068
17.15	0.03581268	0.1125734	-4.455068
17.16667	0.04775025	0.13959	-4.468925
17.18333	0.0298439	0.1260862	-4.475854
17.2	0.04178147	0.1080706	-4.461997
17.21667	0.05371903	0.1395945	-4.461997
17.23333	0.0298439	0.09005962	-4.468925
17.25	0.05371903	0.1305844	-4.455068
17.26667	0.06565659	0.1621127	-4.441211
17.28333	0.05371903	0.1395945	-4.441211
17.3	0.05968781	0.1125734	-4.455068
17.31667	0.08356293	0.1486	-4.461997
17.33333	0.04178147	0.1125734	-4.461997
17.35	0.0298439	0.1350917	-4.468925
17.36667	0.04178147	0.1621082	-4.455068
17.38333	0.05371903	0.1035679	-4.427354
17.4	0.03581268	0.1395945	-4.420425
17.41667	0.05371903	0.1711182	-4.427354
17.43333	0.04775025	0.1215834	-4.441211
17.45	0.04775025	0.1305889	-4.420425
17.46667	0.06565659	0.1305889	-4.392711
17.48333	0.05968781	0.1440972	-4.39964
17.5	0.04775025	0.1440972	-4.420425
17.51667	0.0298439	0.1486	-4.427354
17.53333	0.02387512	0.1215834	-4.434283
17.55	0.04775025	0.1260862	-4.468925
17.56667	0.0298439	0.1125734	-4.475854
17.58333	0.01790634	0.1170762	-4.461997
17.6	0.0298439	0.1215789	-4.461997
17.61667	0.07162537	0.1035679	-4.434283
17.63333	0.04178147	0.1035679	-4.39964
17.65	0.04775025	0.1260862	-4.39964
17.66667	0.0298439	0.1215789	-4.413497
17.68333	0.04178147	0.166611	-4.427354
17.7	0.01790634	0.1125779	-4.441211
17.71667	0.06565659	0.1350917	-4.455068
17.73333	0.07759415	0.1305889	-4.455068
17.75	0.01193756	0.1350917	-4.44814
17.76667	0.05371903	0.1486	-4.455068
17.78333	0.05968781	0.1260862	-4.441211
17.8	0.03581268	0.1350917	-4.427354
17.81667	0.06565659	0.1666155	-4.427354
17.83333	0.04775025	0.1440972	-4.427354
17.85	0.02387512	0.1621082	-4.441211
17.86667	0.07759415	0.1080751	-4.441211
17.88333	0.04178147	0.1576055	-4.434283
17.9	0.05968781	0.1350917	-4.427354

17.91667	0.05371903	0.1170762	-4.420425
17.93333	0.05371903	0.1035679	-4.413497
17.95	0.02387512	0.1305889	-4.427354
17.96667	0.05968781	0.1035679	-4.461997
17.98333	0.02387512	0.1801192	-4.468925
18	0.0298439	0.1486045	-4.461997
18.01667	0.03581268	0.1125734	-4.475854
18.03333	0.05968781	0.1215789	-4.475854
18.05	0.03581268	0.1305889	-4.461997
18.06667	0.02387512	0.1170807	-4.461997
18.08333	0.005968781	0.1440972	-4.455068
18.1	0.02387512	0.1486045	-4.44814
18.11667	0.05968781	0.1215834	-4.427354
18.13333	0.0298439	0.1080706	-4.413497
18.15	0.0298439	0.1125734	-4.413497
18.16667	0.05968781	0.1395945	-4.420425
18.18333	0.03581268	0.1666155	-4.420425
18.2	0.06565659	0.1666155	-4.427354
18.21667	0.05371903	0.1395945	-4.434283
18.23333	0.04775025	0.1350917	-4.434283
18.25	0.05968781	0.09456238	-4.420425
18.26667	0.01790634	0.166611	-4.420425
18.28333	0.07162537	0.08105411	-4.434283
18.3	0.0298439	0.1485955	-4.434283
18.31667	0.04775025	0.1170807	-4.427354
18.33333	0.08953171	0.1260817	-4.427354
18.35	0.04775025	0.1486	-4.413497
18.36667	0.03581268	0.15761	-4.385783
18.38333	0.04178147	0.1395945	-4.39964
18.4	0.05968781	0.15761	-4.427354
18.41667	0.05371903	0.1215834	-4.427354
18.43333	0.03581268	0.1350917	-4.434283
18.45	0.05371903	0.1801238	-4.441211
18.46667	0.06565659	0.15761	-4.44814
18.48333	0.01790634	0.1080751	-4.475854
18.5	0.02387512	0.1305889	-4.468925
18.51667	0.05968781	0.1170807	-4.455068
18.53333	0.01790634	0.1395945	-4.455068
18.55	0.0298439	0.09456238	-4.44814
18.56667	0.05371903	0.1260817	-4.455068
18.58333	0.0298439	0.1440972	-4.455068
18.6	0.04178147	0.1215834	-4.461997
18.61667	0.04775025	0.1170807	-4.482782
18.63333	0.04775025	0.1215789	-4.475854
18.65	0.05371903	0.1576055	-4.455068
18.66667	0.02387512	0.1936365	-4.441211
18.68333	0.07162537	0.1711182	-4.441211

18.7	0.05371903	0.1395945	-4.427354
18.71667	0.04775025	0.1350917	-4.413497
18.73333	0.05371903	0.1440972	-4.413497
18.75	0.04178147	0.1305889	-4.434283
18.76667	0.07759415	0.1170762	-4.44814
18.78333	0.02387512	0.1125779	-4.461997
18.8	0.03581268	0.166611	-4.475854
18.81667	0.02387512	0.1531027	-4.482782
18.83333	0.04178147	0.1395945	-4.475854
18.85	0.0298439	0.09456238	-4.475854
18.86667	0.04178147	0.1576055	-4.49664
18.88333	0.02387512	0.1170807	-4.482782
18.9	0.06565659	0.1170762	-4.475854
18.91667	0.05371903	0.1170762	-4.468925
18.93333	0.02387512	0.1170762	-4.461997
18.95	0.03581268	0.1621127	-4.455068
18.96667	0.0298439	0.1395945	-4.461997
18.98333	0.005968781	0.1170807	-4.468925
19	0.05371903	0.1260862	-4.468925
19.01667	0.02387512	0.1440972	-4.461997
19.03333	0.08356293	0.1035679	-4.475854
19.05	0.02387512	0.08105411	-4.49664
19.06667	0.05968781	0.1035679	-4.524354
19.08333	0.04178147	0.1350917	-4.531282
19.1	0.04178147	0.1756165	-4.517426
19.11667	0.05968781	0.1305889	-4.531282
19.13333	0.08356293	0.1711182	-4.565925
19.15	0.07162537	0.1260862	-4.572854
19.16667	0.04775025	0.1621082	-4.552068
19.18333	0.07759415	0.1350917	-4.54514
19.2	0.02387512	0.1035679	-4.558997
19.21667	0.02387512	0.1981348	-4.565925
19.23333	0.02387512	0.1531072	-4.565925
19.25	0.05968781	0.1801238	-4.558997
19.26667	0.04178147	0.1395945	-4.54514
19.28333	0.08356293	0.09456238	-4.531282
19.3	0.04775025	0.1305844	-4.538211
19.31667	0.0298439	0.1440972	-4.54514
19.33333	0.02387512	0.1080751	-4.531282
19.35	0.0298439	0.1441017	-4.524354
19.36667	0.05371903	0.1395945	-4.538211
19.38333	0.05968781	0.09906514	-4.565925
19.4	0.04775025	0.1305889	-4.572854
19.41667	0.04775025	0.1305844	-4.565925
19.43333	0.03581268	0.1486	-4.572854
19.45	0.07162537	0.1711182	-4.572854
19.46667	0.08953171	0.1486045	-4.579782

19.48333	0.07759415	0.1080706	-4.579782
19.5	0.06565659	0.1486045	-4.586711
19.51667	0.05371903	0.1486	-4.614425
19.53333	0.04775025	0.1260862	-4.621354
19.55	0.05968781	0.1215834	-4.600568
19.56667	0.0298439	0.1576055	-4.593639
19.58333	0.05968781	0.1666155	-4.607497
19.6	0.05371903	0.1666155	-4.621354
19.61667	0.07162537	0.1531027	-4.635211
19.63333	0.02387512	0.1801238	-4.635211
19.65	0.08356293	0.1621127	-4.635211
19.66667	0.02387512	0.1215834	-4.655997
19.68333	0.07162537	0.1395945	-4.676783
19.7	0.07162537	0.1891293	-4.649068
19.71667	0.06565659	0.1440972	-4.635211
19.73333	0.02387512	0.1711137	-4.649068
19.75	0.05371903	0.1215834	-4.662925
19.76667	0.01790634	0.1486	-4.649068
19.78333	0.03581268	0.1170762	-4.642139
19.8	0.03581268	0.1260862	-4.655997
19.81667	0.06565659	0.1350917	-4.676783
19.83333	0.05371903	0.1531027	-4.697568
19.85	0.07759415	0.15761	-4.704497
19.86667	0.07759415	0.1801238	-4.704497
19.88333	0.07759415	0.1531072	-4.711425
19.9	0.07162537	0.09906514	-4.73914
19.91667	0.07162537	0.1395945	-4.759925
19.93333	0.03581268	0.1170762	-4.752996
19.95	0.07162537	0.1440972	-4.725282
19.96667	0.08356293	0.1576055	-4.725282
19.98333	0.05968781	0.1666155	-4.766854
20	0.03581268	0.15761	-4.78764
20.01667	0.05371903	0.1621127	-4.808425
20.03333	0.03581268	0.1531027	-4.836139
20.05	0.05371903	0.1170807	-4.843068
20.06667	0.04775025	0.1576055	-4.836139
20.08333	0.0298439	0.1170807	-4.843068
20.1	0.08953171	0.1305889	-4.863853
20.11667	0.0298439	0.1305889	-4.870782
20.13333	0.05968781	0.1395945	-4.849997
20.15	0.03581268	0.1260862	-4.829211
20.16667	0.05371903	0.1395945	-4.843068
20.18333	0.08953171	0.1846265	-4.884639
20.2	0.04178147	0.1350962	-4.905425
20.21667	0.07162537	0.1395945	-4.912354
20.23333	0.06565659	0.1035679	-4.905425
20.25	0.04775025	0.1711137	-4.898497

20.26667	0.04178147	0.1531072	-4.898497
20.28333	0.0298439	0.15761	-4.912354
20.3	0.05371903	0.1440972	-4.933139
20.31667	0.04178147	0.15761	-4.946997
20.33333	0.05371903	0.1350917	-4.953925
20.35	0.05371903	0.166611	-4.953925
20.36667	0.06565659	0.1486045	-4.960854
20.38333	0.0298439	0.1170807	-4.988568
20.4	0.05968781	0.166611	-5.016282
20.41667	0.01193756	0.1441017	-5.030139
20.43333	0.07162537	0.1756165	-5.037068
20.45	0.04775025	0.1440972	-5.030139
20.46667	0.03581268	0.1486	-5.023211
20.48333	0.02387512	0.1486045	-5.030139
20.5	0.04775025	0.1621082	-5.050925
20.51667	0.04178147	0.1531072	-5.050925
20.53333	0.07162537	0.1215834	-5.043996
20.55	0.03581268	0.1305844	-5.043996
20.56667	0.07759415	0.1170807	-5.071711
20.58333	0.03581268	0.166611	-5.106353
20.6	0.01790634	0.1666155	-5.113282
20.61667	0.03581268	0.1486	-5.113282
20.63333	0.04775025	0.175621	-5.113282
20.65	0.0298439	0.1440972	-5.099425
20.66667	0.01790634	0.184631	-5.106353
20.68333	0.04775025	0.1486	-5.140996
20.7	0.0298439	0.1350917	-5.175639
20.71667	0.03581268	0.1305889	-5.189497
20.73333	0.08356293	0.1170807	-5.217211
20.75	0.04775025	0.1035679	-5.237996
20.76667	0.08356293	0.1711137	-5.224139
20.78333	0.02387512	0.1170807	-5.196425
20.8	0.07162537	0.1260817	-5.182568
20.81667	0.0298439	0.09456238	-5.196425
20.83333	0.05371903	0.1711137	-5.217211
20.85	0.04178147	0.1441017	-5.224139
20.86667	0.05371903	0.1350917	-5.224139
20.88333	0.02387512	0.1711182	-5.244925
20.9	0.0298439	0.1531072	-5.272639
20.91667	0.08356293	0.1486	-5.293425
20.93333	0.07162537	0.1846265	-5.300354
20.95	0.04178147	0.1666155	-5.300354
20.96667	0.04178147	0.1441017	-5.300354
20.98333	0.07162537	0.1891293	-5.321139
21	0.05371903	0.1981348	-5.341925
21.01667	0.0298439	0.1441017	-5.348853
21.03333	0.05968781	0.15761	-5.355782

21.05	0.05371903	0.1576055	-5.36271
21.06667	0.0298439	0.1350917	-5.376567
21.08333	0.05371903	0.15761	-5.369639
21.1	0.04775025	0.193632	-5.369639
21.11667	0.07162537	0.1260862	-5.397353
21.13333	0.04178147	0.1395945	-5.431996
21.15	0.06565659	0.1486	-5.445853
21.16667	0.03581268	0.1350917	-5.459711
21.18333	0.07759415	0.1395945	-5.473567
21.2	0.07759415	0.1170762	-5.487425
21.21667	0.06565659	0.1305889	-5.515139
21.23333	0.05371903	0.1891293	-5.535924
21.25	0.04775025	0.09906514	-5.549782
21.26667	0.05371903	0.1215789	-5.570568
21.28333	0.04775025	0.1576055	-5.584424
21.3	0.05371903	0.1170807	-5.60521
21.31667	0.01193756	0.1170762	-5.60521
21.33333	0.07162537	0.184622	-5.612139
21.35	0.05968781	0.15761	-5.639853
21.36667	0.01193756	0.1531027	-5.674496
21.38333	0.05968781	0.1486	-5.660639
21.4	0.05968781	0.175621	-5.646781
21.41667	0.05371903	0.175621	-5.674496
21.43333	0.04178147	0.1305889	-5.681425
21.45	0.07162537	0.1531027	-5.688353
21.46667	0.01790634	0.1395945	-5.722996
21.48333	0.03581268	0.166611	-5.75071
21.5	0.03581268	0.2026375	-5.757639
21.51667	0.0298439	0.1350962	-5.757639
21.53333	0.06565659	0.1080706	-5.771496
21.55	0.04775025	0.1395945	-5.792282
21.56667	0.05371903	0.1486	-5.79921
21.58333	0.03581268	0.1395945	-5.806139
21.6	0.03581268	0.1305889	-5.826924
21.61667	0.05371903	0.1440972	-5.84771
21.63333	0.05968781	0.1576055	-5.861567
21.65	0.04775025	0.1621127	-5.868496
21.66667	0.05968781	0.1891293	-5.875424
21.68333	0.03581268	0.15761	-5.875424
21.7	0.03581268	0.1170807	-5.889281
21.71667	0.05968781	0.1486	-5.903139
21.73333	0.05968781	0.1350917	-5.916996
21.75	0.05968781	0.1756165	-5.965496
21.76667	0.04178147	0.1260862	-6.027853
21.78333	0.005968781	0.1801192	-6.062496
21.8	0.04775025	0.1350917	-6.069424
21.81667	0.04178147	0.1576055	-6.076353

21.83333	0.04775025	0.1125779	-6.09021
21.85	0.04775025	0.1486	-6.117924
21.86667	0.02387512	0.1711137	-6.13871
21.88333	0.06565659	0.1531072	-6.152567
21.9	0.05371903	0.1305889	-6.18721
21.91667	0.04775025	0.1621082	-6.207995
21.93333	0.05371903	0.1531072	-6.214924
21.95	0.01193756	0.1260862	-6.207995
21.96667	0.06565659	0.1305889	-6.207995
21.98333	0.05371903	0.1350917	-6.23571
22	0.01790634	0.09456238	-6.263424
22.01667	0.06565659	0.211643	-6.270353
22.03333	0.04178147	0.1305889	-6.28421
22.05	0.01790634	0.1215834	-6.318852
22.06667	0.06565659	0.1440972	-6.346567
22.08333	0.02387512	0.1260862	-6.346567
22.1	0.0298439	0.1440972	-6.346567
22.11667	0.03581268	0.1621082	-6.367352
22.13333	0.04775025	0.1125734	-6.395067
22.15	0.02387512	0.1486	-6.415853
22.16667	0.04775025	0.1215834	-6.436638
22.18333	0.04775025	0.1756165	-6.464353
22.2	0.06565659	0.1215834	-6.492067
22.21667	0.03581268	0.1260862	-6.505924
22.23333	0.0298439	0.1801238	-6.52671
22.25	0.03581268	0.1486	-6.554424
22.26667	0.01790634	0.1305889	-6.57521
22.28333	0.0298439	0.09005962	-6.582138
22.3	0.05371903	0.1395945	-6.589067
22.31667	0.04178147	0.1080751	-6.616781
22.33333	0.03581268	0.1801192	-6.651424
22.35	0.08356293	0.15761	-6.67221
22.36667	0.07162537	0.1666155	-6.679138
22.38333	0.01790634	0.1486	-6.686067
22.4	0.02387512	0.1080751	-6.699924
22.41667	0.03581268	0.1260862	-6.720709
22.43333	0.06565659	0.1350917	-6.741495
22.45	0.04178147	0.1350917	-6.769209
22.46667	0.05371903	0.1350917	-6.783067
22.48333	0.01790634	0.1350917	-6.776138
22.5	0.06565659	0.1305889	-6.762281
22.51667	0.05371903	0.1170762	-6.762281
22.53333	0.03581268	0.1711137	-6.796924
22.55	0.05968781	0.1305889	-6.838495
22.56667	0.04178147	0.09906514	-6.86621
22.58333	0.05968781	0.1486	-6.86621
22.6	0.04178147	0.1395945	-6.852352

22.61667	0.03581268	0.1440972	-6.852352
22.63333	0.05968781	0.1576055	-6.880066
22.65	0.01790634	0.1711182	-6.900852
22.66667	0.04775025	0.1441017	-6.907781
22.68333	0.05968781	0.1666155	-6.893924
22.7	0.07162537	0.1260862	-6.900852
22.71667	0.04775025	0.09906514	-6.921638
22.73333	0.0298439	0.1260817	-6.928566
22.75	0.04775025	0.09456238	-6.928566
22.76667	0.0298439	0.1125734	-6.935495
22.78333	0.03581268	0.1621082	-6.942424
22.8	0.0298439	0.1711182	-6.949352
22.81667	0.04775025	0.1260862	-6.949352
22.83333	0.03581268	0.09906514	-6.935495
22.85	0.05968781	0.09456238	-6.928566
22.86667	0.02387512	0.09005962	-6.942424
22.88333	0.02387512	0.09456238	-6.942424
22.9	0.04178147	0.1531027	-6.935495
22.91667	0.03581268	0.1486045	-6.921638
22.93333	0.04178147	0.1260862	-6.880066
22.95	0.02387512	0.1080706	-6.831566
22.96667	0.03581268	0.1260862	-6.810781
22.98333	0.06565659	0.1305889	-6.796924
23	0.05968781	0.09005962	-6.776138
23.01667	0.0298439	0.1531027	-6.741495
23.03333	0.04178147	0.08555687	-6.706852
23.05	0.05371903	0.13959	-6.658352
23.06667	0.05968781	0.15761	-6.602924
23.08333	0.04775025	0.1125734	-6.547495
23.1	0.01790634	0.1486	-6.492067
23.11667	0.04178147	0.1711182	-6.408924
23.13333	0.05371903	0.1035724	-6.304996
23.15	0.04775025	0.1305889	-6.18721
23.16667	0.0298439	0.1440972	-6.048638
23.18333	0.04178147	0.1260862	-5.910067
23.2	0.08356293	0.1260862	-5.771496
23.21667	0.07162537	0.1801238	-5.60521
23.23333	0.07162537	0.1395945	-5.431996
23.25	0.05371903	0.1486	-5.244925
23.26667	0.04178147	0.1305889	-5.030139
23.28333	0.05968781	0.07655136	-4.801497
23.3	0.03581268	0.09456238	-4.54514
23.31667	0.0298439	0.1350917	-4.274926
23.33333	0.04178147	0.1080706	-4.004712
23.35	0.03581268	0.09005962	-3.727569
23.36667	0.05371903	0.1486	-3.415783
23.38333	0.04775025	0.1666155	-3.069355

23.4	0.04178147	0.1486045	-2.688284
23.41667	0.08356293	0.1305889	-2.286427
23.43333	0.05371903	0.1305889	-1.870713
23.45	0.06565659	0.09906514	-1.427285
23.46667	0.07759415	0.1576055	-0.9561422
23.48333	0.08356293	0.1486	-0.464214
23.5	0.05371903	0.1711182	0.04849996
23.51667	0.05968781	0.1350917	0.6027853
23.53333	0.05371903	0.193632	1.191713
23.55	0.05968781	0.1395945	1.815284
23.56667	0.04775025	0.1531027	2.487355
23.58333	0.05371903	0.1080751	3.180212
23.6	0.07162537	0.1711182	3.900783
23.61667	0.08356293	0.1260862	4.649068
23.63333	0.06565659	0.1305844	5.425067
23.65	0.04775025	0.09456238	6.228781
23.66667	0.08953171	0.1260817	7.074066
23.68333	0.1014752	0.1395945	7.947066
23.7	0.05968781	0.1170762	8.854708
23.71667	0.107444	0.1440972	9.783135
23.73333	0.07759415	0.1350917	10.72542
23.75	0.1372879	0.1170807	11.71621
23.76667	0.07163134	0.1350917	12.77628
23.78333	0.1551943	0.1486	13.87792
23.8	0.1134128	0.1621127	14.98649
23.81667	0.1850382	0.1215834	16.12277
23.83333	0.1372939	0.1215834	17.31449
23.85	0.1790754	0.1305889	18.54084
23.86667	0.1372879	0.1621082	19.80184
23.88333	0.1372879	0.1531027	21.09748
23.9	0.1850382	0.1170807	22.42084
23.91667	0.268619	0.1215834	23.77191
23.93333	0.2089252	0.1486	25.15763
23.95	0.1850441	0.1260862	26.56412
23.96667	0.3402444	0.1035679	27.99834
23.98333	0.3641374	0.1711137	29.48105
24	0.2566874	0.15761	30.99841
24.01667	0.3342816	0.1305889	32.52962
24.03333	0.2925001	0.1350917	34.06083
24.05	0.4596499	0.1486	35.63362
24.06667	0.3342995	0.1801238	37.24104
24.08333	0.4477063	0.1260862	38.8554
24.1	0.5372679	0.1531027	40.49747
24.11667	0.5552101	0.1440972	42.16033
24.13333	0.5432605	0.1621082	43.83011
24.15	0.6029543	0.09906964	45.51375
24.16667	0.5193914	0.13959	47.21818

24.18333	0.8357905	0.1395945	48.95032
24.2	0.7104878	0.1125734	50.70325
24.21667	0.7403079	0.1125734	52.4631
24.23333	0.9134503	0.07655136	54.21603
24.25	0.9911399	0.1350872	55.96896
24.26667	0.961302	0.1080751	57.72189
24.28333	1.09862	0.1125734	59.48867
24.3	1.212116	0.1395945	61.26239
24.31667	1.104672	0.1350917	62.98067
24.33333	1.295751	0.1170807	64.65738
24.35	1.355492	0.1531027	66.35488
24.36667	1.355546	0.1260862	68.08009
24.38333	1.660085	0.1395945	69.79838
24.4	1.666275	0.1170762	71.48895
24.41667	1.827462	0.1576055	73.12409
24.43333	1.923153	0.1305889	74.69688
24.45	1.94707	0.1215834	76.24887
24.46667	1.965012	0.1125734	77.80087
24.48333	2.22192	0.1440972	79.33901
24.5	2.263726	0.1170807	80.80787
24.51667	2.407251	0.1260817	82.24902
24.53333	2.724032	0.1350917	83.66244
24.55	2.802008	0.1531027	84.99966
24.56667	3.088784	0.1170807	86.27451
24.58333	3.095016	0.1260862	87.48701
24.6	3.417855	0.1621127	88.67179
24.61667	3.537529	0.1170762	89.82887
24.63333	3.758905	0.1125734	90.9305
24.65	4.052062	0.1531027	91.98365
24.66667	4.130008	0.15761	92.96751
24.68333	4.596558	0.1440972	93.88901
24.7	4.47159	0.1260862	94.72736
24.71667	5.14103	0.1621082	95.48257
24.73333	5.3274	0.1215834	96.1685
24.75	5.537268	0.1395945	96.81979
24.76667	5.369963	0.1395945	97.40871
24.78333	5.979733	0.1711182	97.92143
24.8	6.070382	0.1441017	98.37179
24.81667	6.453816	0.1170762	98.77364
24.83333	6.927158	0.1170762	99.14086
24.85	7.203095	0.1531027	99.45264
24.86667	7.760585	0.1486045	99.68822
24.88333	8.060905	0.1035679	99.8545
24.9	8.58861	0.166611	99.9515
24.91667	8.386716	0.15761	100
24.93333	9.044296	0.1170807	99.98615
24.95	9.783847	0.1260817	99.91685

24.96667	9.820853	0.1080751	99.79214
24.98333	9.767194	0.1576055	99.60507
25	11.00115	0.1080751	99.37643
25.01667	11.67673	0.1215789	99.12007
25.03333	12.07991	0.1305889	98.82214
25.05	12.20284	0.175621	98.46186
25.06667	12.3102	0.1260862	98.03229
25.08333	12.95237	0.1576055	97.55421
25.1	13.80638	0.1350917	97.04843
25.11667	14.31398	0.1440972	96.50108
25.13333	14.83306	0.09005962	95.89136
25.15	15.05177	0.1395945	95.22622
25.16667	15.98805	0.1395945	94.568
25.18333	16.67589	0.1080706	93.87514
25.2	17.23294	0.1170762	93.10608
25.21667	17.9686	0.1170762	92.30929
25.23333	18.87475	0.1170762	91.464
25.25	18.90143	0.1215834	90.57022
25.26667	19.87916	0.1531027	89.62794
25.28333	20.59303	0.1170807	88.67179
25.3	21.21495	0.1215789	87.71565
25.31667	22.41182	0.1440972	86.71101
25.33333	23.07649	0.09906514	85.67172
25.35	23.68464	0.1981348	84.60473
25.36667	24.4172	0.1260862	83.48923
25.38333	25.28675	0.1260862	82.32523
25.4	26.43142	0.1395945	81.13351
25.41667	26.50326	0.1305889	79.93487
25.43333	26.90713	0.1440972	78.7293
25.45	28.94812	0.1215834	77.48909
25.46667	29.32905	0.1936275	76.21423
25.48333	30.90765	0.1350962	74.91859
25.5	30.95483	0.1125779	73.60909
25.51667	31.20845	0.1125734	72.27187
25.53333	32.62885	0.1305889	70.91388
25.55	33.38459	0.1305889	69.5351
25.56667	34.15405	0.1891293	68.14938
25.58333	35.19537	0.1801283	66.74981
25.6	36.44686	0.1350917	65.35717
25.61667	37.46228	0.1215789	63.94374
25.63333	39.56862	0.1215834	62.51646
25.65	39.52002	0.1170807	61.06839
25.66667	40.37741	0.1350917	59.62032
25.68333	41.35851	0.1260817	58.17917
25.7	42.74818	0.1260862	56.72417
25.71667	43.59833	0.1711137	55.26918
25.73333	45.42294	0.1305889	53.83496

25.75	45.72023	0.1125779	52.40075
25.76667	45.96731	0.1170762	50.95268
25.78333	48.17092	0.1350917	49.52539
25.8	48.59759	0.1260862	48.14661
25.81667	50.80425	0.1486	46.77475
25.83333	50.44745	0.1260862	45.38211
25.85	52.30331	0.1621082	43.98254
25.86667	53.91201	0.1080751	42.62454
25.88333	55.079	0.1350917	41.27347
25.9	54.92299	0.09906514	39.93626
25.91667	57.06274	0.1486	38.64754
25.93333	57.89116	0.1531072	37.3519
25.95	59.07219	0.166611	36.07012
25.96667	59.44858	0.1350917	34.84376
25.98333	60.26499	0.1576055	33.64512
26	61.63596	0.1215834	32.45341
26.01667	61.77849	0.1305844	31.28248
26.03333	64.16696	0.1170807	30.13234
26.05	65.15169	0.1260817	29.00298
26.06667	66.48505	0.1260862	27.91519
26.08333	68.20741	0.1440972	26.86205
26.1	68.99815	0.1801238	25.84355
26.11667	69.28913	0.1350917	24.8597
26.13333	70.71513	0.1395945	23.88277
26.15	71.80551	0.1170762	22.91277
26.16667	72.4268	0.09456238	21.99127
26.18333	73.00923	0.09456238	21.08363
26.2	74.14139	0.1486	20.18984
26.21667	75.69089	0.1576055	19.35842
26.23333	76.43006	0.1305934	18.56163
26.25	78.17873	0.1395945	17.7787
26.26667	78.03107	0.1215834	16.99577
26.28333	79.55125	0.1350917	16.2267
26.3	79.17737	0.1260862	15.49227
26.31667	81.32601	0.1260862	14.80635
26.33333	81.62272	0.08105411	14.12735
26.35	82.44134	0.1936275	13.46913
26.36667	84.25698	0.1350962	12.86635
26.38333	84.82832	0.1486	12.27049
26.4	86.25139	0.1440972	11.67463
26.41667	86.29681	0.1395945	11.12728
26.43333	87.07645	0.1305889	10.58685
26.45	87.42145	0.1260862	10.04642
26.46667	87.95399	0.1215834	9.547565
26.48333	88.80442	0.1035679	9.09028
26.5	88.52454	0.1350872	8.639922
26.51667	90.72177	0.1260862	8.175709

26.53333	89.60782	0.1125734	7.718423
26.55	91.3726	0.1486	7.309638
26.56667	91.07047	0.1080751	6.935495
26.58333	91.12048	0.1305844	6.561352
26.6	92.96406	0.1395945	6.201067
26.61667	93.89567	0.1395945	5.840782
26.63333	94.28609	0.1080751	5.494353
26.65	93.34188	0.1080706	5.147925
26.66667	93.87824	0.1440972	4.808425
26.68333	96.57553	0.1576055	4.489711
26.7	95.25011	0.1621082	4.191783
26.71667	94.95871	0.1395945	3.91464
26.73333	97.33715	0.1305889	3.609783
26.75	97.38651	0.1215834	3.297998
26.76667	98.14436	0.1170762	3.006998
26.78333	96.58031	0.09456238	2.722927
26.8	97.24302	0.1035679	2.438855
26.81667	97.82945	0.1350917	2.168641
26.83333	96.80742	0.1395945	1.905356
26.85	98.11047	0.1621082	1.662856
26.86667	98.35333	0.1395945	1.420356
26.88333	98.08074	0.1621127	1.163999
26.9	95.91467	0.1125734	0.9353565
26.91667	100	0.08555687	0.7205709
26.93333	99.45768	0.1305844	0.4711425
26.95	98.73229	0.1080751	0.2217141
26.96667	97.50696	0.1305844	-0.01385713
26.98333	96.2935	0.1305889	-0.2355713
27	96.59982	0.1440972	-0.4295711
27.01667	97.8889	0.09456238	-0.6097139
27.03333	97.35553	0.1125734	-0.8175709
27.05	96.76528	0.1486	-1.039285
27.06667	97.03973	0.1621127	-1.240213
27.08333	95.55642	0.1215834	-1.420356
27.1	96.7417	0.1305844	-1.59357
27.11667	96.1949	0.1305889	-1.78757
27.13333	96.27542	0.1125779	-1.974641
27.15	95.07397	0.1305889	-2.133998
27.16667	93.68449	0.1756165	-2.279498
27.18333	94.4587	0.1350962	-2.404212
27.2	93.97296	0.175621	-2.549712
27.21667	93.86248	0.1711182	-2.743712
27.23333	94.00245	0.08555687	-2.923855
27.25	91.61643	0.1531027	-3.062426
27.26667	91.84169	0.1170807	-3.180212
27.28333	94.08536	0.1395945	-3.318783
27.3	91.84181	0.1395945	-3.450426

27.31667	91.22667	0.175621	-3.561283
27.33333	91.12562	0.09906964	-3.685997
27.35	90.0504	0.1170762	-3.81764
27.36667	89.41293	0.08105411	-3.949283
27.38333	89.42905	0.1215789	-4.094783
27.4	86.33615	0.1711182	-4.226426
27.41667	87.94634	0.1215834	-4.337283
27.43333	86.72901	0.09906514	-4.455068
27.45	86.3651	0.1260817	-4.572854
27.46667	85.81788	0.1440972	-4.676783
27.48333	85.44238	0.1260862	-4.794568
27.5	85.68072	0.1305889	-4.919282
27.51667	82.3009	0.1260862	-5.030139
27.53333	82.2871	0.1486	-5.140996
27.55	80.9125	0.1260862	-5.272639
27.56667	81.6309	0.1711182	-5.411211
27.58333	82.35485	0.1260862	-5.515139
27.6	79.60575	0.1395945	-5.584424
27.61667	79.67976	0.1531027	-5.660639
27.63333	78.77364	0.1350917	-5.764567
27.65	77.76802	0.1440972	-5.875424
27.66667	78.38973	0.1486	-5.979353
27.68333	77.26688	0.1080706	-6.055567
27.7	76.63712	0.1486	-6.13871
27.71667	75.29958	0.1350917	-6.242639
27.73333	75.01438	0.1711137	-6.346567
27.75	75.27295	0.139599	-6.436638
27.76667	73.25276	0.1080706	-6.52671
27.78333	73.68621	0.1305889	-6.616781
27.8	71.95807	0.1215834	-6.720709
27.81667	71.07004	0.1576055	-6.838495
27.83333	70.21967	0.1395945	-6.921638
27.85	71.07803	0.1170807	-6.977067
27.86667	69.74783	0.09906514	-7.004781
27.88333	68.71565	0.1305889	-7.060209
27.9	67.09871	0.1305889	-7.157209
27.91667	67.14735	0.1486	-7.274995
27.93333	66.93659	0.1305889	-7.371995
27.95	67.34832	0.1305889	-7.448209
27.96667	65.55512	0.1621082	-7.524423
27.98333	65.26396	0.1531072	-7.58678
28	65.2263	0.1576055	-7.662994
28.01667	65.2756	0.1350917	-7.725352
28.03333	62.82422	0.1395945	-7.794637
28.05	62.56309	0.1395945	-7.870852
28.06667	62.29987	0.1486	-7.953994
28.08333	61.50888	0.1666155	-8.037137

28.1	60.34037	0.1260862	-8.113352
28.11667	59.71694	0.1711182	-8.189566
28.13333	59.22272	0.1125779	-8.272708
28.15	59.02566	0.1080706	-8.341994
28.16667	58.65938	0.1530982	-8.404351
28.18333	57.65971	0.15761	-8.445923
28.2	57.65995	0.1576055	-8.487494
28.21667	56.32429	0.1486	-8.563708
28.23333	56.11453	0.1170807	-8.646851
28.25	55.26942	0.1440927	-8.702279
28.26667	55.92001	0.1215834	-8.750779
28.28333	54.60445	0.1486	-8.833922
28.3	54.20318	0.09005962	-8.910136
28.31667	54.36277	0.1846265	-8.958636
28.33333	51.93891	0.1440972	-9.000208
28.35	52.47843	0.1440972	-9.069493
28.36667	51.66668	0.09456238	-9.13185
28.38333	52.24256	0.1531027	-9.187279
28.4	50.51052	0.1080706	-9.249637
28.41667	51.08765	0.1215834	-9.311994
28.43333	49.76949	0.1395945	-9.367422
28.45	49.77069	0.1440972	-9.443636
28.46667	48.2701	0.1441017	-9.526779
28.48333	49.22478	0.1170762	-9.575279
28.5	48.7415	0.175621	-9.596065
28.51667	48.24776	0.1711182	-9.644565
28.53333	47.74554	0.1395945	-9.699993
28.55	47.25991	0.1080706	-9.741565
28.56667	46.47419	0.1260862	-9.783135
28.58333	45.84123	0.1395945	-9.845493
28.6	45.83574	0.1260862	-9.887064
28.61667	44.90115	0.1305889	-9.921707
28.63333	44.85263	0.1621082	-9.970207
28.65	44.69134	0.1666155	-10.03256
28.66667	43.49861	0.1215834	-10.08799
28.68333	42.99192	0.1080751	-10.14342
28.7	43.53919	0.1080706	-10.19885
28.71667	43.07094	0.1080706	-10.24042
28.73333	41.77127	0.1576055	-10.28199
28.75	42.6645	0.1486	-10.35821
28.76667	41.78732	0.1305889	-10.41364
28.78333	41.6203	0.1486	-10.44135
28.8	42.32092	0.1260862	-10.46214
28.81667	41.72102	0.1531027	-10.48985
28.83333	40.64562	0.193632	-10.53142
28.85	40.46165	0.09456688	-10.58685
28.86667	40.24784	0.06304309	-10.64921

28.88333	41.23239	0.1305844	-10.72542
28.9	38.97142	0.1801238	-10.79471
28.91667	38.94011	0.1170807	-10.82935
28.93333	38.84016	0.1486	-10.83628
28.95	39.27084	0.08555687	-10.85013
28.96667	38.37865	0.1486	-10.89171
28.98333	38.35567	0.1891293	-10.94713
29	38.00543	0.16662	-10.99564
29.01667	36.96106	0.1486045	-11.03721
29.03333	36.54491	0.1395945	-11.09956
29.05	37.32233	0.1486	-11.15499
29.06667	35.87847	0.1215789	-11.18271
29.08333	34.97493	0.1170807	-11.21042
29.1	35.94654	0.1035679	-11.24506
29.11667	35.32191	0.1440972	-11.28663
29.13333	35.01931	0.184631	-11.34899
29.15	34.53122	0.1531027	-11.38363
29.16667	34.22242	0.09005962	-11.39749
29.18333	34.32915	0.1305844	-11.41828
29.2	33.7986	0.1170807	-11.46678
29.21667	34.03988	0.1215789	-11.52221
29.23333	33.74758	0.1395945	-11.55685
29.25	33.62988	0.1170762	-11.59842
29.26667	33.12924	0.09005962	-11.62613
29.28333	33.50983	0.07655136	-11.64692
29.3	33.55526	0.1440927	-11.67463
29.31667	32.94474	0.1666155	-11.71621
29.33333	32.73277	0.1531072	-11.76471
29.35	32.86792	0.1035679	-11.82013
29.36667	32.06855	0.09906514	-11.86171
29.38333	32.26026	0.1440972	-11.88249
29.4	32.19434	0.1260862	-11.90328
29.41667	31.52572	0.1440972	-11.93099
29.43333	30.87686	0.1621082	-11.96563
29.45	30.82946	0.1125779	-12.01413
29.46667	31.08275	0.1440972	-12.05571
29.48333	30.92379	0.09456238	-12.08342
29.5	30.93161	0.1305889	-12.13885
29.51667	30.41487	0.1621082	-12.16656
29.53333	30.05223	0.1981348	-12.19428
29.55	30.71626	0.1305889	-12.24278
29.56667	29.44273	0.1350917	-12.27742
29.58333	29.23639	0.1170807	-12.29821
29.6	28.62118	0.1621082	-12.31899
29.61667	29.99648	0.1486045	-12.35363
29.63333	28.83189	0.1305889	-12.40213
29.65	29.37112	0.1305889	-12.44371

29.66667	29.42183	0.1170807	-12.46449
29.68333	28.37776	0.1260862	-12.47835
29.7	28.2424	0.1621082	-12.51299
29.71667	28.59327	0.1440972	-12.56842
29.73333	28.50219	0.1125779	-12.60999
29.75	28.01034	0.1170807	-12.63078
29.76667	27.79229	0.1440972	-12.67235
29.78333	27.6121	0.1486	-12.71392
29.8	26.81986	0.1440972	-12.72085
29.81667	27.03079	0.1305844	-12.72778
29.83333	25.96882	0.1350917	-12.74856
29.85	26.10835	0.1125734	-12.79013
29.86667	26.69516	0.1260862	-12.82478
29.88333	26.67704	0.1531027	-12.85942
29.9	26.34749	0.1080751	-12.88713
29.91667	26.60462	0.1215789	-12.92871
29.93333	25.76395	0.1621082	-12.97721
29.95	26.33429	0.1350917	-13.02571
29.96667	25.27874	0.1260862	-13.06035
29.98333	25.02186	0.1035679	-13.08806
30	26.39706	0.1350917	-13.1227
30.01667	25.15891	0.09456238	-13.16428
30.03333	24.52705	0.0720486	-13.21278
30.05	25.37254	0.1531027	-13.26128
30.06667	24.83891	0.1170807	-13.29592
30.08333	25.55566	0.1350917	-13.30978
30.1	24.41252	0.1215834	-13.32363
30.11667	24.70522	0.1260817	-13.35828
30.13333	24.27116	0.1035679	-13.39985
30.15	24.43765	0.09906514	-13.44142
30.16667	23.94022	0.1215789	-13.48299
30.18333	22.99503	0.1576055	-13.48992
30.2	23.4125	0.1395945	-13.49685
30.21667	23.77931	0.1215834	-13.52456
30.23333	23.39306	0.1260817	-13.55921
30.25	24.08746	0.1440972	-13.59385
30.26667	23.28701	0.1846265	-13.64235
30.28333	22.96254	0.1486	-13.67006
30.3	22.83124	0.1395945	-13.69778
30.31667	22.75484	0.1486	-13.72549
30.33333	22.56505	0.1125779	-13.76706
30.35	23.0721	0.06754585	-13.79478
30.36667	22.10985	0.1125734	-13.82942
30.38333	22.90712	0.193632	-13.88485
30.4	21.93463	0.15761	-13.91949
30.41667	22.16276	0.1486	-13.94028
30.43333	22.65253	0.1441017	-13.96799

30.45	22.59093	0.1260862	-14.00263
30.46667	22.03745	0.1305844	-14.02342
30.48333	21.65813	0.1215834	-14.05113
30.5	21.82574	0.1395945	-14.09963
30.51667	21.75973	0.1486	-14.13428
30.53333	21.75464	0.1350917	-14.1412
30.55	21.68645	0.1531027	-14.16892
30.56667	21.66222	0.1486	-14.21049
30.58333	21.26034	0.1711182	-14.24513
30.6	21.27968	0.1305889	-14.26592
30.61667	21.68422	0.1711182	-14.2867
30.63333	21.01417	0.1080706	-14.30056
30.65	21.80261	0.1395945	-14.32828
30.66667	20.70535	0.1350917	-14.36292
30.68333	20.69032	0.1350917	-14.39063
30.7	21.21593	0.1531027	-14.42528
30.71667	20.76355	0.1260862	-14.46685
30.73333	20.60579	0.1350872	-14.50842
30.75	21.03461	0.1260862	-14.5292
30.76667	20.7931	0.1576055	-14.54306
30.78333	20.37426	0.1170807	-14.56385
30.8	20.5425	0.1621082	-14.61235
30.81667	20.04992	0.1531072	-14.66085
30.83333	19.83576	0.1440972	-14.6747
30.85	20.59845	0.09005962	-14.69549
30.86667	19.69571	0.1440972	-14.7232
30.88333	19.70676	0.15761	-14.76478
30.9	19.92534	0.1170807	-14.79249
30.91667	20.12899	0.1215789	-14.80635
30.93333	19.63715	0.1080751	-14.79942
30.95	19.32325	0.1350917	-14.81328
30.96667	19.41677	0.09456238	-14.88256
30.98333	19.62664	0.1440972	-14.95185
31	18.89562	0.1440972	-14.97956
31.01667	18.95292	0.1440972	-14.9657
31.03333	18.92675	0.1395945	-14.9657
31.05	19.99213	0.1035679	-15.00035
31.06667	19.34189	0.1350872	-15.02113
31.08333	19.22692	0.1395945	-15.03499
31.1	19.13202	0.09906514	-15.05577
31.11667	18.63449	0.1215789	-15.09042
31.13333	18.34772	0.1440972	-15.13199
31.15	18.90702	0.1621082	-15.16663
31.16667	18.39981	0.1486	-15.2082
31.18333	18.44893	0.09005962	-15.2567
31.2	18.01013	0.1350917	-15.29135
31.21667	17.99139	0.09456238	-15.31906

31.23333	18.42513	0.1215789	-15.33985
31.25	18.47421	0.1125734	-15.36063
31.26667	18.55061	0.1170762	-15.37449
31.28333	18.62433	0.1486	-15.39528
31.3	18.36014	0.1350917	-15.42299
31.31667	17.75955	0.1305889	-15.46456
31.33333	18.10974	0.1350917	-15.4992
31.35	17.80609	0.1395945	-15.50613
31.36667	17.26188	0.1125734	-15.4992
31.38333	17.55538	0.1125734	-15.50613
31.4	17.74822	0.1125734	-15.54077
31.41667	17.13541	0.1395945	-15.58235
31.43333	16.72007	0.1350917	-15.61699
31.45	17.79788	0.1486	-15.63085
31.46667	17.20263	0.1260862	-15.65163
31.48333	17.17523	0.1035679	-15.67935
31.5	16.90017	0.1440972	-15.71399
31.51667	17.23405	0.1305889	-15.7417
31.53333	17.3621	0.1260862	-15.76942
31.55	16.89983	0.1395945	-15.80406
31.56667	16.28712	0.1125734	-15.81099
31.58333	16.45479	0.1711137	-15.81792
31.6	16.48148	0.1080751	-15.83177
31.61667	16.56557	0.1756165	-15.85256
31.63333	16.36672	0.09906964	-15.87335
31.65	16.78101	0.1035679	-15.90106
31.66667	16.56677	0.1305889	-15.94263
31.68333	16.03908	0.1395945	-15.99113
31.7	15.97416	0.1305889	-16.02577
31.71667	16.09441	0.1395945	-16.04656
31.73333	16.59275	0.166611	-16.05349
31.75	16.38132	0.1170807	-16.06042
31.76667	16.48022	0.1260862	-16.06735
31.78333	16.19339	0.1305889	-16.09506
31.8	15.8974	0.1486	-16.11584
31.81667	15.82468	0.1350917	-16.14356
31.83333	15.83525	0.1621082	-16.1782
31.85	15.4926	0.1170807	-16.20592
31.86667	15.75664	0.1531027	-16.21284
31.88333	15.26521	0.1486	-16.23363
31.9	15.1787	0.1170807	-16.26827
31.91667	15.74224	0.1621082	-16.28906
31.93333	15.76931	0.1080751	-16.28906
31.95	15.32559	0.1260817	-16.30984
31.96667	15.22088	0.1891293	-16.33063
31.98333	15.3457	0.09906964	-16.34449
32	14.89154	0.1485955	-16.39299

32.01667	15.24362	0.1125779	-16.44149
32.03333	15.24953	0.1395945	-16.48306
32.05	15.08216	0.211643	-16.51077
32.06667	14.78294	0.1215834	-16.5177
32.08333	14.80408	0.1260817	-16.51077
32.1	14.85234	0.1305889	-16.51077
32.11666	15.15248	0.1260862	-16.53849
32.13334	14.77668	0.0720486	-16.57313
32.15	15.01909	0.1215789	-16.59392
32.16667	14.56616	0.1621082	-16.60777
32.18333	14.34685	0.1486	-16.62163
32.2	14.11308	0.1801238	-16.62856
32.21667	14.59735	0.1305889	-16.63549
32.23333	14.37753	0.1035679	-16.67706
32.25	14.66559	0.1576055	-16.71863
32.26667	13.93956	0.09456238	-16.7602
32.28333	14.20036	0.1395945	-16.78792
32.3	14.15938	0.1035679	-16.79485
32.31667	14.03364	0.08555687	-16.80177
32.33333	14.0634	0.1170807	-16.81563
32.35	14.26747	0.07655136	-16.82949
32.36666	14.2316	0.1260862	-16.84335
32.38334	13.9261	0.09456238	-16.87106
32.4	14.29117	0.13959	-16.89877
32.41667	14.17766	0.1846265	-16.91263
32.43333	13.51798	0.1305889	-16.92649
32.45	13.64063	0.09005962	-16.9542
32.46667	13.54603	0.1395945	-16.96113
32.48333	13.6823	0.1576055	-16.96113
32.5	13.72032	0.1666155	-16.98885
32.51667	13.75541	0.1080751	-17.03042
32.53333	14.04371	0.09906514	-17.0512
32.55	13.51209	0.1035679	-17.07892
32.56667	13.43045	0.1395945	-17.12049
32.58333	13.23304	0.1170807	-17.13434
32.6	13.56803	0.1125734	-17.11356
32.61666	13.70141	0.08105411	-17.12742
32.63334	12.7535	0.1981348	-17.15513
32.65	13.70408	0.09906514	-17.17592
32.66667	13.01753	0.1215789	-17.20363
32.68333	13.19463	0.15761	-17.23134
32.7	12.9864	0.1305889	-17.23134
32.71667	13.40475	0.1125734	-17.25213
32.73333	13.20275	0.1305889	-17.27984
32.75	12.86019	0.166611	-17.2937
32.76667	13.26026	0.09906514	-17.31449
32.78333	13.29207	0.1531027	-17.34913

32.8	12.53024	0.1395945	-17.36992
32.81667	13.10828	0.1486	-17.35606
32.83333	12.75214	0.1440972	-17.34913
32.85	12.54686	0.1486045	-17.36992
32.86666	12.31168	0.1350917	-17.3907
32.88334	12.62813	0.08555687	-17.41149
32.9	12.51014	0.1440927	-17.4392
32.91667	12.86241	0.1531027	-17.47384
32.93333	12.63119	0.1080706	-17.49463
32.95	12.53439	0.1531027	-17.49463
32.96667	12.35277	0.09456238	-17.50156
32.98333	12.29962	0.1305844	-17.52927
33	12.41268	0.1846265	-17.55699
33.01667	12.16152	0.1350917	-17.5847
33.03333	12.56176	0.1305889	-17.61242
33.05	12.74297	0.09906514	-17.64013
33.06667	12.40878	0.1350917	-17.66784
33.08333	12.31731	0.1621127	-17.6817
33.1	12.05918	0.1215834	-17.6817
33.11666	12.36979	0.1080706	-17.68863
33.13334	12.38326	0.1486	-17.68863
33.15	12.24506	0.1305889	-17.6817
33.16667	12.18454	0.1305889	-17.68863
33.18333	12.29829	0.1260862	-17.72327
33.2	12.11332	0.1260862	-17.74406
33.21667	11.67454	0.1440972	-17.77177
33.23333	11.63111	0.1260862	-17.80642
33.25	11.75705	0.1260862	-17.8272
33.26667	12.29039	0.1215834	-17.83413
33.28333	12.24491	0.08555687	-17.85492
33.3	11.70424	0.1395945	-17.88956
33.31667	11.49961	0.1035679	-17.91035
33.33333	11.92447	0.1170807	-17.93113
33.35	11.52399	0.1260817	-17.93806
33.36666	11.69687	0.1440972	-17.96577
33.38334	11.36041	0.1035679	-17.99349
33.4	11.42708	0.1080706	-18.00735
33.41667	12.07273	0.1350917	-18.01427
33.43333	11.77116	0.1395945	-18.03506
33.45	11.32557	0.1260907	-18.03506
33.46667	11.14931	0.1170762	-18.04199
33.48333	11.2942	0.1441017	-18.07663
33.5	11.46169	0.1305844	-18.09049
33.51667	11.34852	0.1170762	-18.09049
33.53333	11.11435	0.1395945	-18.11127
33.55	11.20372	0.1170807	-18.12513
33.56667	11.03595	0.1531027	-18.13899

33.58333	10.76556	0.1350917	-18.15977
33.6	10.90238	0.1080706	-18.17363
33.61666	10.90925	0.1125734	-18.17363
33.63334	10.74136	0.1170762	-18.1667
33.65	10.69936	0.1215834	-18.18056
33.66667	10.7522	0.1125734	-18.20827
33.68333	11.26259	0.1305889	-18.22906
33.7	11.15023	0.1486	-18.23599
33.71667	10.24504	0.1170807	-18.24984
33.73333	10.69167	0.1170762	-18.25677
33.75	11.11098	0.1531027	-18.29834
33.76667	10.47304	0.1125779	-18.33992
33.78333	11.17139	0.1621082	-18.35377
33.8	10.61592	0.1395945	-18.3607
33.81667	10.87241	0.1350917	-18.38842
33.83333	10.24985	0.1215834	-18.42999
33.85	10.90105	0.1891248	-18.44384
33.86666	10.73519	0.1305889	-18.44384
33.88334	10.31573	0.1621082	-18.4577
33.9	10.22398	0.1666155	-18.48541
33.91667	10.45757	0.1080751	-18.47849
33.93333	10.88938	0.09906514	-18.47156
33.95	10.57942	0.1891293	-18.48541
33.96667	10.41723	0.09456238	-18.52006
33.98333	10.25443	0.1080706	-18.54777
34	10.06819	0.1260817	-18.56856
34.01667	10.44498	0.1305889	-18.58241
34.03333	10.54206	0.09456238	-18.61013
34.05	10.31492	0.1260862	-18.63784
34.06667	10.45211	0.1531027	-18.64477
34.08333	9.990635	0.1260862	-18.6517
34.1	9.719813	0.04953032	-18.64477
34.11666	9.994556	0.1350872	-18.65863
34.13334	9.355157	0.08555687	-18.67249
34.15	10.42419	0.1215789	-18.67249
34.16667	9.822667	0.1215789	-18.67941
34.18333	9.408207	0.1215789	-18.7002
34.2	10.19778	0.08555687	-18.72791
34.21667	9.426609	0.1125734	-18.76256
34.23333	9.783607	0.1260817	-18.79027
34.25	9.677567	0.1215789	-18.80413
34.26667	9.701244	0.1395945	-18.81799
34.28333	9.264145	0.1215834	-18.82491
34.3	9.447721	0.1125779	-18.83184
34.31667	9.503457	0.1621082	-18.86649
34.33333	9.106892	0.09906514	-18.88727
34.35	9.555893	0.1215789	-18.88727

34.36666	9.125478	0.1486	-18.8942
34.38334	9.406339	0.1305889	-18.90806
34.4	10.01705	0.1395945	-18.92192
34.41667	9.726134	0.08555687	-18.9427
34.43333	9.437604	0.09456238	-18.94963
34.45	9.227675	0.13959	-18.94963
34.46667	9.537683	0.08555687	-18.9427
34.48333	9.305204	0.1531027	-18.94963
34.5	9.40071	0.1170807	-18.97042
34.51667	8.837263	0.1035679	-19.00506
34.53333	9.4719	0.09005962	-19.03277
34.55	9.370431	0.09906514	-19.06049
34.56667	9.256928	0.1125734	-19.06049
34.58333	9.058867	0.1035679	-19.07434
34.6	9.213816	0.1350917	-19.11592
34.61666	9.37625	0.1125734	-19.15749
34.63334	9.287095	0.1666155	-19.15749
34.65	9.538011	0.1215834	-19.12284
34.66667	9.197479	0.1170807	-19.12284
34.68333	9.358344	0.1576055	-19.17134
34.7	8.675713	0.1666155	-19.19213
34.71667	8.961665	0.1395945	-19.19906
34.73333	9.046439	0.1440972	-19.19213
34.75	8.764642	0.1621127	-19.19906
34.76667	8.884059	0.1125779	-19.22677
34.78333	9.045801	0.1170762	-19.24756
34.8	8.615338	0.1350917	-19.26142
34.81667	8.991527	0.1260862	-19.27527
34.83333	8.435201	0.1215834	-19.2822
34.85	8.643642	0.1125734	-19.30299
34.86666	9.039438	0.1305889	-19.32377
34.88334	8.153809	0.1305889	-19.34456
34.9	8.70287	0.1531027	-19.34456
34.91667	7.853829	0.1260862	-19.35149
34.93333	8.162868	0.1756165	-19.36534
34.95	8.607292	0.1260862	-19.38613
34.96667	8.77554	0.08555687	-19.39999
34.98333	8.782124	0.1305844	-19.41384
35	8.452838	0.1260862	-19.4277
35.01667	8.46998	0.1531027	-19.4277
35.03333	8.643708	0.09906514	-19.40691
35.05	8.266762	0.1440972	-19.41384
35.06667	7.919188	0.1215789	-19.44849
35.08333	8.348391	0.1305889	-19.46234
35.1	8.104208	0.202642	-19.46234
35.11666	8.253845	0.15761	-19.48313
35.13334	7.960527	0.1035679	-19.51084

35.15	7.953968	0.1801238	-19.50391
35.16667	8.360459	0.1260862	-19.51084
35.18333	7.954672	0.09456238	-19.5247
35.2	8.522416	0.1486	-19.53856
35.21667	7.853674	0.1305889	-19.55934
35.23333	8.13321	0.1395945	-19.58013
35.25	7.930272	0.1350917	-19.58706
35.26667	7.510786	0.1260862	-19.60091
35.28333	7.845061	0.1125734	-19.60784
35.3	7.726062	0.1395945	-19.61477
35.31667	7.881393	0.1260862	-19.6217
35.33333	7.840018	0.1305844	-19.63556
35.35	7.821813	0.1305889	-19.66327
35.36666	7.618444	0.1305844	-19.70484
35.38334	7.959029	0.193632	-19.7187
35.4	7.738202	0.1215834	-19.69791
35.41667	8.013321	0.1395945	-19.69099
35.43333	7.702652	0.1215834	-19.71177
35.45	7.755344	0.1215789	-19.73256
35.46667	7.516796	0.1080706	-19.74641
35.48333	7.940806	0.1260862	-19.75334
35.5	7.863642	0.1486	-19.76027
35.51667	7.924046	0.1576055	-19.78799
35.53333	7.731893	0.09906964	-19.80877
35.55	7.318806	0.1260817	-19.80877
35.56667	7.330009	0.1440972	-19.80184
35.58333	7.557175	0.1621082	-19.80184
35.6	7.306982	0.1035724	-19.79491
35.61666	7.395236	0.1440927	-19.80184
35.63334	7.204122	0.1125779	-19.82956
35.65	7.461502	0.1486	-19.85727
35.66667	7.653296	0.1125779	-19.8642
35.68333	7.043114	0.1486	-19.87113
35.7	7.221532	0.1486045	-19.87113
35.71667	7.245915	0.08555687	-19.87113
35.73333	7.718469	0.1485955	-19.89191
35.75	7.246977	0.07655136	-19.89884
35.76667	7.449337	0.1080706	-19.9127
35.78333	7.144428	0.1350917	-19.91963
35.8	7.287452	0.1260862	-19.92656
35.81667	7.222141	0.1080751	-19.92656
35.83333	7.060543	0.1080706	-19.94734
35.85	6.802476	0.1486	-19.96813
35.86666	7.119293	0.1170807	-19.96813
35.88334	6.850835	0.1125734	-19.97506
35.9	6.91583	0.1260862	-19.98199
35.91667	7.089312	0.1350917	-19.98891

35.93333	7.257697	0.1215834	-19.98199
35.95	6.886606	0.1035679	-19.97506
35.96667	7.20331	0.1395945	-19.99584
35.98333	7.401205	0.1305889	-20.03741
36	6.665702	0.1305889	-20.06513
36.01667	6.723701	0.1035679	-20.06513
36.03333	6.634771	0.1305844	-20.0582
36.05	6.867721	0.1170807	-20.06513
36.06667	6.509081	0.1576055	-20.06513
36.08333	6.998742	0.1170762	-20.0582
36.1	7.16166	0.1576055	-20.07899
36.11666	6.700834	0.139599	-20.1067
36.13334	6.885532	0.166611	-20.11363
36.15	6.784612	0.1621127	-20.11363
36.16667	6.694424	0.1305889	-20.12056
36.18333	6.772059	0.08105411	-20.12056
36.2	6.969578	0.1215789	-20.12056
36.21667	6.455147	0.1350917	-20.14827
36.23333	6.926782	0.1576055	-20.18291
36.25	6.892247	0.1440972	-20.2037
36.26667	6.419346	0.1215834	-20.21756
36.28333	6.975016	0.1440972	-20.21756
36.3	6.365311	0.1260862	-20.22449
36.31667	6.502276	0.09005962	-20.24527
36.33333	6.352526	0.1305844	-20.2522
36.35	6.592071	0.1260862	-20.2522
36.36666	6.238952	0.1170762	-20.24527
36.38334	6.615773	0.1576055	-20.2522
36.4	6.675741	0.1260862	-20.27991
36.41667	6.407158	0.1486	-20.30763
36.43333	6.3467	0.1170807	-20.31456
36.45	6.214838	0.1170807	-20.32841
36.46667	6.238504	0.09906514	-20.34227
36.48333	6.172621	0.184622	-20.3492
36.5	6.435742	0.1305889	-20.3492
36.51667	6.304841	0.1486	-20.36306
36.53333	6.256631	0.1215789	-20.36999
36.55	6.316325	0.1576055	-20.36306
36.56667	6.424151	0.1260862	-20.36999
36.58333	6.089374	0.1170807	-20.39077
36.6	6.118866	0.1260862	-20.41849
36.61666	6.154428	0.1486	-20.41849
36.63334	6.406084	0.1486	-20.3977
36.65	6.358423	0.1080751	-20.36999
36.66667	6.412333	0.1170807	-20.38384
36.68333	6.268545	0.1170762	-20.43234
36.7	5.92759	0.1260817	-20.46699

36.71667	5.84315	0.1305889	-20.46699
36.73333	6.04625	0.07655136	-20.4462
36.75	6.148698	0.1125734	-20.4462
36.76667	5.903452	0.1486	-20.46006
36.78333	5.77747	0.1531072	-20.47391
36.8	6.087703	0.1170807	-20.4947
36.81667	6.172621	0.1801192	-20.51549
36.83333	6.035136	0.1531072	-20.4947
36.85	6.028564	0.1260862	-20.48084
36.86666	6.100601	0.1440927	-20.51549
36.88334	5.783898	0.15761	-20.52241
36.9	6.033954	0.1260862	-20.51549
36.91667	5.855547	0.1486	-20.5432
36.93333	6.052403	0.1305889	-20.57091
36.95	5.538187	0.1440972	-20.57784
36.96667	5.878515	0.1531027	-20.58477
36.98333	5.896749	0.1350917	-20.58477
37	5.669697	0.1801238	-20.5917
37.01667	5.836728	0.1440972	-20.61249
37.03333	6.040514	0.1260862	-20.61941
37.05	5.968626	0.1125734	-20.61249
37.06667	5.933016	0.1080706	-20.61941
37.08333	5.675564	0.1260862	-20.6402
37.1	5.735049	0.1846265	-20.66099
37.11666	5.783009	0.1395945	-20.6887
37.13334	5.717418	0.1440972	-20.69563
37.15	5.645416	0.1621127	-20.68177
37.16667	5.711001	0.1260862	-20.6887
37.18333	5.956438	0.1260817	-20.70256
37.2	5.705671	0.1395945	-20.70949
37.21667	5.866804	0.1260862	-20.71641
37.23333	5.56787	0.175621	-20.71641
37.25	5.675111	0.15761	-20.72334
37.26667	5.244374	0.09906514	-20.7372
37.28333	6.045247	0.09906514	-20.7372
37.3	5.454535	0.1350917	-20.73027
37.31667	5.435578	0.166611	-20.7372
37.33333	5.585227	0.1395945	-20.75799
37.35	5.579432	0.1305889	-20.79263
37.36666	5.842422	0.175621	-20.80648
37.38334	5.490103	0.1125779	-20.80648
37.4	5.668939	0.1440972	-20.81341
37.41667	5.758972	0.1486	-20.82727
37.43333	5.477777	0.1395945	-20.82034
37.45	5.465565	0.1666155	-20.82727
37.46667	5.184578	0.1440972	-20.84806
37.48333	5.494692	0.08555687	-20.8827

37.5	5.322147	0.09906514	-20.90348
37.51667	5.39955	0.1035679	-20.88963
37.53333	5.57933	0.09906514	-20.86191
37.55	5.316089	0.09906514	-20.86884
37.56667	5.26197	0.1486	-20.88963
37.58333	4.980649	0.1621127	-20.90348
37.6	5.345264	0.1215834	-20.91734
37.61666	5.519165	0.1440972	-20.92427
37.63334	5.202461	0.1260862	-20.91734
37.65	5.243598	0.1531072	-20.91734
37.66667	5.24407	0.1756165	-20.92427
37.68333	5.375406	0.1170807	-20.9312
37.7	5.327775	0.1666155	-20.92427
37.71667	5.369599	0.1440972	-20.92427
37.73333	5.190392	0.1576055	-20.94506
37.75	5.171997	0.1350917	-20.95198
37.76667	5.411326	0.1035679	-20.92427
37.78333	5.399747	0.1486	-20.91041
37.8	4.927097	0.1305889	-20.9312
37.81667	5.105946	0.1170807	-20.94506
37.83333	5.423228	0.08105411	-20.96584
37.85	5.160346	0.166611	-20.99356
37.86666	5.058309	0.1350917	-21.00741
37.88334	5.309422	0.09906514	-21.02127
37.9	5.357637	0.1125734	-21.03513
37.91667	4.532388	0.1035679	-21.05591
37.93333	5.446023	0.1891248	-21.05591
37.95	4.9452	0.1035724	-21.04898
37.96667	5.231421	0.1486	-21.03513
37.98333	5.632661	0.1486045	-21.04206
38	5.118809	0.09906514	-21.04206
38.01667	5.118152	0.1215789	-21.05591
38.03333	4.693593	0.09005962	-21.08363
38.05	5.105486	0.07655136	-21.08363
38.06667	5.105886	0.166611	-21.0767
38.08333	4.956846	0.1395945	-21.08363
38.1	5.17159	0.175621	-21.09056
38.11666	5.12418	0.1170807	-21.09056
38.13334	5.196122	0.1621082	-21.10441
38.15	4.860784	0.1305889	-21.13906
38.16667	5.314972	0.1576055	-21.1737
38.18333	5.256019	0.1215834	-21.1737
38.2	4.998758	0.1035679	-21.15984
38.21667	4.87872	0.1215789	-21.1737
38.23333	4.848602	0.1576055	-21.19448
38.25	4.765039	0.1486	-21.19448
38.26667	5.165341	0.1125779	-21.19448

38.28333	4.759267	0.13959	-21.20141
38.3	4.717068	0.1215834	-21.2222
38.31667	5.003874	0.1395945	-21.23606
38.33333	5.058196	0.1486	-21.25684
38.35	4.705279	0.1035724	-21.26377
38.36666	4.848381	0.1440972	-21.26377
38.38334	4.956326	0.1170807	-21.25684
38.4	4.645454	0.1440972	-21.26377
38.41667	5.087401	0.1080706	-21.28456
38.43333	4.849002	0.1440972	-21.30534
38.45	4.699018	0.1305889	-21.31227
38.46667	4.74678	0.0720486	-21.31227
38.48333	4.824614	0.13959	-21.29841
38.5	4.884367	0.09456238	-21.28456
38.51667	4.621418	0.1035679	-21.29148
38.53333	4.66893	0.1305889	-21.29841
38.55	4.645251	0.1260817	-21.28456
38.56667	4.692709	0.1350917	-21.29148
38.58333	4.382195	0.1576055	-21.33998
38.6	4.572695	0.1395945	-21.36077
38.61666	4.609075	0.07655136	-21.3677
38.63334	4.740734	0.1125734	-21.38848
38.65	4.633099	0.1080706	-21.40234
38.66667	4.985961	0.1576055	-21.39541
38.68333	4.765087	0.1035679	-21.40234
38.7	4.866275	0.1215834	-21.4162
38.71667	4.848799	0.1350872	-21.43006
38.73333	4.669133	0.175621	-21.43699
38.75	4.501607	0.1305889	-21.42313
38.76667	4.59694	0.1576055	-21.40234
38.78333	4.609105	0.1260862	-21.40234
38.8	4.429707	0.1215834	-21.4162
38.81667	4.381748	0.1350917	-21.4162
38.83333	4.555027	0.1621127	-21.40927
38.85	4.375737	0.1125779	-21.40927
38.86666	4.453325	0.07655136	-21.44391
38.88334	4.614864	0.1080706	-21.47163
38.9	4.507528	0.1260862	-21.48549
38.91667	4.722571	0.1486	-21.47163
38.93333	4.334129	0.09005962	-21.4647
38.95	4.279837	0.1395945	-21.4647
38.96667	4.656508	0.1035679	-21.47856
38.98333	4.561253	0.1440972	-21.48549
39	4.36408	0.1350917	-21.47856
39.01667	4.549005	0.1576055	-21.49934
39.03333	4.459437	0.15761	-21.5132
39.05	4.734473	0.1215834	-21.5132

39.06667	4.262372	0.1080706	-21.52013
39.08333	4.441316	0.1305889	-21.52706
39.1	4.399678	0.1305889	-21.53399
39.11666	4.214246	0.1395945	-21.53399
39.13334	4.363364	0.1486	-21.52706
39.15	4.262151	0.1350917	-21.52706
39.16667	3.968869	0.1666155	-21.55477
39.18333	4.685917	0.09005962	-21.58249
39.2	4.519585	0.1531027	-21.58249
39.21667	4.447625	0.1305889	-21.57556
39.23333	4.357777	0.09906964	-21.59634
39.25	4.429379	0.1215789	-21.61713
39.26667	3.981093	0.1576055	-21.61713
39.28333	4.458721	0.1711182	-21.62406
39.3	4.184342	0.1125734	-21.64484
39.31667	4.3455	0.1395945	-21.66563
39.33333	4.136652	0.1305889	-21.66563
39.35	4.410959	0.08555687	-21.6587
39.36666	4.327879	0.1486	-21.6587
39.38334	4.764	0.09456238	-21.63791
39.4	4.226649	0.1215834	-21.62406
39.41667	4.782181	0.09906514	-21.63099
39.43333	4.346126	0.1305889	-21.64484
39.45	4.273892	0.1531072	-21.6587
39.46667	4.656467	0.1531027	-21.6587
39.48333	4.370007	0.1486045	-21.64484
39.5	4.375433	0.1080751	-21.6587
39.51667	4.549077	0.1756165	-21.66563
39.53333	4.244251	0.09906514	-21.6587
39.55	4.1542	0.1080706	-21.6587
39.56667	4.261781	0.1080706	-21.67949
39.58333	4.554932	0.1305889	-21.70027
39.6	4.489287	0.1035679	-21.71413
39.61666	4.005154	0.1080706	-21.71413
39.63334	4.351295	0.09005962	-21.70027
39.65	4.381407	0.09906514	-21.69334
39.66667	4.345881	0.1035679	-21.68641
39.68333	4.261751	0.1170807	-21.68641
39.7	3.993061	0.1305889	-21.70027
39.71667	4.135828	0.1395945	-21.7072
39.73333	4.429164	0.1215789	-21.71413
39.75	4.238186	0.08555687	-21.72799
39.76667	3.933164	0.09906514	-21.74877
39.78333	4.315244	0.1170762	-21.7557
39.8	4.106402	0.1305889	-21.76263
39.81667	4.064358	0.1531027	-21.76956
39.83333	4.040584	0.1305889	-21.78341

39.85	4.159864	0.1440972	-21.79727
39.86666	4.213989	0.1891293	-21.81806
39.88334	4.130241	0.1215834	-21.81113
39.9	3.998647	0.1215834	-21.8042
39.91667	4.076277	0.1531027	-21.79727
39.93333	4.345273	0.1260862	-21.79727
39.95	4.501052	0.09456238	-21.81806
39.96667	3.849655	0.09906514	-21.8527
39.98333	4.004336	0.1531027	-21.87348
40	4.136061	0.1215834	-21.85963
40.01667	3.92097	0.09906514	-21.86656
40.03333	3.669636	0.13959	-21.86656
40.05	3.938279	0.1125734	-21.86656
40.06667	4.225813	0.1440972	-21.89427
40.08333	4.094315	0.1486	-21.89427
40.1	4.219809	0.1531027	-21.88734
40.11666	4.219946	0.08105411	-21.87348
40.13334	4.136377	0.1531027	-21.85963
40.15	4.028688	0.1215789	-21.86656
40.16667	4.141827	0.1260862	-21.89427
40.18333	4.16609	0.1440972	-21.92198
40.2	4.219809	0.193632	-21.92891
40.21667	4.243851	0.1080751	-21.91506
40.23333	3.944988	0.1305844	-21.9012
40.25	4.058305	0.1891293	-21.90813
40.26667	4.165958	0.15761	-21.92891
40.28333	4.231776	0.1305889	-21.93584
40.3	4.136347	0.1395945	-21.91506
40.31667	3.920994	0.1215834	-21.9012
40.33333	3.944523	0.1711137	-21.91506
40.35	3.855027	0.1440972	-21.9497
40.36666	3.825093	0.1170807	-21.97741
40.38334	3.836774	0.1035724	-21.98434
40.4	4.010316	0.1621082	-21.98434
40.41667	4.076236	0.1666155	-21.97741
40.43333	3.902908	0.1531027	-21.97048
40.45	3.920624	0.1215834	-21.98434
40.46667	3.759365	0.1260862	-21.97741
40.48333	4.105948	0.1215834	-21.97048
40.5	3.861091	0.1305889	-21.9982
40.51667	4.088	0.1170762	-22.03977
40.53333	3.890995	0.1486	-22.06748
40.55	3.675713	0.09906514	-22.07441
40.56667	3.80086	0.1215789	-22.0952
40.58333	3.926563	0.1440972	-22.0952
40.6	4.034269	0.07655136	-22.08134
40.61666	4.088245	0.07655136	-22.08134

40.63334	4.010364	0.1576055	-22.06748
40.65	4.136079	0.1170807	-22.05363
40.66667	3.885139	0.1440972	-22.05363
40.68333	4.141767	0.1260862	-22.0467
40.7	3.652076	0.1305889	-22.05363
40.71667	4.159488	0.166611	-22.08134
40.73333	3.861228	0.1666155	-22.0952
40.75	4.105823	0.1621127	-22.08827
40.76667	3.789507	0.1350917	-22.0952
40.78333	3.61569	0.1170762	-22.11598
40.8	4.021765	0.1305889	-22.11598
40.81667	3.920964	0.175621	-22.12984
40.83333	3.884757	0.1395945	-22.1437
40.85	4.016369	0.1035679	-22.15756
40.86666	3.681526	0.09906514	-22.15756
40.88334	3.783037	0.1260817	-22.1437
40.9	3.962363	0.1395945	-22.1437
40.91667	3.861055	0.1080706	-22.15756
40.93333	3.62767	0.1350917	-22.16448
40.95	3.800812	0.1035679	-22.17834
40.96667	3.884775	0.1350917	-22.17834
40.98333	3.896599	0.1666155	-22.17141
41	3.66368	0.08555687	-22.16448
41.01667	3.968236	0.1440972	-22.17141
41.03333	3.866958	0.09906514	-22.17141
41.05	3.872963	0.1035679	-22.1922
41.06667	3.795208	0.1215789	-22.20605
41.08333	3.753145	0.1531072	-22.21298
41.1	3.651473	0.1125734	-22.21298
41.11666	3.76494	0.1260862	-22.21991
41.13334	3.693476	0.1215834	-22.22684
41.15	3.79479	0.1080706	-22.21991
41.16667	3.878705	0.13959	-22.20605
41.18333	3.849022	0.1125779	-22.21991
41.2	3.783127	0.1440972	-22.22684
41.21667	3.902693	0.1125779	-22.23377
41.23333	4.052253	0.1035679	-22.26148
41.25	3.777349	0.1576055	-22.29613
41.26667	3.800956	0.1350917	-22.30998
41.28333	3.777074	0.1440972	-22.31691
41.3	3.795166	0.1440972	-22.32384
41.31667	3.520005	0.1215834	-22.30998
41.33333	3.597551	0.1260817	-22.28227
41.35	3.537858	0.1531027	-22.26841
41.36666	3.938208	0.1395945	-22.2892
41.38334	3.842779	0.1440972	-22.30998
41.4	3.735269	0.09456238	-22.33077

41.41667	3.812965	0.1350917	-22.3377
41.43333	3.633674	0.1170807	-22.34463
41.45	3.60956	0.1035679	-22.34463
41.46667	3.621606	0.1350872	-22.34463
41.48333	3.555776	0.1035679	-22.32384
41.5	4.135291	0.1260817	-22.30998
41.51667	3.873237	0.1756165	-22.32384
41.53333	3.705389	0.1531072	-22.32384
41.55	3.723331	0.1170807	-22.32384
41.56667	3.681311	0.1215834	-22.34463
41.58333	3.7829	0.1711182	-22.35848
41.6	3.801111	0.1621127	-22.35848
41.61666	3.806912	0.1350917	-22.35155
41.63334	3.442429	0.15761	-22.34463
41.65	3.758816	0.1486	-22.34463
41.66667	3.4961	0.1260817	-22.36541
41.68333	3.728936	0.1395945	-22.37927
41.7	3.699253	0.1486	-22.39313
41.71667	3.597701	0.09456238	-22.40005
41.73333	3.705145	0.1350872	-22.41391
41.75	3.681442	0.1125734	-22.40698
41.76667	3.627521	0.1170807	-22.39313
41.78333	3.872551	0.1260817	-22.37927
41.8	3.968493	0.1125779	-22.37927
41.81667	3.855104	0.1125734	-22.3862
41.83333	3.585787	0.1576055	-22.39313
41.85	3.693291	0.1035724	-22.39313
41.86666	3.573676	0.1170762	-22.40005
41.88334	3.579657	0.1125779	-22.4347
41.9	3.597581	0.1260862	-22.45548
41.91667	3.633364	0.1531027	-22.44163
41.93333	3.91438	0.166611	-22.4347
41.95	3.615899	0.1080751	-22.42777
41.96667	3.866457	0.2071403	-22.44163
41.98333	3.490346	0.1350917	-22.45548
42	3.639291	0.1080706	-22.46241
42.01667	3.681281	0.1756165	-22.46934
42.03333	3.394469	0.1215834	-22.47627
42.05	3.693004	0.1080706	-22.49013
42.06667	3.38252	0.1170762	-22.49706
42.08333	3.692855	0.1260862	-22.49706
42.1	3.322778	0.1260862	-22.49013
42.11666	3.705025	0.1260862	-22.49706
42.13334	3.699104	0.1440972	-22.5317
42.15	3.520053	0.1035679	-22.53863
42.16667	3.716975	0.1305889	-22.51784
42.18333	3.579746	0.09456238	-22.51784

42.2	3.591708	0.1531027	-22.54556
42.21667	3.818635	0.08555687	-22.55248
42.23333	3.609704	0.1305844	-22.5317
42.25	3.615589	0.1215834	-22.51784
42.26667	3.471992	0.1395945	-22.51091
42.28333	3.633364	0.1305889	-22.51784
42.3	3.87855	0.1125779	-22.53863
42.31667	3.376671	0.1440972	-22.54556
42.33333	3.435994	0.09456238	-22.53863
42.35	3.615332	0.1215789	-22.53863
42.36666	3.37648	0.1080706	-22.55248
42.38334	3.579454	0.1531027	-22.55248
42.4	3.561578	0.09456238	-22.53863
42.41667	3.466053	0.1440972	-22.5317
42.43333	3.471974	0.09906514	-22.55941
42.45	3.752805	0.09005962	-22.57327
42.46667	3.561918	0.1260817	-22.57327
42.48333	3.603478	0.1215834	-22.58713
42.5	3.406246	0.1170807	-22.5802
42.51667	3.465904	0.1531027	-22.57327
42.53333	3.752829	0.1621082	-22.58713
42.55	3.537989	0.1035724	-22.60791
42.56667	3.45405	0.1305844	-22.60791
42.58333	3.651187	0.1711137	-22.60791
42.6	3.675313	0.1080751	-22.61484
42.61666	3.472171	0.09906514	-22.6287
42.63334	3.687029	0.1170807	-22.6287
42.65	3.513983	0.1260817	-22.60791
42.66667	3.495909	0.1576055	-22.60098
42.68333	3.489851	0.139599	-22.60098
42.7	3.352414	0.1621082	-22.60098
42.71667	3.489761	0.1080706	-22.60098
42.73333	3.489875	0.1305889	-22.60791
42.75	3.442106	0.07655136	-22.60098
42.76667	3.513696	0.1080706	-22.60098
42.78333	3.316565	0.1080706	-22.62177
42.8	3.394129	0.1711182	-22.63563
42.81667	3.758768	0.1080706	-22.63563
42.83333	3.364596	0.1801192	-22.6287
42.85	3.38825	0.1441017	-22.6287
42.86666	3.304383	0.1125779	-22.6287
42.88334	3.346302	0.1080706	-22.63563
42.9	3.7947	0.1170762	-22.63563
42.91667	3.627616	0.1080706	-22.64256
42.93333	3.51985	0.1080706	-22.64948
42.95	3.453978	0.1215834	-22.65641
42.96667	3.531764	0.1350872	-22.64948

42.98333	3.633286	0.1711182	-22.64256
43	3.358645	0.1260862	-22.6287
43.01667	3.406109	0.1395945	-22.64256
43.03333	3.471861	0.1440972	-22.6772
43.05	3.059531	0.1350917	-22.68413
43.06667	3.429703	0.1305889	-22.6772
43.08333	3.71097	0.1125779	-22.69106
43.1	3.388602	0.1440972	-22.69798
43.11666	3.662987	0.1395945	-22.70491
43.13334	3.274909	0.1215789	-22.69798
43.15	3.788403	0.1305889	-22.68413
43.16667	3.406586	0.0720486	-22.69798
43.18333	3.214857	0.1080706	-22.73263
43.2	3.60315	0.1440972	-22.74648
43.21667	3.322653	0.1080706	-22.76034
43.23333	3.76448	0.1305889	-22.76727
43.25	3.49619	0.1440972	-22.7742
43.26667	3.537798	0.1035724	-22.76034
43.28333	3.53772	0.1215789	-22.75341
43.3	3.65139	0.1170807	-22.74648
43.31667	3.412298	0.1215789	-22.75341
43.33333	3.645128	0.1125734	-22.76034
43.35	3.567606	0.07655136	-22.76727
43.36666	3.543832	0.13959	-22.76727
43.38334	3.639357	0.1395945	-22.75341
43.4	3.579639	0.1080751	-22.75341
43.41667	3.537893	0.184622	-22.76034
43.43333	3.573515	0.1486045	-22.76034
43.45	3.346522	0.1215834	-22.75341
43.46667	3.663095	0.1666155	-22.7257
43.48333	3.705288	0.1170807	-22.69798
43.5	3.496046	0.1350917	-22.69798
43.51667	3.454044	0.1260862	-22.73956
43.53333	3.328449	0.1260817	-22.76034
43.55	3.657144	0.1035679	-22.76034
43.56667	3.322635	0.1486	-22.76727
43.58333	3.471682	0.1080706	-22.76034
43.6	3.436185	0.166611	-22.76034
43.61666	3.441933	0.1440972	-22.78113
43.63334	3.585423	0.15761	-22.79498
43.65	3.322743	0.1440972	-22.80191
43.66667	3.64492	0.1080751	-22.81577
43.68333	3.352569	0.1440927	-22.81577
43.7	3.418154	0.1125779	-22.81577
43.71667	3.406061	0.1576055	-22.78806
43.73333	3.675128	0.1305889	-22.79498
43.75	3.304806	0.1486	-22.81577

43.76667	3.316452	0.1260862	-22.80884
43.78333	3.435821	0.1305889	-22.80191
43.8	3.585411	0.1440972	-22.80884
43.81667	3.663441	0.1486	-22.8227
43.83333	3.364447	0.09906514	-22.83656
43.85	3.340303	0.1305889	-22.82963
43.86666	3.376235	0.1395945	-22.80884
43.88334	3.376122	0.1170762	-22.80191
43.9	3.424146	0.1260862	-22.79498
43.91667	3.346391	0.1531072	-22.78806
43.93333	3.364124	0.1486	-22.78806
43.95	3.173004	0.1531072	-22.79498
43.96667	3.184745	0.1260862	-22.79498
43.98333	3.364154	0.15761	-22.80191
44	3.627168	0.1350917	-22.81577
44.01667	3.794909	0.1486	-22.80191
44.03333	3.251147	0.1486	-22.79498
44.05	3.26851	0.1305889	-22.79498
44.06667	3.232751	0.1035679	-22.78806
44.08333	3.603007	0.1531027	-22.78113
44.1	3.663459	0.1260862	-22.78113
44.11666	3.418279	0.1711182	-22.78113
44.13334	3.238714	0.1035679	-22.78806
44.15	3.453805	0.1215834	-22.78806
44.16667	3.262798	0.1621082	-22.7742
44.18333	3.035477	0.1215834	-22.76727
44.2	3.525126	0.1486	-22.76727
44.21667	3.418213	0.1170807	-22.76727
44.23333	3.501782	0.09456238	-22.76034
44.25	3.478003	0.1260817	-22.73956
44.26667	3.49567	0.1440972	-22.73263
44.28333	3.436281	0.1035679	-22.73263
44.3	3.310471	0.1080751	-22.74648
44.31667	3.298533	0.1350917	-22.75341
44.33333	3.531399	0.1440972	-22.73263
44.35	3.37639	0.1486045	-22.71877
44.36666	3.561506	0.1080706	-22.7257
44.38334	3.627401	0.07655136	-22.73956
44.4	3.221029	0.1305844	-22.75341
44.41667	3.752674	0.1170807	-22.73956
44.43333	3.245053	0.09456238	-22.73263
44.45	3.579221	0.1621082	-22.74648
44.46667	3.364614	0.1080706	-22.76034
44.48333	3.423872	0.1395945	-22.76034
44.5	3.567546	0.193632	-22.76034
44.51667	3.376426	0.1305889	-22.76034
44.53333	3.663083	0.1395945	-22.73956

44.55	3.454211	0.1215834	-22.73263
44.56667	3.244695	0.1170762	-22.75341
44.58333	3.214845	0.1215834	-22.74648
44.6	3.471646	0.1440972	-22.7257
44.61666	3.686898	0.1486	-22.7257
44.63334	3.585859	0.1711182	-22.73263
44.65	3.131336	0.1080751	-22.74648
44.66667	3.071212	0.1576055	-22.76727
44.68333	3.202645	0.1125779	-22.78806
44.7	3.310399	0.1621082	-22.80191
44.71667	3.435911	0.1441017	-22.80884
44.73333	3.471855	0.08555687	-22.80191
44.75	3.603425	0.1576055	-22.81577
44.76667	3.161257	0.1035724	-22.85041
44.78333	3.352181	0.1576055	-22.8712
44.8	3.292427	0.1395945	-22.8712
44.81667	3.328294	0.1260862	-22.87813
44.83333	3.406049	0.1035679	-22.88506
44.85	3.352306	0.1350917	-22.87813
44.86666	3.298444	0.1440972	-22.88506
44.88334	3.388095	0.1576055	-22.87813
44.9	3.459989	0.1215834	-22.87813
44.91667	3.400176	0.1215834	-22.89891
44.93333	3.525729	0.1215789	-22.92663
44.95	3.436173	0.1350917	-22.94741
44.96667	3.483828	0.1621082	-22.94048
44.98333	3.131258	0.09005962	-22.92663
45	3.298378	0.1080706	-22.96127
45.01667	3.45972	0.08555687	-22.99591
45.03333	3.543671	0.1305844	-22.98898
45.05	3.47195	0.1440972	-22.95434
45.06667	3.430109	0.166611	-22.94048
45.08333	3.35843	0.1305889	-22.96127
45.1	3.53741	0.1440972	-22.9682
45.11666	3.495981	0.1125734	-22.9682
45.13334	3.418136	0.1576055	-22.9682
45.15	3.525747	0.1170762	-22.96127
45.16667	3.328544	0.1350917	-22.9682
45.18333	3.531549	0.1350917	-22.98206
45.2	3.555621	0.166611	-23.00284
45.21667	3.173171	0.1486	-23.00284
45.23333	3.411892	0.1125779	-23.00284
45.25	3.418154	0.09456238	-23.0167
45.26667	3.537559	0.1305844	-23.00284
45.28333	3.472088	0.1350917	-22.99591
45.3	3.346337	0.1170807	-23.0167
45.31667	3.483715	0.1215789	-23.03748

45.33333	3.328491	0.1260817	-23.04441
45.35	3.244814	0.1260862	-23.05134
45.36666	3.178883	0.1080751	-23.05827
45.38334	3.626995	0.1080706	-23.07213
45.4	3.233014	0.1260817	-23.08598
45.41667	3.573199	0.166611	-23.07906
45.43333	3.173243	0.1440972	-23.07906
45.45	3.567248	0.1215834	-23.07906
45.46667	3.454074	0.1350917	-23.07213
45.48333	3.400331	0.1531027	-23.07906
45.5	3.328288	0.1080751	-23.08598
45.51667	3.746717	0.1486	-23.08598
45.53333	3.418524	0.1350917	-23.09291
45.55	3.406073	0.1170807	-23.10677
45.56667	3.483798	0.1305889	-23.12756
45.58333	3.209055	0.1395945	-23.14834
45.6	3.549294	0.1035679	-23.13448
45.61666	3.424296	0.1486	-23.12063
45.63334	3.382204	0.1576055	-23.1137
45.65	3.364196	0.1350917	-23.10677
45.66667	3.782715	0.1395945	-23.09291
45.68333	3.484138	0.1080706	-23.10677
45.7	3.525687	0.1395945	-23.13448
45.71667	3.358335	0.1666155	-23.15527
45.73333	3.388292	0.09456238	-23.16913
45.75	3.358227	0.1486	-23.17605
45.76667	3.382168	0.1531072	-23.16913
45.78333	3.746741	0.1350872	-23.15527
45.8	3.544023	0.1486	-23.12756
45.81667	3.489916	0.1260862	-23.10677
45.83333	3.382323	0.08105411	-23.1137
45.85	3.400092	0.1621037	-23.12063
45.86666	3.220772	0.1350917	-23.12756
45.88334	3.507596	0.1125779	-23.13448
45.9	3.292565	0.175621	-23.14141
45.91667	3.30449	0.1305889	-23.15527
45.93333	3.435923	0.1215834	-23.15527
45.95	3.591481	0.1395945	-23.15527
45.96667	3.280812	0.09906514	-23.17605
45.98333	3.477573	0.1305889	-23.17605
46	3.53171	0.1260862	-23.1622
46.01667	3.603478	0.1260862	-23.13448
46.03333	3.406306	0.1080706	-23.13448
46.05	3.579406	0.1260862	-23.1622
46.06667	3.537828	0.1215789	-23.17605
46.08333	3.316559	0.1215834	-23.16913
46.1	3.651056	0.1170762	-23.1622

46.11666	3.418363	0.1125734	-23.15527
46.13334	3.507781	0.1080706	-23.1622
46.15	3.430187	0.1350872	-23.17605
46.16667	3.746741	0.09005962	-23.16913
46.18333	3.442315	0.1080706	-23.1622
46.2	3.663142	0.08105411	-23.15527
46.21667	3.448218	0.1530982	-23.16913
46.23333	3.639195	0.1260862	-23.18298
46.25	3.675319	0.1125779	-23.18298
46.26667	3.454223	0.1305889	-23.18298
46.28333	3.382329	0.1125734	-23.18991
46.3	3.519593	0.1125734	-23.18991
46.31667	3.537792	0.1125734	-23.20377
46.33333	3.723015	0.1080706	-23.21763
46.35	3.44219	0.09005962	-23.23148
46.36666	3.722914	0.1170807	-23.25227
46.38334	3.448254	0.1395945	-23.2592
46.4	3.298671	0.1080751	-23.24534
46.41667	3.340231	0.1350917	-23.23841
46.43333	3.549515	0.1305889	-23.23841
46.45	3.579651	0.08555687	-23.24534
46.46667	3.561637	0.1486	-23.25227
46.48333	3.53778	0.09906514	-23.26613
46.5	3.489904	0.1215834	-23.27305
46.51667	3.519814	0.1080706	-23.24534
46.53333	3.519856	0.1080706	-23.23841
46.55	3.430163	0.1305844	-23.24534
46.56667	3.609286	0.1035679	-23.2592
46.58333	3.645421	0.1260862	-23.26613
46.6	3.280794	0.1125734	-23.27305
46.61666	3.382174	0.1440972	-23.27305
46.63334	3.352324	0.1170762	-23.27998
46.65	3.627347	0.1170762	-23.27305
46.66667	3.310644	0.09906514	-23.27305
46.68333	3.53741	0.1350917	-23.27305
46.7	3.478092	0.1035679	-23.2592
46.71667	3.758786	0.1486	-23.25227
46.73333	3.466172	0.1440972	-23.26613
46.75	3.394308	0.1440972	-23.28691
46.76667	3.418076	0.1666155	-23.29384
46.78333	3.274658	0.1486045	-23.29384
46.8	3.698674	0.1486	-23.29384
46.81667	3.717154	0.1486	-23.30077
46.83333	3.496375	0.1305889	-23.29384
46.85	3.453954	0.1035679	-23.27998
46.86666	3.645206	0.1170762	-23.26613
46.88334	3.412412	0.1080751	-23.26613

46.9	3.340279	0.1305889	-23.26613
46.91667	3.507685	0.1080706	-23.26613
46.93333	3.627282	0.1260862	-23.27998
46.95	3.573688	0.1350917	-23.3077
46.96667	3.388435	0.1080751	-23.32848
46.98333	3.543462	0.2161458	-23.33541
47	3.627383	0.1170807	-23.32848
47.01667	3.466214	0.1080706	-23.32848
47.03333	3.633221	0.1531027	-23.33541
47.05	3.675384	0.09906514	-23.32848
47.06667	3.591606	0.1215789	-23.32155
47.08333	3.663363	0.1486	-23.3077
47.1	3.52598	0.1035724	-23.30077
47.11666	3.734947	0.1621082	-23.28691
47.13334	3.854776	0.1395945	-23.27998
47.15	3.705431	0.1486	-23.29384
47.16667	3.764904	0.1531027	-23.32155
47.18333	3.75918	0.1305889	-23.33541
47.2	3.735126	0.1215834	-23.34234
47.21667	3.669511	0.1080706	-23.32848
47.23333	3.723236	0.1486045	-23.32155
47.25	3.507996	0.09456238	-23.32848
47.26667	3.669213	0.1170762	-23.33541
47.28333	3.830883	0.1035679	-23.34927
47.3	3.914434	0.1305844	-23.34927
47.31667	3.5741	0.1395945	-23.34234
47.33333	3.854507	0.09456238	-23.34927
47.35	3.848795	0.1395945	-23.36313
47.36666	3.574076	0.1395945	-23.38391
47.38334	3.938226	0.09906964	-23.38391
47.4	3.735394	0.1350872	-23.37005
47.41667	3.555991	0.1305889	-23.37698
47.43333	3.705174	0.1080706	-23.37005
47.45	3.424194	0.1350917	-23.3562
47.46667	3.764827	0.1666155	-23.3562
47.48333	3.765023	0.1125734	-23.37005
47.5	3.735389	0.1531027	-23.37005
47.51667	3.705216	0.1125779	-23.37005
47.53333	3.639536	0.1035679	-23.3562
47.55	3.842719	0.1215834	-23.3562
47.56667	3.932549	0.1170807	-23.37698
47.58333	3.550052	0.1215789	-23.38391
47.6	3.543689	0.09906964	-23.38391
47.61666	4.039868	0.1305844	-23.39084
47.63334	3.723713	0.09906514	-23.4047
47.65	3.770968	0.1576055	-23.41163
47.66667	3.741226	0.1125779	-23.4047

47.68333	3.950444	0.1125734	-23.41163
47.7	3.663554	0.1170762	-23.41855
47.71667	3.645654	0.1170762	-23.41163
47.73333	3.89639	0.1395945	-23.39084
47.75	3.717398	0.1080706	-23.37698
47.76667	3.777039	0.1621082	-23.37698
47.78333	3.998218	0.07655136	-23.39777
47.8	3.74741	0.1440972	-23.4047
47.81667	3.950449	0.1350917	-23.39777
47.83333	4.267422	0.1170807	-23.4047
47.85	3.927118	0.1170762	-23.42548
47.86666	3.72939	0.13959	-23.44627
47.88334	3.884757	0.1440972	-23.44627
47.9	3.866904	0.09456238	-23.44627
47.91667	3.657621	0.09005962	-23.43241
47.93333	3.866463	0.1531027	-23.41855
47.95	3.813197	0.1395945	-23.42548
47.96667	4.046034	0.1170807	-23.44627
47.98333	3.771398	0.1395945	-23.4532
48	3.938434	0.1215834	-23.43934
48.01667	3.968571	0.1260817	-23.43934
48.03333	3.645899	0.15761	-23.4532
48.05	4.057721	0.1260862	-23.47398
48.06667	3.777438	0.1260862	-23.47398
48.08333	3.926389	0.08105411	-23.4532
48.1	4.207752	0.1215789	-23.44627
48.11666	3.807462	0.1215834	-23.4532
48.13334	3.824848	0.1440972	-23.48091
48.15	3.842916	0.1215834	-23.5017
48.16667	3.884823	0.1621082	-23.48784
48.18333	3.854776	0.1080751	-23.48091
48.2	3.944642	0.166611	-23.48784
48.21667	3.980544	0.1170807	-23.49477
48.23333	3.998456	0.1531027	-23.48784
48.25	3.962686	0.09906514	-23.48784
48.26667	3.902783	0.1260817	-23.48091
48.28333	3.843191	0.1080706	-23.46705
48.3	4.081954	0.1350917	-23.48091
48.31667	4.177729	0.1350917	-23.48784
48.33333	3.789591	0.09456238	-23.48091
48.35	4.099801	0.1440927	-23.47398
48.36666	4.064435	0.1440972	-23.48091
48.38334	3.938888	0.1080751	-23.48091
48.4	3.980479	0.1440972	-23.46705
48.41667	3.926807	0.1350917	-23.46705
48.43333	4.028193	0.09456238	-23.48091
48.45	3.986597	0.1125779	-23.49477

48.46667	3.920761	0.1260817	-23.5017
48.48333	4.01046	0.09005962	-23.49477
48.5	4.14804	0.1801192	-23.48091
48.51667	4.00458	0.1080751	-23.46705
48.53333	3.890798	0.1531027	-23.46013
48.55	4.213619	0.1440972	-23.46705
48.56667	4.17222	0.1080706	-23.48784
48.58333	3.801373	0.1621127	-23.5017
48.6	3.950408	0.09906514	-23.50863
48.61666	4.327193	0.1215834	-23.49477
48.63334	4.136485	0.1486	-23.48784
48.65	3.903111	0.1305889	-23.49477
48.66667	4.070153	0.1170807	-23.5017
48.68333	3.932806	0.0720486	-23.51555
48.7	4.100039	0.1305889	-23.54327
48.71667	3.962668	0.1215834	-23.55713
48.73333	3.93277	0.1395945	-23.55713
48.75	3.992392	0.1531027	-23.5502
48.76667	4.243565	0.1440972	-23.54327
48.78333	4.226088	0.1260862	-23.53634
48.8	4.082431	0.1486	-23.52941
48.81667	3.968762	0.1260862	-23.53634
48.83333	4.076272	0.1260862	-23.54327
48.85	4.231645	0.175621	-23.54327
48.86666	4.136317	0.1305889	-23.54327
48.88334	4.17801	0.1621082	-23.54327
48.9	3.933033	0.1170807	-23.53634
48.91667	4.052176	0.1440972	-23.53634
48.93333	3.831247	0.1305844	-23.54327
48.95	3.86078	0.1486045	-23.54327
48.96667	4.249479	0.1080706	-23.5502
48.98333	3.855247	0.1260862	-23.57791
49	4.046237	0.1035679	-23.58484
49.01667	4.255633	0.1395945	-23.59177
49.03333	4.309621	0.1350917	-23.60563
49.05	3.963008	0.1215834	-23.5987
49.06667	4.171718	0.1666155	-23.57098
49.08333	4.076409	0.1035724	-23.55713
49.1	4.219749	0.1215789	-23.57791
49.11666	3.909181	0.1440972	-23.61255
49.13334	4.159811	0.1215789	-23.62641
49.15	4.21991	0.1170762	-23.61948
49.16667	4.208098	0.1035679	-23.63334
49.18333	4.15417	0.09456238	-23.62641
49.2	4.267744	0.1215789	-23.5987
49.21667	4.064626	0.1305889	-23.5987
49.23333	3.926843	0.09906514	-23.61255

49.25	4.010304	0.1215789	-23.61255
49.26667	4.076218	0.1350917	-23.59177
49.28333	4.034538	0.1621082	-23.59177
49.3	3.932752	0.1531027	-23.60563
49.31667	4.100183	0.1666155	-23.61255
49.33333	4.171766	0.1305889	-23.60563
49.35	3.897125	0.1035679	-23.61255
49.36666	4.392969	0.08555687	-23.61948
49.38334	4.429438	0.1621082	-23.62641
49.4	3.999071	0.1215834	-23.62641
49.41667	3.980628	0.09456238	-23.61948
49.43333	4.087952	0.1035679	-23.61255
49.45	4.160127	0.1035679	-23.60563
49.46667	4.142137	0.1035679	-23.59177
49.48333	4.237858	0.1440972	-23.61255
49.5	4.172202	0.1305889	-23.63334
49.51667	4.148118	0.1215834	-23.64027
49.53333	4.435109	0.1531027	-23.6472
49.55	4.017019	0.1846265	-23.65413
49.56667	4.273432	0.1125779	-23.66105
49.58333	3.998838	0.1125734	-23.65413
49.6	4.064364	0.2071403	-23.62641
49.61666	4.100176	0.1350917	-23.60563
49.63334	4.135911	0.1486	-23.61948
49.65	4.249891	0.1486	-23.63334
49.66667	4.231884	0.1305889	-23.64027
49.68333	4.118477	0.09906514	-23.64027
49.7	4.11218	0.1215789	-23.63334
49.71667	4.142095	0.1260862	-23.62641
49.73333	4.130342	0.1215834	-23.62641
49.75	4.464893	0.09906514	-23.64027
49.76667	4.100726	0.175621	-23.6472
49.78333	4.094291	0.175621	-23.6472
49.8	3.998552	0.1305889	-23.65413
49.81667	4.094291	0.1531027	-23.67491
49.83333	4.327223	0.1486	-23.67491
49.85	4.100534	0.1395945	-23.66798
49.86666	4.088388	0.1440972	-23.6472
49.88334	3.783407	0.1170807	-23.61948
49.9	4.099806	0.1846265	-23.5987
49.91667	4.088299	0.1395945	-23.60563
49.93333	4.118304	0.1711182	-23.62641
49.95	3.992601	0.1125779	-23.62641
49.96667	4.010442	0.166611	-23.60563
49.98333	4.261333	0.1350917	-23.5987
50	4.016972	0.1215789	-23.61255
50.01667	4.249497	0.1395945	-23.64027

50.03333	3.837461	0.1531072	-23.6472
50.05	4.159763	0.1260862	-23.67491
50.06667	4.070487	0.166611	-23.70263
50.08333	4.44698	0.1486	-23.70263
50.1	4.345804	0.1395945	-23.70955
50.11666	4.130492	0.15761	-23.71648
50.13334	4.309484	0.1576055	-23.68877
50.15	4.231931	0.1350917	-23.67491
50.16667	4.076594	0.1305889	-23.68877
50.18333	4.345195	0.1215834	-23.70263
50.2	4.357682	0.1125779	-23.70955
50.21667	4.17222	0.166611	-23.70955
50.23333	4.423261	0.1080751	-23.70955
50.25	4.465484	0.1395945	-23.70955
50.26667	4.339847	0.1440972	-23.70955
50.28333	3.891287	0.1125734	-23.70955
50.3	4.356941	0.1350917	-23.71648
50.31667	4.190335	0.1125779	-23.70955
50.33333	4.160061	0.166611	-23.71648
50.35	4.237834	0.1305889	-23.73727
50.36666	4.17826	0.1350917	-23.7442
50.38334	4.136096	0.1350917	-23.7442
50.4	4.207919	0.1440972	-23.73727
50.41667	4.118286	0.1350917	-23.7442
50.43333	3.998624	0.1260817	-23.7442
50.45	4.423046	0.1080751	-23.75805
50.46667	4.178284	0.1576055	-23.75805
50.48333	4.375343	0.1576055	-23.75805
50.5	4.268096	0.1260862	-23.73727
50.51667	4.028641	0.1350917	-23.70955
50.53333	4.273522	0.1531027	-23.70955
50.55	4.100553	0.166611	-23.72341
50.56667	4.070422	0.1305889	-23.73034
50.58333	4.052408	0.1080751	-23.7442
50.6	4.123992	0.1576055	-23.75805
50.61666	4.315691	0.1305889	-23.75805
50.63334	4.166179	0.1125734	-23.75805
50.65	4.285609	0.1260862	-23.76498
50.66667	4.148177	0.1170807	-23.77884
50.68333	4.321624	0.1486	-23.77884
50.7	4.303795	0.1440972	-23.77884
50.71667	4.220239	0.1486	-23.7927
50.73333	4.536745	0.1080706	-23.79963
50.75	4.363781	0.1621082	-23.80655
50.76667	4.506985	0.1260862	-23.80655
50.78333	4.310224	0.1215834	-23.82734
50.8	4.190138	0.1260817	-23.8412

50.81667	4.16024	0.1666155	-23.81348
50.83333	4.375379	0.1621127	-23.78577
50.85	4.303771	0.1486	-23.7927
50.86666	4.393638	0.1486	-23.78577
50.88334	4.207919	0.1395945	-23.7927
50.9	4.040817	0.09005962	-23.81348
50.91667	4.45286	0.1486	-23.83427
50.93333	4.310045	0.1486045	-23.8412
50.95	3.89722	0.1666155	-23.84813
50.96667	4.195581	0.1666155	-23.8412
50.98333	4.506788	0.1215834	-23.83427
51	4.244424	0.1305889	-23.84813
51.01667	4.279735	0.1125734	-23.85505
51.03333	4.429235	0.1125734	-23.82734
51.05	4.010967	0.1486	-23.80655
51.06667	4.189857	0.1305889	-23.81348
51.08333	4.327652	0.1621127	-23.82041
51.1	4.178117	0.175621	-23.8412
51.11666	4.088418	0.1801238	-23.87584
51.13334	4.405229	0.1125779	-23.88277
51.15	4.004986	0.1260817	-23.86198
51.16667	4.177866	0.1666155	-23.84813
51.18333	4.172052	0.166611	-23.83427
51.2	4.554735	0.1350917	-23.83427
51.21667	4.28614	0.1621127	-23.83427
51.23333	4.046684	0.1080706	-23.85505
51.25	4.291577	0.1125734	-23.86198
51.26667	4.24979	0.1395945	-23.86891
51.28333	4.542953	0.1395945	-23.88277
51.3	4.011164	0.1305889	-23.90355
51.31667	4.117993	0.09456238	-23.90355
51.33333	4.506686	0.1440972	-23.89663
51.35	4.417668	0.09005962	-23.89663
51.36666	4.298012	0.1215789	-23.90355
51.38334	4.417166	0.1440972	-23.91741
51.4	4.303867	0.1350917	-23.9382
51.41667	4.190257	0.1035679	-23.95205
51.43333	4.518922	0.1350917	-23.94513
51.45	4.00507	0.1305889	-23.93127
51.46667	4.141916	0.1260862	-23.91048
51.48333	4.63255	0.1080706	-23.90355
51.5	4.369959	0.1035679	-23.89663
51.51667	4.094488	0.1305889	-23.89663
51.53333	4.423088	0.09456238	-23.90355
51.55	3.867555	0.1305844	-23.91048
51.56667	4.177639	0.1395945	-23.91741
51.58333	4.464994	0.1486045	-23.93127

51.6	4.238246	0.1035679	-23.94513
51.61666	4.220131	0.15761	-23.95205
51.63334	4.190042	0.1260817	-23.96591
51.65	4.465084	0.1305889	-23.97284
51.66667	4.375683	0.1576055	-23.95898
51.68333	4.124457	0.1170807	-23.9382
51.7	4.195856	0.1260862	-23.9382
51.71667	4.495142	0.1711182	-23.97284
51.73333	4.00501	0.1350917	-23.99363
51.75	4.476771	0.1215834	-24.00056
51.76667	4.112681	0.1125734	-24.00056
51.78333	4.022564	0.1395945	-23.99363
51.8	4.411102	0.1395945	-23.97284
51.81667	4.238103	0.1305889	-23.95205
51.83333	4.243863	0.1621082	-23.95898
51.85	4.56681	0.1260862	-23.96591
51.86666	4.346156	0.1170762	-23.99363
51.88334	4.124326	0.09456238	-24.01441
51.9	3.789412	0.1170807	-24.00056
51.91667	4.20161	0.1305844	-23.95898
51.93333	4.393256	0.1531027	-23.94513
51.95	4.339704	0.1666155	-23.97284
51.96667	4.525034	0.1260862	-24.00056
51.98333	4.339871	0.1215834	-24.02134
52	4.124487	0.1395945	-24.02134
52.01667	4.231806	0.175621	-24.00748
52.03333	4.495059	0.1846265	-24.00056
52.05	4.357759	0.1170807	-24.00748
52.06667	4.339769	0.1531027	-24.02827
52.08333	4.232087	0.1215834	-24.02827
52.1	3.992768	0.15761	-24.00748
52.11666	4.183871	0.1395945	-23.99363
52.13334	4.405324	0.1350917	-24.00748
52.15	4.202272	0.1440972	-24.02134
52.16667	4.423088	0.09906514	-24.04213
52.18333	4.405635	0.1440972	-24.05598
52.2	4.321791	0.15761	-24.04906
52.21667	3.956961	0.1531027	-24.02827
52.23333	4.261829	0.0720486	-24.02134
52.25	4.399039	0.1350917	-24.00748
52.26667	4.25016	0.13959	-24.00056
52.28333	4.357592	0.175621	-23.99363
52.3	4.196196	0.1756255	-24.00748
52.31667	4.202004	0.09456238	-24.0352
52.33333	4.02867	0.1350872	-24.04906
52.35	4.596254	0.1395945	-24.05598
52.36666	4.118841	0.1440972	-24.06291

52.38334	4.225652	0.1305889	-24.05598
52.4	4.315763	0.1080706	-24.02134
52.41667	4.351599	0.1305844	-24.01441
52.43333	4.016751	0.175621	-24.04213
52.45	4.363173	0.1440972	-24.04906
52.46667	4.076773	0.175621	-24.0352
52.48333	4.004527	0.1621127	-24.0352
52.5	4.446939	0.1080751	-24.04906
52.51667	4.459437	0.1530982	-24.04906
52.53333	4.35179	0.1125779	-24.06291
52.55	4.309699	0.1621082	-24.09063
52.56667	4.375582	0.1440972	-24.09063
52.58333	4.250052	0.1260862	-24.09063
52.6	4.178159	0.1395945	-24.10448
52.61666	4.046452	0.1395945	-24.11141
52.63334	4.429122	0.1080706	-24.09756
52.65	4.112574	0.1170762	-24.07677
52.66667	4.010555	0.1486	-24.06984
52.68333	4.130092	0.08105411	-24.0837
52.7	4.381294	0.1576055	-24.11141
52.71667	4.255902	0.1531072	-24.12527
52.73333	4.303771	0.175621	-24.11141
52.75	4.333675	0.1350917	-24.11141
52.76667	4.046792	0.1080751	-24.11834
52.78333	4.225539	0.1485955	-24.11141
52.8	4.238145	0.1305889	-24.11834
52.81667	4.160204	0.1215834	-24.13913
52.83333	4.291523	0.1305889	-24.12527
52.85	4.142436	0.09906514	-24.11141
52.86666	3.986638	0.1170762	-24.14605
52.88334	4.345273	0.1080706	-24.15991
52.9	4.148326	0.13959	-24.15298
52.91667	4.118316	0.166611	-24.16684
52.93333	4.124147	0.1260862	-24.15991
52.95	4.184085	0.0720486	-24.15298
52.96667	3.897023	0.1080706	-24.15991
52.98333	4.075967	0.1305889	-24.15298
53	4.18396	0.1260862	-24.15991
53.01667	4.363423	0.1260862	-24.15298
53.03333	3.993162	0.1215834	-24.14605
53.05	4.165738	0.1305889	-24.15991
53.06667	4.566607	0.1440972	-24.19455
53.08333	4.160801	0.1486	-24.20148
53.1	4.201801	0.1305889	-24.20148
53.11666	4.285806	0.08555687	-24.19455
53.13334	4.26781	0.1531027	-24.20148
53.15	4.190156	0.1531072	-24.20148

53.16667	3.962954	0.1395945	-24.20148
53.18333	4.33324	0.1215834	-24.19455
53.2	4.106497	0.1621082	-24.19455
53.21667	4.321558	0.08105411	-24.21534
53.23333	4.309639	0.08555687	-24.23613
53.25	3.783747	0.1440972	-24.24998
53.26667	4.147652	0.1350917	-24.2777
53.28333	4.171933	0.1440972	-24.29155
53.3	4.178212	0.09456238	-24.28463
53.31667	4.225724	0.1035679	-24.28463
53.33333	4.202249	0.1395945	-24.29848
53.35	3.87917	0.1395945	-24.28463
53.36666	4.153794	0.1891293	-24.27077
53.38334	3.914846	0.1350917	-24.27077
53.4	4.100093	0.08555687	-24.2777
53.41667	4.052629	0.09906514	-24.2777
53.43333	4.351486	0.1035679	-24.25691
53.45	4.02263	0.166611	-24.24305
53.46667	4.100242	0.08555687	-24.25691
53.48333	3.998552	0.1215834	-24.27077
53.5	4.249504	0.1305889	-24.26384
53.51667	4.363501	0.1531027	-24.24998
53.53333	4.082706	0.166611	-24.24305
53.55	4.040399	0.1305889	-24.22227
53.56667	4.267661	0.1305889	-24.2292
53.58333	3.986788	0.1125734	-24.23613
53.6	4.106169	0.1395945	-24.24305
53.61666	4.16597	0.1215834	-24.24998
53.63334	3.873094	0.1486	-24.27077
53.65	4.093975	0.1440972	-24.26384
53.66667	3.944976	0.06754585	-24.24305
53.68333	4.159685	0.08555687	-24.22227
53.7	4.166263	0.1621082	-24.20148
53.71667	4.279646	0.1350917	-24.1807
53.73333	4.190197	0.1440972	-24.1807
53.75	3.944815	0.1621082	-24.19455
53.76667	3.813102	0.1666155	-24.20841
53.78333	4.159727	0.1170807	-24.20148
53.8	3.926968	0.1350872	-24.19455
53.81667	4.297224	0.1395945	-24.19455
53.83333	4.220197	0.1080706	-24.1807
53.85	3.717667	0.1350917	-24.17377
53.86666	3.878615	0.1576055	-24.1807
53.88334	4.08806	0.1305889	-24.18763
53.9	4.195916	0.1170807	-24.17377
53.91667	3.950802	0.1035679	-24.15298
53.93333	3.723552	0.1756165	-24.14605

53.95	4.285143	0.1440972	-24.14605
53.96667	3.933074	0.1305889	-24.15298
53.98333	3.968571	0.1305889	-24.16684
54	4.022403	0.1260817	-24.17377
54.01667	4.004592	0.1215834	-24.17377
54.03333	3.885056	0.1486	-24.14605
54.05	4.189631	0.1440972	-24.11834
54.06667	3.825439	0.1125779	-24.11141
54.08333	4.028181	0.1711137	-24.09756
54.1	3.836917	0.1350917	-24.09063
54.11666	3.902896	0.1170762	-24.07677
54.13334	3.944529	0.1531027	-24.05598
54.15	3.968446	0.1531027	-24.06291
54.16667	3.98058	0.1531027	-24.07677
54.18333	4.201807	0.1395945	-24.0837
54.2	4.136096	0.1711182	-24.07677
54.21667	3.938936	0.1711182	-24.06291
54.23333	4.285316	0.1440972	-24.06291
54.25	4.070673	0.1215834	-24.04906
54.26667	4.243773	0.1440972	-24.04213
54.28333	4.375284	0.1260862	-24.04906
54.3	4.05288	0.1711182	-24.0352
54.31667	4.034431	0.1711182	-24.02134
54.33333	4.118172	0.1125779	-24.02827
54.35	3.855158	0.1170762	-24.0352
54.36666	4.123777	0.1395945	-24.01441
54.38334	4.046618	0.1260862	-23.9867
54.4	3.759258	0.08555687	-23.97977
54.41667	3.860846	0.1170762	-23.97977
54.43333	3.968469	0.1621082	-23.9867
54.45	4.082067	0.1981393	-23.9867
54.46667	4.076397	0.1350917	-23.96591
54.48333	3.938643	0.1395945	-23.9382
54.5	3.908835	0.1125779	-23.91048
54.51667	3.950665	0.09456238	-23.90355
54.53333	4.261393	0.1305889	-23.8897
54.55	3.974969	0.1080706	-23.85505
54.56667	4.279425	0.08555687	-23.8412
54.58333	4.016667	0.1170762	-23.86198
54.6	3.992583	0.1531027	-23.87584
54.61666	4.022314	0.1305889	-23.86891
54.63334	4.022499	0.1215789	-23.86891
54.65	3.96866	0.1395945	-23.86891
54.66667	4.124033	0.1395945	-23.86891
54.68333	3.891138	0.1350917	-23.84813
54.7	3.878812	0.08555687	-23.82734
54.71667	3.890833	0.1531027	-23.79963

54.73333	3.920671	0.1035724	-23.77191
54.75	3.884805	0.1215789	-23.76498
54.76667	3.854854	0.1440972	-23.75805
54.78333	3.908614	0.1395945	-23.7442
54.8	4.183912	0.1531027	-23.72341
54.81667	3.914959	0.1305889	-23.70955
54.83333	3.819047	0.1891293	-23.70955
54.85	3.729366	0.1531072	-23.70955
54.86666	4.15959	0.1170807	-23.68877
54.88334	4.010597	0.08105411	-23.66105
54.9	3.944767	0.09456238	-23.63334
54.91667	3.920689	0.09005962	-23.63334
54.93333	3.902759	0.1395945	-23.64027
54.95	3.699582	0.09906514	-23.64027
54.96667	3.914356	0.1215789	-23.61948
54.98333	3.609996	0.08105411	-23.57791
55	3.788898	0.1711137	-23.55713
55.01667	3.729145	0.1801238	-23.57791
55.03333	3.818969	0.1170807	-23.60563
55.05	4.285197	0.1215834	-23.58484
55.06667	3.921125	0.1125734	-23.53634
55.08333	3.926634	0.1305889	-23.48784
55.1	4.315291	0.09005962	-23.47398
55.11666	3.98699	0.1260817	-23.4532
55.13334	3.902837	0.1486	-23.43241
55.15	4.177717	0.1260862	-23.43241
55.16667	3.646125	0.1350917	-23.43934
55.18333	3.872593	0.1125734	-23.41855
55.2	3.717243	0.1440972	-23.37698
55.21667	3.651628	0.1350917	-23.3562
55.23333	3.794814	0.1125734	-23.34234
55.25	3.992291	0.1305889	-23.31463
55.26667	3.711555	0.1305889	-23.28691
55.28333	3.812827	0.1125734	-23.25227
55.3	3.890696	0.1305889	-23.22455
55.31667	3.657627	0.1395945	-23.20377
55.33333	3.824783	0.1080706	-23.15527
55.35	3.950414	0.1531027	-23.1137
55.36666	3.96259	0.1531072	-23.09291
55.38334	3.92673	0.1260817	-23.07213
55.4	4.016339	0.1125734	-23.05134
55.41667	3.699683	0.1486	-23.02363
55.43333	4.010155	0.08555687	-22.98206
55.45	4.046416	0.1260817	-22.94741
55.46667	3.861007	0.1215834	-22.93356
55.48333	4.147759	0.1531027	-22.91277
55.5	3.789406	0.1125734	-22.88506

55.51667	3.998409	0.1170807	-22.84348
55.53333	3.681705	0.1170762	-22.82963
55.55	3.597647	0.1440972	-22.80884
55.56667	3.657382	0.1260862	-22.78113
55.58333	3.860703	0.1350872	-22.73956
55.6	3.836917	0.09906514	-22.69106
55.61666	3.633651	0.1486	-22.64256
55.63334	3.902514	0.1215834	-22.60098
55.65	3.986453	0.1305844	-22.55248
55.66667	3.801332	0.1170807	-22.51784
55.68333	3.896533	0.09906514	-22.50398
55.7	3.789245	0.1305844	-22.49706
55.71667	4.06391	0.1440972	-22.4832
55.73333	3.998719	0.1305889	-22.46934
55.75	3.580009	0.1305889	-22.4347
55.76667	4.057768	0.09005962	-22.41391
55.78333	3.885098	0.1080706	-22.3862
55.8	3.890869	0.1215789	-22.35155
55.81667	3.621647	0.1711182	-22.31691
55.83333	3.938273	0.1711182	-22.2892
55.85	3.771374	0.1305889	-22.28227
55.86666	3.705348	0.1215834	-22.26148
55.88334	3.932436	0.07655136	-22.2407
55.9	3.771249	0.1440927	-22.2407
55.91667	3.753181	0.09005962	-22.24763
55.93333	3.806984	0.1035679	-22.2407
55.95	3.842892	0.1486	-22.23377
55.96667	3.765167	0.1035724	-22.2407
55.98333	3.526033	0.1080706	-22.23377
56	3.663136	0.08105411	-22.21298
56.01667	3.872706	0.1576055	-22.21991
56.03333	4.100027	0.1260862	-22.21991
56.05	3.705664	0.1486	-22.20605
56.06667	3.777009	0.1440972	-22.20605
56.08333	3.538162	0.1305889	-22.21298
56.1	3.830411	0.1170807	-22.22684
56.11666	3.651718	0.1350872	-22.2407
56.13334	3.806811	0.1395945	-22.27534
56.15	3.884721	0.09456238	-22.28227
56.16667	3.950461	0.09906514	-22.28227
56.18333	4.094142	0.1531027	-22.27534
56.2	3.813275	0.1125779	-22.2892
56.21667	3.896689	0.1305889	-22.31691
56.23333	3.932746	0.15761	-22.34463
56.25	3.723367	0.139599	-22.36541
56.26667	3.681407	0.1125779	-22.39313
56.28333	3.681401	0.1260817	-22.42084

56.3	3.938411	0.1125779	-22.44855
56.31667	3.729455	0.1440972	-22.4832
56.33333	3.621606	0.1440972	-22.50398
56.35	3.597593	0.1170762	-22.50398
56.36666	4.003912	0.09456238	-22.51091
56.38334	3.897071	0.08555687	-22.52477
56.4	3.89075	0.1215789	-22.55941
56.41667	3.65173	0.1215834	-22.60098
56.43333	3.717034	0.1440972	-22.61484
56.45	3.735293	0.1260862	-22.6287
56.46667	3.693332	0.1305889	-22.63563
56.48333	3.579705	0.09906514	-22.64948
56.5	3.639428	0.1576055	-22.6772
56.51667	3.478116	0.1440972	-22.69798
56.53333	3.519659	0.1440972	-22.71877
56.55	3.800788	0.1440972	-22.75341
56.56667	3.938357	0.1215834	-22.79498
56.58333	3.574172	0.1305889	-22.85041
56.6	3.824699	0.1440972	-22.87813
56.61666	3.597874	0.1486	-22.88506
56.63334	4.039737	0.1440972	-22.89198
56.65	3.843089	0.1350917	-22.91277
56.66667	3.723451	0.1260862	-22.94048
56.68333	3.992291	0.1125779	-22.94741
56.7	3.663626	0.1305889	-22.95434
56.71667	3.639357	0.1576055	-22.97513
56.73333	3.753008	0.1486	-22.98206
56.75	3.962471	0.1080706	-22.98898
56.76667	3.591941	0.1350917	-23.02363
56.78333	3.950127	0.1215834	-23.04441
56.8	3.669815	0.08105411	-23.05827
56.81667	3.729157	0.166611	-23.0652
56.83333	4.165529	0.1125779	-23.07906
56.85	4.064364	0.1215789	-23.10677
56.86666	3.544286	0.1305889	-23.13448
56.88334	3.794778	0.1350917	-23.16913
56.9	3.920641	0.1621082	-23.17605
56.91667	3.926688	0.139599	-23.18298
56.93333	3.807169	0.1711182	-23.19684
56.95	3.795064	0.166611	-23.21763
56.96667	3.866672	0.1350917	-23.23841
56.98333	4.00433	0.1486	-23.2592
57	3.896916	0.1215834	-23.27998
57.01667	3.860912	0.1215789	-23.31463
57.03333	3.741232	0.1260862	-23.34234
57.05	3.830961	0.1080706	-23.34927
57.06667	3.759246	0.08555687	-23.37698

57.08333	3.484079	0.166611	-23.41163
57.1	3.687155	0.1260862	-23.43241
57.11666	4.015975	0.1170807	-23.4532
57.13334	3.484628	0.1125734	-23.47398
57.15	3.83034	0.1305844	-23.5017
57.16667	3.645737	0.09005962	-23.53634
57.18333	3.496011	0.1756165	-23.58484
57.2	3.854525	0.1621127	-23.60563
57.21667	3.795011	0.1125779	-23.61948
57.23333	3.711227	0.1170762	-23.6472
57.25	3.837031	0.1305889	-23.68184
57.26667	3.842791	0.1305889	-23.71648
57.28333	4.088036	0.1170807	-23.7442
57.3	3.592048	0.1395945	-23.75805
57.31667	4.069867	0.1350917	-23.77884
57.33333	3.884888	0.1486	-23.79963
57.35	3.920743	0.1170807	-23.81348
57.36666	3.723415	0.1260817	-23.81348
57.38334	3.824777	0.1260862	-23.82734
57.4	3.460353	0.09906514	-23.86891
57.41667	3.603574	0.1215789	-23.88277
57.43333	3.639273	0.1440972	-23.88277
57.45	3.705174	0.1395945	-23.90355
57.46667	3.645433	0.1170762	-23.91741
57.48333	3.747165	0.1035679	-23.94513
57.5	3.812881	0.1711137	-23.97977
57.51667	3.992344	0.1260862	-24.01441
57.53333	3.699552	0.1170807	-24.02827
57.55	3.950348	0.1576055	-24.01441
57.56667	3.84898	0.1440972	-24.02134
57.58333	3.711489	0.2161548	-24.02827
57.6	3.753193	0.1260862	-24.02827
57.61666	3.472082	0.1125734	-24.0352
57.63334	3.986065	0.1305889	-24.06291
57.65	3.681652	0.1305889	-24.07677
57.66667	3.645499	0.1350917	-24.07677
57.68333	3.842647	0.1215834	-24.10448
57.7	3.675539	0.1170807	-24.12527
57.71667	3.88465	0.1170762	-24.13913
57.73333	3.615756	0.1486	-24.15298
57.75	3.466119	0.09456238	-24.15991
57.76667	3.507781	0.166611	-24.17377
57.78333	3.836625	0.1576055	-24.1807
57.8	3.801117	0.2206576	-24.17377
57.81667	3.812887	0.08105411	-24.16684
57.83333	3.836923	0.09005962	-24.17377
57.85	3.603848	0.1215834	-24.17377

57.86666	3.758888	0.1531027	-24.17377
57.88334	3.729336	0.1350917	-24.17377
57.9	3.806817	0.09456238	-24.15298
57.91667	3.538066	0.1440927	-24.1322
57.93333	3.759025	0.1801238	-24.13913
57.95	3.920391	0.1125779	-24.15991
57.96667	3.866857	0.1621082	-24.17377
57.98333	3.831038	0.09906514	-24.18763
58	3.657502	0.1350917	-24.16684
58.01667	3.723308	0.211643	-24.13913
58.03333	3.783043	0.1621127	-24.11834
58.05	3.770974	0.2116521	-24.0837
58.06667	3.7114	0.1170807	-24.07677
58.08333	3.806924	0.1035679	-24.09063
58.1	3.460144	0.1215789	-24.06984
58.11666	3.836559	0.1621082	-24.0352
58.13334	3.322999	0.1350917	-24.00056
58.15	3.794509	0.1666155	-23.96591
58.16667	3.609716	0.1170807	-23.95898
58.18333	3.776854	0.1305844	-23.95898
58.2	3.9145	0.1305889	-23.95898
58.21667	3.837138	0.1305889	-23.94513
58.23333	3.771052	0.1576055	-23.92434
58.25	4.106056	0.1305889	-23.90355
58.26667	3.693732	0.1576055	-23.89663
58.28333	3.753056	0.2071448	-23.90355
58.3	3.926318	0.15761	-23.89663
58.31667	3.86099	0.1125734	-23.87584
58.33333	3.944594	0.1305889	-23.83427
58.35	3.836989	0.1350917	-23.80655
58.36666	3.765274	0.15761	-23.78577
58.38334	3.609656	0.1531027	-23.77191
58.4	3.621558	0.1621127	-23.75113
58.41667	3.980168	0.09906514	-23.73727
58.43333	3.854955	0.1125734	-23.70955
58.45	3.902729	0.1531027	-23.67491
58.46667	3.609883	0.1395945	-23.6472
58.48333	3.663351	0.1891293	-23.62641
58.5	3.800812	0.1350917	-23.60563
58.51667	3.66966	0.1080706	-23.58484
58.53333	3.836768	0.1350917	-23.56405
58.55	3.765137	0.1170807	-23.56405
58.56667	3.663488	0.1305844	-23.56405
58.58333	3.663375	0.1305889	-23.54327
58.6	3.567696	0.1576055	-23.50863
58.61666	3.591756	0.175621	-23.49477
58.63334	4.099586	0.1305889	-23.50863

58.65	3.46061	0.1711182	-23.50863
58.66667	3.818611	0.1486	-23.48091
58.68333	3.884709	0.1125779	-23.4532
58.7	3.771273	0.1215789	-23.42548
58.71667	3.639476	0.1395945	-23.4047
58.73333	4.087755	0.1215789	-23.39084
58.75	3.914959	0.1531027	-23.38391
58.76667	3.789227	0.1440972	-23.37698
58.78333	3.687352	0.1260817	-23.37005
58.8	3.795046	0.1350917	-23.3562
58.81667	3.741256	0.1305889	-23.34234
58.83333	3.531978	0.1170762	-23.33541
58.85	3.507859	0.1576055	-23.33541
58.86666	3.64529	0.175621	-23.33541
58.88334	3.639458	0.1711182	-23.32155
58.9	3.513983	0.1440972	-23.30077
58.91667	3.63916	0.1260862	-23.29384
58.93333	3.986238	0.1576055	-23.29384
58.95	3.568089	0.1305889	-23.29384
58.96667	3.711113	0.1981348	-23.3077
58.98333	3.741142	0.1350917	-23.31463
59	3.705216	0.1531072	-23.29384
59.01667	3.795088	0.1350917	-23.26613
59.03333	3.555967	0.1260862	-23.23841
59.05	3.794629	0.1531027	-23.24534
59.06667	3.663542	0.09906514	-23.27305
59.08333	3.854824	0.1305844	-23.29384
59.1	3.842797	0.1486	-23.3077
59.11666	3.729366	0.15761	-23.32848
59.13334	3.394547	0.1621082	-23.36313
59.15	3.69298	0.1486045	-23.39084
59.16667	3.484156	0.1260862	-23.4047
59.18333	3.477842	0.1305889	-23.41855
59.2	3.585512	0.1350872	-23.43934
59.21667	3.830722	0.1035679	-23.44627
59.23333	3.842886	0.1305889	-23.44627
59.25	3.848861	0.1486	-23.4532
59.26667	3.8609	0.1215834	-23.43934
59.28333	3.699373	0.2071403	-23.43241
59.3	3.759108	0.139599	-23.44627
59.31667	3.651599	0.1531027	-23.42548
59.33333	3.561888	0.08555687	-23.39777
59.35	3.926115	0.1170762	-23.41163
59.36666	3.663697	0.1846265	-23.43934
59.38334	3.645409	0.1350917	-23.44627
59.4	3.938428	0.1711182	-23.46013
59.41667	3.657573	0.1891338	-23.48091

59.43333	3.950461	0.1035679	-23.4532
59.45	3.938601	0.1621082	-23.41163
59.46667	3.59203	0.1531072	-23.4047
59.48333	3.579615	0.1621082	-23.43934
59.5	3.794826	0.09456238	-23.47398
59.51667	3.747177	0.1125734	-23.5017
59.53333	3.621653	0.1305889	-23.5017
59.55	3.669427	0.1440972	-23.48784
59.56667	3.872485	0.1305889	-23.47398
59.58333	3.66963	0.1305889	-23.49477
59.6	3.657329	0.1305889	-23.5017
59.61666	3.806918	0.1350917	-23.49477
59.63334	3.84285	0.1170807	-23.47398
59.65	3.896671	0.1531027	-23.46013
59.66667	3.633961	0.1440972	-23.43934
59.68333	3.734881	0.1170762	-23.42548
59.7	3.687435	0.1215834	-23.41855
59.71667	3.472183	0.1080706	-23.41163
59.73333	3.53772	0.1395945	-23.4047
59.75	3.657245	0.1531027	-23.39777
59.76667	3.561816	0.1621082	-23.37698
59.78333	3.81266	0.1440972	-23.37005
59.8	3.508079	0.1576055	-23.36313
59.81667	3.442142	0.1621127	-23.33541
59.83333	3.734881	0.1035679	-23.31463
59.85	3.65746	0.1350917	-23.30077
59.86666	3.669362	0.1440972	-23.27998
59.88334	3.615553	0.1440972	-23.24534
59.9	3.723212	0.1531072	-23.21763
59.91667	3.681341	0.1576055	-23.21763
59.93333	3.579884	0.1486045	-23.2107
59.95	3.6273	0.1440972	-23.18991
59.96667	3.454134	0.1215834	-23.15527
59.98333	3.800526	0.1080706	-23.10677
60	3.633722	0.1125734	-23.07213
60.01667	3.466149	0.175621	-23.05827
60.03333	3.770819	0.1711182	-23.05134
60.05	3.764988	0.08555687	-23.04441
60.06667	3.364614	0.1080706	-23.03748
60.08333	3.370308	0.1260817	-23.00977
60.1	3.615207	0.1486	-22.97513
60.11666	3.633621	0.1531072	-22.93356
60.13334	3.687065	0.139599	-22.88506
60.15	3.43036	0.09456238	-22.82963
60.16667	3.483757	0.1891248	-22.78113
60.18333	3.729127	0.1621127	-22.74648
60.2	3.723212	0.1035724	-22.69798

60.21667	3.681311	0.1125734	-22.64256
60.23333	3.358603	0.1215834	-22.58713
60.25	3.51366	0.1080706	-22.51091
60.26667	3.603389	0.08555687	-22.45548
60.28333	3.627568	0.1260817	-22.42084
60.3	3.507948	0.1576055	-22.39313
60.31667	3.639231	0.1486	-22.35155
60.33333	3.49607	0.1215834	-22.29613
60.35	3.663244	0.1215789	-22.23377
60.36666	3.681281	0.1621082	-22.17141
60.38334	3.472111	0.1846265	-22.13677
60.4	3.525669	0.1305889	-22.10906
60.41667	3.364542	0.1576055	-22.0467
60.43333	3.621074	0.1305889	-21.96356
60.45	3.591791	0.1531027	-21.9012
60.46667	3.334489	0.1440972	-21.8527
60.48333	3.483763	0.1395945	-21.79727
60.5	3.62145	0.1305889	-21.74184
60.51667	3.681299	0.1486	-21.69334
60.53333	3.466095	0.1260862	-21.63791
60.55	3.340464	0.1080706	-21.56863
60.56667	3.519689	0.1215789	-21.50627
60.58333	3.633292	0.1801238	-21.44391
60.6	3.490042	0.1440972	-21.38156
60.61666	3.495831	0.1350917	-21.33998
60.63334	3.621456	0.1215834	-21.30534
60.65	3.890547	0.1621082	-21.25684
60.66667	3.460311	0.1125779	-21.20141
60.68333	3.460102	0.1170762	-21.14598
60.7	3.50174	0.1531027	-21.08363
60.71667	3.525825	0.1305889	-21.0282
60.73333	3.346415	0.09906514	-20.96584
60.75	3.412071	0.1080706	-20.89656
60.76667	3.537631	0.1260862	-20.82034
60.78333	3.382454	0.1170762	-20.75106
60.8	3.274521	0.1080706	-20.70256
60.81667	3.376044	0.1035679	-20.65406
60.83333	3.459911	0.1170807	-20.60556
60.85	3.507829	0.1576055	-20.55013
60.86666	3.334525	0.1395945	-20.50163
60.88334	3.519545	0.1215834	-20.46006
60.9	3.42432	0.1035679	-20.40463
60.91667	3.519629	0.1486	-20.34227
60.93333	3.495891	0.09456238	-20.28684
60.95	3.471891	0.1170762	-20.2522
60.96667	3.334597	0.1486	-20.21063
60.98333	3.25058	0.1395945	-20.16906

61	3.352169	0.1350917	-20.12749
61.01667	3.202967	0.1395945	-20.08591
61.03333	3.411863	0.09005962	-20.0582
61.05	3.149183	0.09456238	-20.03049
61.06667	3.23841	0.1350872	-19.99584
61.08333	3.525634	0.1215834	-19.94734
61.1	3.406163	0.1440972	-19.88498
61.11666	3.286614	0.1440972	-19.82263
61.13334	3.22073	0.1305889	-19.78799
61.15	3.579221	0.1486	-19.7672
61.16667	3.310656	0.1350917	-19.75334
61.18333	3.513577	0.09005962	-19.72563
61.2	3.412304	0.1350917	-19.70484
61.21667	3.388137	0.1305889	-19.67713
61.23333	3.6391	0.1080706	-19.64249
61.25	3.388495	0.1170762	-19.6217
61.26667	3.334358	0.1215834	-19.60091
61.28333	3.513708	0.1215789	-19.58013
61.3	3.531632	0.1576055	-19.55934
61.31667	3.298695	0.1350917	-19.54549
61.33333	3.286405	0.1305889	-19.51777
61.35	3.459828	0.1440972	-19.49699
61.36666	3.334537	0.1486	-19.49006
61.38334	3.178829	0.08555687	-19.49006
61.4	3.340249	0.1350872	-19.49006
61.41667	3.202782	0.1486	-19.49699
61.43333	3.238601	0.1125779	-19.49006
61.45	3.465719	0.1170762	-19.49699
61.46667	3.238798	0.1080706	-19.51084
61.48333	3.131151	0.1170807	-19.51084
61.5	3.077127	0.1305844	-19.51777
61.51667	3.495318	0.1260862	-19.53163
61.53333	3.298748	0.1440972	-19.53163
61.55	3.053431	0.09005962	-19.53163
61.56667	3.411719	0.1531027	-19.53163
61.58333	3.250723	0.1035679	-19.54549
61.6	3.131103	0.1260862	-19.56627
61.61666	2.987476	0.1080706	-19.59399
61.63334	3.238446	0.1756165	-19.6217
61.65	3.561255	0.09906514	-19.64941
61.66667	3.358514	0.1756165	-19.6702
61.68333	3.089322	0.1350917	-19.68406
61.7	3.184673	0.1711137	-19.70484
61.71667	3.268439	0.1440972	-19.74641
61.73333	3.119195	0.1080751	-19.77413
61.75	3.148938	0.1350917	-19.78799
61.76667	3.184619	0.1801238	-19.80877

61.78333	3.358126	0.184631	-19.8642
61.8	3.178895	0.1486	-19.93349
61.81667	3.143017	0.1125779	-19.98891
61.83333	3.411898	0.166611	-20.05127
61.85	3.143094	0.1711182	-20.09977
61.86666	3.567278	0.202642	-20.13441
61.88334	3.101456	0.1531072	-20.16213
61.9	3.447628	0.1666155	-20.21063
61.91667	3.220921	0.2161503	-20.25913
61.93333	3.041309	0.2161548	-20.29377
61.95	3.579168	0.1486045	-20.30763
61.96667	3.364429	0.2071448	-20.32841
61.98333	3.340303	0.2116521	-20.36999
62	2.921951	0.2431713	-20.42541
62.01667	3.339927	0.2341703	-20.47391
62.03333	3.382257	0.1981393	-20.52241
62.05	3.167065	0.1936365	-20.57091
62.06667	3.250544	0.2341658	-20.60556
62.08333	3.029485	0.315238	-20.61249
62.1	3.328019	0.3017297	-20.61941
62.11666	3.119148	0.2792069	-20.64713
62.13334	3.142963	0.3737828	-20.67484
62.15	3.298229	0.2927197	-20.69563
62.16667	3.286572	0.342259	-20.73027
62.18333	3.208769	0.3692845	-20.77184
62.2	2.975628	0.369289	-20.79263
62.21667	3.202472	0.4188284	-20.76491
62.23333	3.280484	0.3783036	-20.7372
62.25	3.035471	0.3963011	-20.75799
62.26667	3.339921	0.360288	-20.77877
62.28333	3.011662	0.4863832	-20.76491
62.3	3.32208	0.4413646	-20.75106
62.31667	3.561434	0.4413556	-20.75799
62.33333	3.382395	0.5764878	-20.76491
62.35	3.15505	0.490922	-20.74413
62.36666	3.190803	0.6530707	-20.72334
62.38334	3.405852	0.6530752	-20.68177
62.4	3.13124	0.7522034	-20.62634
62.41667	3.340201	0.7161724	-20.55706
62.43333	3.417909	0.8287863	-20.50163
62.45	3.400176	0.806313	-20.46006
62.46667	3.316494	0.936938	-20.3977
62.48333	3.304323	1.027101	-20.30763
62.5	3.262625	0.9685113	-20.22449
62.51667	3.160923	1.162224	-20.1552
62.53333	3.537422	1.099271	-20.09284
62.55	3.334501	1.310977	-20.03049

62.56667	3.764671	1.320086	-19.97506
62.58333	3.454241	1.320096	-19.89884
62.6	3.298623	1.446249	-19.79491
62.61666	3.453865	1.518383	-19.69099
62.63334	3.430061	1.721345	-19.60784
62.65	3.32855	1.933236	-19.51777
62.66667	3.860184	1.757529	-19.41384
62.68333	3.645636	2.0415	-19.30992
62.7	3.639464	1.982996	-19.19906
62.71667	3.370714	2.172323	-19.08127
62.73333	3.704715	2.352749	-18.97042
62.75	3.418386	2.528802	-18.85263
62.76667	3.818521	2.605641	-18.73484
62.78333	3.735424	2.677812	-18.61013
62.8	3.968129	2.871673	-18.49234
62.81667	3.556224	3.0074	-18.3607
62.83333	3.687083	3.007373	-18.22906
62.85	4.022033	3.611904	-18.12513
62.86666	4.052337	3.535965	-18.04199
62.88334	4.010484	3.819922	-17.93113
62.9	3.974766	3.919474	-17.79256
62.91667	4.099956	4.127726	-17.66092
62.93333	4.219893	4.6017	-17.55699
62.95	4.148201	4.466788	-17.44613
62.96667	4.201968	5.252564	-17.32834
62.98333	4.423243	5.402623	-17.21056
63	4.256027	5.547013	-17.12049
63.01667	4.63255	5.710535	-17.04427
63.03333	4.513473	6.054167	-16.97499
63.05	4.399744	6.755134	-16.88492
63.06667	4.632831	6.914811	-16.82256
63.08333	4.74659	7.028046	-16.7602
63.1	4.740865	7.589157	-16.68399
63.11666	4.806516	8.128924	-16.62163
63.13334	4.770805	7.863023	-16.55235
63.15	4.938181	8.586008	-16.48999
63.16667	5.141997	9.127154	-16.44842
63.18333	5.309368	10.05277	-16.41377
63.2	4.938903	10.06372	-16.39299
63.21667	5.064075	10.28514	-16.3722
63.23333	5.590551	10.84838	-16.3722
63.25	5.292213	11.51692	-16.3722
63.26667	5.692468	12.33934	-16.36527
63.28333	5.43596	12.40991	-16.3722
63.3	5.68102	12.47468	-16.39992
63.31667	5.729128	13.44106	-16.43456
63.33333	6.004295	13.86736	-16.47613

63.35	6.082576	15.07042	-16.5177
63.36666	5.765711	15.3096	-16.5662
63.38334	6.249737	15.81024	-16.62163
63.4	6.423704	16.17449	-16.69092
63.41667	6.50818	17.97669	-16.75327
63.43333	6.921398	17.88036	-16.80177
63.45	6.927755	18.87455	-16.86413
63.46667	6.694788	18.79497	-16.94727
63.48333	6.36521	19.16855	-17.04427
63.5	6.920802	21.22776	-17.16206
63.51667	7.143515	22.10746	-17.26599
63.53333	7.012046	23.00791	-17.36299
63.55	7.275472	23.09941	-17.47384
63.56667	7.401366	24.45286	-17.57084
63.58333	7.605194	24.55117	-17.67477
63.6	7.133576	25.94543	-17.80642
63.61666	7.891809	26.91861	-17.93806
63.63334	7.534434	28.05078	-18.0697
63.65	7.899043	28.55681	-18.20134
63.66667	8.133174	29.48746	-18.3122
63.68333	8.181855	30.47311	-18.44384
63.7	8.008284	31.02966	-18.59627
63.71667	8.630284	31.94901	-18.74177
63.73333	8.200747	33.13088	-18.86649
63.75	8.559631	34.53365	-18.99813
63.76667	8.596185	35.69979	-19.12977
63.78333	9.002742	36.03189	-19.26834
63.8	8.956418	37.52975	-19.40691
63.81667	8.782512	37.8117	-19.55934
63.83333	9.608353	39.62241	-19.70484
63.85	9.089916	41.46444	-19.82956
63.86666	9.616214	42.11644	-19.95427
63.88334	9.251628	43.62446	-20.08591
63.9	9.615419	44.63443	-20.21063
63.91667	10.12025	45.1576	-20.33534
63.93333	9.911853	46.01907	-20.45313
63.95	10.19912	47.48949	-20.55706
63.96667	10.24172	48.25829	-20.66791
63.98333	10.26041	50.40318	-20.80648
64	10.28984	51.06522	-20.92427
64.01667	10.54739	52.52015	-21.04206
64.03333	10.87794	54.1039	-21.15291
64.05	10.62692	55.21068	-21.26377
64.06667	10.84274	55.44897	-21.36077
64.08334	10.54894	57.95173	-21.45084
64.1	11.09383	57.79234	-21.52706
64.11667	11.31197	59.15672	-21.61713

64.13333	11.32366	60.9109	-21.71413
64.15	11.41378	61.68515	-21.79034
64.16666	11.75601	62.95029	-21.85963
64.18333	11.51161	63.70265	-21.94277
64.2	11.88229	65.77711	-22.01206
64.21667	11.93744	67.31994	-22.08827
64.23333	11.65012	68.00868	-22.16448
64.25	11.93608	69.67682	-22.23377
64.26667	11.67421	70.85492	-22.30998
64.28333	12.50005	71.18552	-22.37234
64.3	12.04239	72.72595	-22.42084
64.31667	12.38742	73.06631	-22.49706
64.33334	12.47235	74.49342	-22.5802
64.35	12.83342	74.6076	-22.6287
64.36667	12.08461	76.83003	-22.66334
64.38333	12.81364	78.45323	-22.69798
64.4	12.42599	79.21816	-22.74648
64.41666	12.42572	80.43467	-22.80191
64.43333	13.30701	81.73939	-22.84348
64.45	12.96198	81.7873	-22.85734
64.46667	13.0685	83.4544	-22.86427
64.48333	13.04644	84.4985	-22.89198
64.5	12.84667	84.88465	-22.94048
64.51667	12.76889	84.8448	-22.98898
64.53333	12.41985	86.61088	-23.00977
64.55	13.09124	88.92867	-23.02363
64.56667	13.19524	88.90449	-23.03748
64.58334	13.07623	88.58421	-23.05827
64.6	13.33254	91.34904	-23.09291
64.61667	13.42489	90.79826	-23.13448
64.63333	12.68665	92.68392	-23.18298
64.65	12.94848	92.5641	-23.20377
64.66666	13.09293	92.49778	-23.21763
64.68333	13.14765	94.71489	-23.23148
64.7	12.89491	93.96712	-23.23841
64.71667	13.26103	95.64475	-23.25227
64.73333	13.05191	95.39674	-23.27305
64.75	12.64948	97.33396	-23.27305
64.76667	12.85171	96.38419	-23.25227
64.78333	13.01398	96.89333	-23.26613
64.8	12.70925	97.74957	-23.30077
64.81667	12.77443	98.60176	-23.32155
64.83334	12.91788	97.81756	-23.32155
64.85	12.89561	99.15447	-23.33541
64.86667	12.58174	99.46985	-23.36313
64.88333	12.4921	99.0293	-23.37005
64.9	12.76769	99.65725	-23.3562

64.91666	11.95821	99.4965	-23.34927
64.93333	12.28011	99.73699	-23.37005
64.95	12.50189	99.73001	-23.38391
64.96667	12.46253	99.57021	-23.36313
64.98333	12.05297	99.41901	-23.3562
65	11.93825	100	-23.37698
65.01667	11.90808	98.87917	-23.39777
65.03333	11.78658	99.17874	-23.39777
65.05	12.26181	99.0958	-23.37698
65.06667	11.89592	99.7465	-23.36313
65.08334	11.84817	98.37711	-23.38391
65.1	11.5596	98.42484	-23.4047
65.11667	12.07459	99.69949	-23.42548
65.13333	11.64396	97.83616	-23.42548
65.15	11.48131	98.04558	-23.42548
65.16666	11.37848	97.89172	-23.43241
65.18333	10.9411	97.24522	-23.42548
65.2	10.92677	97.08527	-23.43241
65.21667	11.56315	97.91193	-23.4532
65.23333	11.32394	96.02141	-23.4532
65.25	10.94755	94.737	-23.4532
65.26667	11.10094	94.81683	-23.46013
65.28333	10.69363	93.81465	-23.46013
65.3	10.74743	92.86489	-23.44627
65.31667	10.3987	93.07327	-23.43241
65.33334	10.65039	92.1087	-23.43241
65.35	10.32688	90.25928	-23.46013
65.36667	9.966252	91.28284	-23.48784
65.38333	9.761971	91.01263	-23.49477
65.4	9.772733	89.51349	-23.48784
65.41666	9.827275	87.9613	-23.46705
65.43333	9.600038	86.62132	-23.4532
65.45	9.311214	87.04031	-23.43934
65.46667	9.71816	85.56767	-23.44627
65.48333	9.425266	84.78046	-23.44627
65.5	9.484727	84.32384	-23.42548
65.51667	9.101728	84.09798	-23.41855
65.53333	8.794945	82.84333	-23.41855
65.55	8.914273	81.34467	-23.41163
65.56667	8.74621	80.01744	-23.41163
65.58334	8.734129	79.94122	-23.39777
65.6	8.488483	78.41235	-23.38391
65.61667	8.350367	76.92563	-23.39084
65.63333	8.313813	76.88834	-23.38391
65.65	7.888818	76.14521	-23.38391
65.66666	8.378814	73.59093	-23.38391
65.68333	8.026703	72.97491	-23.37005

65.7	7.864305	72.5032	-23.34927
65.71667	7.534195	69.78606	-23.34234
65.73333	7.827173	70.05798	-23.32155
65.75	7.091658	69.17999	-23.30077
65.76667	7.245366	68.36683	-23.27305
65.78333	7.257972	66.93158	-23.23841
65.8	7.221825	66.10316	-23.20377
65.81667	7.437507	65.74105	-23.17605
65.83334	7.228199	63.6828	-23.14141
65.85	6.862898	62.02407	-23.1137
65.86667	6.850435	61.20461	-23.08598
65.88333	7.023548	60.93972	-23.05827
65.9	6.712586	58.76921	-23.03748
65.91666	6.772155	58.24981	-23.00284
65.93333	6.27523	57.10139	-22.9682
65.95	6.077383	55.89339	-22.94048
65.96667	6.477363	53.8377	-22.91277
65.98333	6.239041	53.56033	-22.85041
66	5.891503	53.29588	-22.78806
66.01667	5.872934	51.54809	-22.74648
66.03333	5.777153	51.069	-22.69798
66.05	5.711664	49.98406	-22.65641
66.06667	5.579252	48.69708	-22.62177
66.08334	5.615477	47.16587	-22.5802
66.1	5.537333	46.65535	-22.51784
66.11667	5.166803	46.26487	-22.44163
66.13333	5.297645	44.9215	-22.37927
66.15	5.010666	43.47978	-22.33077
66.16666	5.070145	42.48845	-22.29613
66.18333	5.034362	42.51778	-22.23377
66.2	5.165789	40.88684	-22.1437
66.21667	4.801263	39.90382	-22.06748
66.23333	4.962224	39.63079	-22.01206
66.25	4.741116	38.08402	-21.93584
66.26667	4.238443	37.57843	-21.83884
66.28333	4.536918	36.12767	-21.76263
66.3	4.615032	35.82803	-21.71413
66.31667	4.405814	34.87757	-21.65177
66.33334	4.626635	33.79539	-21.57556
66.35	4.274405	33.28423	-21.50627
66.36667	4.369452	32.0501	-21.43006
66.38333	4.381479	31.1	-21.37463
66.4	4.285991	31.53391	-21.3192
66.41666	4.160127	30.59074	-21.24991
66.43333	4.130307	28.69599	-21.16677
66.45	4.112293	28.75869	-21.0767
66.46667	3.932824	27.25428	-21.00741

66.48333	3.968499	27.22102	-20.95198
66.5	3.938894	26.63312	-20.8827
66.51667	4.105948	25.92017	-20.81341
66.53333	3.867119	24.8695	-20.77877
66.55	3.896743	24.33211	-20.75799
66.56667	3.932555	23.63433	-20.70949
66.58334	3.753432	22.86406	-20.6402
66.6	3.651521	22.27273	-20.5917
66.61667	3.567851	21.69526	-20.55706
66.63333	3.740975	21.35996	-20.50856
66.65	3.597802	20.31098	-20.43927
66.66666	3.603401	19.8426	-20.37691
66.68333	3.406318	19.39136	-20.3492
66.7	3.501842	18.63589	-20.32149
66.71667	3.460019	18.18852	-20.26606
66.73333	3.441987	17.45638	-20.21063
66.75	3.304669	16.98749	-20.18291
66.76667	3.21469	16.68	-20.16213
66.78333	3.322271	16.29474	-20.14134
66.8	3.489773	15.84184	-20.11363
66.81667	3.537589	15.20981	-20.08591
66.83334	3.233002	14.97276	-20.06513
66.85	3.310238	14.3748	-20.04434
66.86667	3.406079	14.25522	-20.04434
66.88333	3.388214	13.25683	-20.0582
66.9	3.424069	12.68122	-20.07899
66.91666	3.005651	12.57986	-20.09284
66.93333	3.059132	11.81244	-20.1067
66.95	3.208769	11.91931	-20.1067
66.96667	3.16075	11.62427	-20.09977
66.98333	3.32821	10.86475	-20.11363
67	3.26851	10.7157	-20.14134
67.01667	2.838346	10.58047	-20.18291
67.03333	2.891659	10.07446	-20.21063
67.05	3.184458	9.565898	-20.23141
67.06667	2.993564	9.256091	-20.2522
67.08334	2.927675	9.046654	-20.27991
67.1	3.035233	8.688825	-20.30763
67.11667	2.802158	8.687519	-20.35613
67.13333	2.945408	8.322116	-20.41156
67.15	3.035233	7.95329	-20.46006
67.16666	2.62895	7.952926	-20.48777
67.18333	3.022913	7.505572	-20.52934
67.2	3.035286	7.15093	-20.59863
67.21667	2.849973	7.562996	-20.64713
67.23333	2.969379	7.042685	-20.67484
67.25	2.939559	6.820401	-20.70949

67.26667	2.927609	6.498792	-20.75106
67.28333	2.897766	6.308469	-20.80648
67.3	2.664637	6.005371	-20.87577
67.31667	2.957227	6.094822	-20.94506
67.33334	2.64078	5.485397	-21.02127
67.35	2.736256	5.525066	-21.09748
67.36667	2.634656	5.502251	-21.14598
67.38333	2.927341	5.240253	-21.18063
67.4	2.903603	5.127244	-21.24298
67.41666	2.897753	4.756396	-21.29841
67.43333	2.772135	4.89627	-21.33998
67.45	2.640654	4.539107	-21.40234
67.46667	2.712268	4.548238	-21.4647
67.48333	2.556978	4.358141	-21.52013
67.5	2.742016	4.033087	-21.56863
67.51667	2.760197	4.005611	-21.6102
67.53333	2.568975	3.956171	-21.6587
67.55	2.610643	4.000906	-21.72106
67.56667	2.622575	3.617375	-21.78341
67.58334	2.622587	3.459314	-21.8527
67.6	2.837726	3.41381	-21.9012
67.61667	2.437578	3.206165	-21.9497
67.63333	2.32977	3.178815	-22.00513
67.65	2.759779	3.319094	-22.05363
67.66666	2.760191	3.278245	-22.08827
67.68333	2.592761	2.85423	-22.1437
67.7	2.670427	2.831212	-22.20605
67.71667	2.712202	2.808797	-22.26148
67.73333	2.371832	2.849367	-22.32384
67.75	2.586637	2.845107	-22.37234
67.76667	2.437316	2.488403	-22.41391
67.78333	2.64038	2.646229	-22.45548
67.8	2.258199	2.443177	-22.47627
67.81667	2.867277	2.542351	-22.50398
67.83334	2.903669	2.479389	-22.5317
67.85	2.407717	2.47032	-22.55248
67.86667	2.377521	2.289832	-22.58713
67.88333	2.771729	2.136454	-22.63563
67.9	2.628723	2.050758	-22.6772
67.91666	2.521082	2.037024	-22.71877
67.93333	2.604579	2.14528	-22.74648
67.95	2.700294	1.956015	-22.7742
67.96667	2.449468	1.870247	-22.80191
67.98333	2.706042	1.978439	-22.8227
68	2.550997	1.798072	-22.85041
68.01667	2.562798	1.76653	-22.87813
68.03333	2.574831	1.788963	-22.89198

68.05	2.6166	1.685395	-22.91277
68.06667	2.538928	1.779948	-22.93356
68.08334	2.311882	1.608772	-22.95434
68.1	2.335709	1.477899	-22.98898
68.11667	2.431156	1.486869	-23.0167
68.13333	2.461173	1.604201	-23.03748
68.15	2.56275	1.369702	-23.0652
68.16666	2.425456	1.441796	-23.09984
68.18333	2.73597	1.441832	-23.12063
68.2	2.359829	1.423717	-23.1137
68.21667	2.389482	1.279607	-23.12063
68.23333	2.759726	1.39671	-23.14141
68.25	2.527241	1.405706	-23.14834
68.26667	2.437268	1.293084	-23.14141
68.28333	2.568719	1.315561	-23.1622
68.3	2.592689	1.207419	-23.19684
68.31667	2.550902	1.229937	-23.2107
68.33334	2.443291	1.256958	-23.22455
68.35	2.473146	1.288486	-23.23841
68.36667	2.556763	1.320096	-23.26613
68.38333	2.42548	1.171388	-23.3077
68.4	2.335691	1.184887	-23.32848
68.41666	2.479002	1.184833	-23.32848
68.43333	2.305907	1.085727	-23.33541
68.45	2.640332	1.085691	-23.32155
68.46667	2.329854	1.045153	-23.28691
68.48333	2.819515	1.144254	-23.27998
68.5	2.533109	0.9955729	-23.28691
68.51667	2.395546	1.229847	-23.3077
68.53333	2.550711	1.013638	-23.32848
68.55	2.604663	0.8558794	-23.32155
68.56667	2.258157	1.018087	-23.31463
68.58334	2.269886	0.927964	-23.34927
68.6	2.610381	0.9504688	-23.38391
68.61667	2.610601	1.094665	-23.38391
68.63333	2.20445	0.9009655	-23.37005
68.65	2.323527	0.9820241	-23.36313
68.66666	2.586631	0.797294	-23.37698
68.68333	2.616594	0.9009025	-23.39084
68.7	2.323897	0.8017923	-23.38391
68.71667	2.5447	0.9685023	-23.38391
68.73333	2.515006	0.9324623	-23.39084
68.75	2.592606	0.8874077	-23.39777
68.76667	2.306003	0.8558614	-23.41855
68.78333	2.365446	0.8378459	-23.44627
68.8	2.479038	0.8648579	-23.46013
68.81667	2.443356	0.8198078	-23.44627

68.83334	2.222153	0.8378233	-23.43241
68.85	2.568581	0.8423261	-23.43241
68.86667	2.467196	0.7387312	-23.43934
68.88333	2.562762	0.7206571	-23.43934
68.9	2.425468	0.7882435	-23.43934
68.91666	2.287852	0.6756521	-23.44627
68.93333	2.47899	0.7386997	-23.44627
68.95	2.276004	0.6666105	-23.43241
68.96667	2.562618	0.9369065	-23.4532
68.98333	2.383543	0.7567557	-23.47398
69	2.377527	0.7252049	-23.48784
69.01667	2.365571	0.6170622	-23.5017
69.03333	2.311733	0.806232	-23.50863
69.05	2.664243	0.806295	-23.51555
69.06667	2.461328	0.7026641	-23.5017
69.08334	2.407394	0.6531113	-23.49477
69.1	2.335638	0.7386546	-23.50863
69.11667	2.317761	0.7161904	-23.52941
69.13333	2.467022	0.6080432	-23.54327
69.15	2.287953	0.6981568	-23.53634
69.16666	2.210108	0.6305615	-23.52248
69.18333	2.389249	0.6485725	-23.5017
69.2	2.479103	0.761227	-23.48784
69.21667	2.891343	0.7477007	-23.49477
69.23333	2.258366	0.6981614	-23.53634
69.25	2.341594	0.7161769	-23.56405
69.26667	2.568569	0.5494848	-23.55713
69.28333	2.252141	0.6620672	-23.54327
69.3	2.33565	0.6305795	-23.54327
69.31667	2.347545	0.6305569	-23.55713
69.33334	2.365529	0.5900367	-23.55713
69.35	2.120577	0.657596	-23.52941
69.36667	2.371397	0.6350597	-23.5017
69.38333	2.329681	0.7386681	-23.5017
69.4	2.484899	0.5450046	-23.51555
69.41666	2.520968	0.5945169	-23.51555
69.43333	2.108782	0.5494758	-23.50863
69.45	2.275669	0.5990242	-23.51555
69.46667	2.305764	0.7071218	-23.52248
69.48333	2.293838	0.6891468	-23.51555
69.5	2.377377	0.6080612	-23.51555
69.51667	2.449194	0.6936136	-23.51555
69.53333	2.401455	0.5630021	-23.52941
69.55	2.461102	0.6350642	-23.53634
69.56667	2.377479	0.6215559	-23.52941
69.58334	2.413447	0.5494758	-23.52248
69.6	2.22814	0.4368709	-23.51555

69.61667	2.431168	0.6260227	-23.52248
69.63333	2.413369	0.6350687	-23.53634
69.65	2.293838	0.5855294	-23.54327
69.66666	2.359549	0.5539651	-23.5502
69.68333	2.258008	0.6620762	-23.55713
69.7	2.437071	0.6125684	-23.54327
69.71667	2.4074	0.5584993	-23.52941
69.73333	2.491005	0.666579	-23.52941
69.75	2.473093	0.5990286	-23.52248
69.76667	2.526979	0.5134583	-23.52941
69.78333	2.676282	0.5764878	-23.55713
69.8	2.288096	0.5314693	-23.56405
69.81667	2.467016	0.5404658	-23.5502
69.83334	2.323849	0.5314378	-23.52941
69.85	2.341535	0.5044393	-23.5502
69.86667	2.305788	0.4413511	-23.56405
69.88333	2.443153	0.472902	-23.56405
69.9	2.497004	0.5314288	-23.5502
69.91666	2.347701	0.5359676	-23.52248
69.93333	2.174385	0.5044258	-23.52248
69.95	2.628132	0.4864057	-23.55713
69.96667	2.539	0.5359495	-23.59177
69.98333	2.335841	0.5179476	-23.60563
70	2.395314	0.4773957	-23.60563
70.01667	2.353586	0.5539696	-23.5987
70.03333	2.419314	0.4774047	-23.57791
70.05	2.084812	0.481903	-23.57098
70.06667	2.192148	0.526935	-23.58484
70.08334	2.245897	0.5044393	-23.5987
70.1	2.389315	0.5359586	-23.59177
70.11667	2.198308	0.4593802	-23.60563
70.13333	2.305674	0.4368529	-23.62641
70.15	2.311679	0.5359405	-23.63334
70.16666	2.329705	0.508933	-23.62641
70.18333	2.425199	0.5044212	-23.61948
70.2	2.544706	0.5539741	-23.62641
70.21667	2.204426	0.4774182	-23.6472
70.23333	2.413208	0.4413556	-23.6472
70.25	2.514976	0.5314378	-23.63334
70.26667	2.264072	0.4188509	-23.64027
70.28333	2.234007	0.4323501	-23.66105
70.3	2.269892	0.4728839	-23.68184
70.31667	2.371474	0.4368529	-23.67491
70.33334	2.478954	0.5044168	-23.66105
70.35	2.371641	0.4233536	-23.6472
70.36667	2.461078	0.5449415	-23.64027
70.38333	2.258061	0.5269576	-23.63334

70.4	2.472926	0.4323592	-23.65413
70.41666	2.359722	0.4143346	-23.67491
70.43333	2.443213	0.4053246	-23.66105
70.45	2.048945	0.4008218	-23.64027
70.46667	2.269712	0.4413556	-23.6472
70.48333	2.32367	0.5359405	-23.66105
70.5	2.222052	0.5134403	-23.67491
70.51667	2.496908	0.4413556	-23.66798
70.53333	2.204348	0.3873136	-23.66798
70.55	2.234037	0.3918028	-23.65413
70.56667	2.22201	0.4548729	-23.66105
70.58334	2.353484	0.4683812	-23.68184
70.6	2.467106	0.3287642	-23.68184
70.61667	2.305865	0.4458494	-23.67491
70.63333	2.365392	0.4098319	-23.67491
70.65	2.335703	0.4548774	-23.6957
70.66666	2.317767	0.4233401	-23.70955
70.68333	2.544718	0.4278339	-23.70955
70.7	2.18643	0.4188464	-23.70955
70.71667	2.425158	0.3918073	-23.70955
70.73333	2.234097	0.3828108	-23.70955
70.75	2.329585	0.4863832	-23.71648
70.76667	2.383388	0.4368529	-23.72341
70.78333	2.287887	0.3647908	-23.75113
70.8	2.27586	0.4053201	-23.75805
70.81667	2.126486	0.4323501	-23.75113
70.83334	2.227967	0.4368439	-23.7442
70.85	2.36544	0.4233356	-23.7442
70.86667	2.449247	0.4188464	-23.75113
70.88333	2.389393	0.4863967	-23.7442
70.9	2.264042	0.3783081	-23.7442
70.91666	2.233983	0.369289	-23.7442
70.93333	2.413274	0.3377562	-23.73727
70.95	2.347605	0.4143256	-23.75805
70.96667	2.365565	0.4053246	-23.76498
70.98333	2.210144	0.3782991	-23.75805
71	2.478966	0.4098229	-23.76498
71.01667	2.287887	0.3467708	-23.78577
71.03333	2.138507	0.4008218	-23.79963
71.05	2.359358	0.2927287	-23.7927
71.06667	2.317767	0.4188149	-23.79963
71.08334	2.198236	0.4683902	-23.80655
71.1	2.024915	0.3377697	-23.81348
71.11667	2.2936	0.3782856	-23.81348
71.13333	2.281841	0.342268	-23.7927
71.15	2.28784	0.3873001	-23.7927
71.16666	2.335578	0.4188329	-23.82041

71.18333	2.204217	0.4053246	-23.8412
71.2	2.353449	0.3737963	-23.84813
71.21667	2.281889	0.4503477	-23.87584
71.23333	2.281835	0.3017387	-23.90355
71.25	2.263863	0.369289	-23.90355
71.26667	2.28781	0.3377652	-23.88277
71.28333	2.323617	0.306228	-23.88277
71.3	2.228104	0.3782856	-23.8897
71.31667	2.347504	0.3377652	-23.89663
71.33334	2.389452	0.2792069	-23.8897
71.35	2.353592	0.3332535	-23.87584
71.36667	2.198189	0.3422635	-23.86198
71.38333	2.234019	0.3512735	-23.86198
71.4	2.311643	0.2476831	-23.86891
71.41666	2.377461	0.2521859	-23.88277
71.43333	2.210269	0.3287417	-23.87584
71.45	2.323581	0.2927197	-23.88277
71.46667	2.198236	0.2972224	-23.91741
71.48333	2.275795	0.3242435	-23.95898
71.5	2.22805	0.324239	-23.95205
71.51667	2.23994	0.2837187	-23.93127
71.53333	2.078729	0.2882124	-23.92434
71.55	2.192047	0.2927152	-23.92434
71.56667	2.239946	0.3242435	-23.94513
71.58334	2.048832	0.3017342	-23.97284
71.6	2.09053	0.3197317	-23.97284
71.61667	2.502704	0.342268	-23.97284
71.63333	2.26412	0.3422545	-23.97284
71.65	2.25787	0.2927287	-23.99363
71.66666	2.407293	0.3647683	-24.02827
71.68333	2.491017	0.2702059	-24.05598
71.7	1.929528	0.369289	-24.06291
71.71667	2.192023	0.3647773	-24.06291
71.73333	2.317588	0.3377652	-24.06984
71.75	2.251967	0.4053111	-24.06984
71.76667	2.31769	0.3873091	-24.0837
71.78333	2.096678	0.315247	-24.11834
71.8	2.323533	0.3377562	-24.13913
71.81667	2.472979	0.351269	-24.10448
71.83334	2.323825	0.315247	-24.06984
71.85	2.299801	0.3332535	-24.0837
71.86667	2.114578	0.3467663	-24.09756
71.88333	2.239863	0.3242435	-24.09756
71.9	2.090601	0.2927242	-24.09756
71.91666	2.108526	0.3737783	-24.11834
71.93333	2.054741	0.3017297	-24.14605
71.95	2.371242	0.3377562	-24.15298

71.96667	2.192333	0.3377562	-24.13913
71.98333	2.383281	0.3963056	-24.13913
72	1.923487	0.2206666	-24.13913
72.01667	2.275657	0.3197362	-24.1322
72.03333	2.144356	0.2792114	-24.13913
72.05	2.30568	0.2972179	-24.15991
72.06667	2.180282	0.2927197	-24.17377
72.08334	2.239982	0.2837096	-24.17377
72.1	2.210049	0.2972224	-24.18763
72.11667	2.078676	0.2972224	-24.20148
72.13333	2.192118	0.2611959	-24.20841
72.15	2.269814	0.2972179	-24.21534
72.16666	2.234031	0.2837142	-24.21534
72.18333	2.054795	0.2792069	-24.20148
72.2	2.162185	0.2341703	-24.19455
72.21667	2.257859	0.2837051	-24.19455
72.23333	2.473021	0.2656986	-24.20841
72.25	2.407388	0.2927197	-24.22227
72.26667	2.329764	0.2882124	-24.22227
72.28333	2.144416	0.2972224	-24.2292
72.3	2.311649	0.2656986	-24.24305
72.31667	2.32367	0.2431758	-24.26384
72.33334	2.251937	0.2656941	-24.28463
72.35	2.114602	0.2251603	-24.28463
72.36667	2.502775	0.2746997	-24.26384
72.38333	2.401443	0.2747042	-24.27077
72.4	2.252021	0.2972179	-24.29155
72.41666	2.210144	0.2927242	-24.30541
72.43333	1.917387	0.3017252	-24.31234
72.45	2.162137	0.2747042	-24.31234
72.46667	2.042791	0.2296676	-24.30541
72.48333	2.401187	0.2792024	-24.29848
72.5	2.305746	0.2747087	-24.29848
72.51667	2.13252	0.2702014	-24.31927
72.53333	2.245903	0.3287372	-24.34005
72.55	2.275825	0.2927287	-24.34005
72.56667	2.257906	0.1801283	-24.34005
72.58334	2.013031	0.2566796	-24.34698
72.6	2.096457	0.2882169	-24.35391
72.61667	2.239827	0.2656896	-24.35391
72.63333	2.054836	0.2476831	-24.35391
72.65	2.204074	0.2296631	-24.36084
72.66666	2.251908	0.2206576	-24.36084
72.68333	2.156306	0.2521814	-24.36084
72.7	2.102557	0.2161548	-24.38163
72.71667	2.227949	0.2521814	-24.40241
72.73333	2.114506	0.2476786	-24.40241

72.75	2.036811	0.2566886	-24.40241
72.76667	2.102479	0.2837051	-24.40241
72.78333	2.26976	0.2611914	-24.40934
72.8	2.013037	0.2116521	-24.4232
72.81667	2.156192	0.2566796	-24.43705
72.83334	2.192118	0.2702059	-24.43013
72.85	2.060769	0.2882079	-24.43705
72.86667	2.174188	0.1891338	-24.45091
72.88333	2.222022	0.2566841	-24.45784
72.9	2.198195	0.2566841	-24.46477
72.91666	1.869565	0.2071493	-24.47863
72.93333	2.096355	0.2431713	-24.48555
72.95	2.126462	0.2386731	-24.49248
72.96667	2.287625	0.2251603	-24.49941
72.98333	2.371444	0.1756255	-24.50634
73	2.054902	0.2431758	-24.5202
73.01667	2.281692	0.1981393	-24.50634
73.03333	2.114578	0.2251603	-24.50634
73.05	2.120415	0.2792024	-24.51327
73.06667	2.054759	0.2792069	-24.51327
73.08334	2.215922	0.306228	-24.50634
73.1	2.216125	0.2521859	-24.49941
73.11667	2.192118	0.2611914	-24.50634
73.13333	2.072755	0.2611914	-24.49941
73.15	2.114435	0.2206621	-24.49941
73.16666	2.108526	0.1801238	-24.5202
73.18333	2.13834	0.2656896	-24.53405
73.2	2.066684	0.2116521	-24.5202
73.21667	2.108442	0.2116476	-24.52712
73.23333	2.305579	0.2341613	-24.52712
73.25	2.126539	0.1576145	-24.5202
73.26667	2.180205	0.2116476	-24.50634
73.28333	2.036799	0.2296676	-24.49941
73.3	1.947207	0.1350917	-24.50634
73.31667	1.970927	0.1981348	-24.5202
73.33334	2.060656	0.1981393	-24.5202
73.35	2.042738	0.2206531	-24.51327
73.36667	2.150289	0.2341658	-24.50634
73.38333	2.180193	0.1936365	-24.51327
73.4	2.132431	0.1891338	-24.5202
73.41666	2.41316	0.2116476	-24.52712
73.43333	1.965233	0.2026465	-24.54791
73.45	2.281674	0.2431713	-24.55484
73.46667	2.347581	0.1891383	-24.53405
73.48333	2.090703	0.2521769	-24.52712
73.5	2.377318	0.2026465	-24.54098
73.51667	2.078759	0.1801283	-24.54791

73.53333	2.126336	0.175621	-24.54791
73.55	2.233948	0.1846265	-24.56177
73.56667	2.066762	0.1801283	-24.57562
73.58334	2.26976	0.2116476	-24.58255
73.6	2.335602	0.2071448	-24.5687
73.61667	1.971226	0.1936365	-24.5687
73.63333	2.102437	0.2386686	-24.57562
73.65	2.13837	0.1801283	-24.58255
73.66666	2.048712	0.2341613	-24.59641
73.68333	2.395176	0.2161593	-24.60334
73.7	2.066846	0.184631	-24.58948
73.71667	2.102473	0.193632	-24.59641
73.73333	2.048724	0.1891338	-24.61027
73.75	1.994993	0.193632	-24.60334
73.76667	2.102384	0.2341658	-24.58255
73.78333	2.114536	0.2251603	-24.58255
73.8	2.054693	0.2251603	-24.59641
73.81667	2.036775	0.15761	-24.6172
73.83334	2.090512	0.2296631	-24.62412
73.85	1.905366	0.2837006	-24.62412
73.86667	2.227823	0.2476876	-24.61027
73.88333	2.180234	0.16662	-24.59641
73.9	2.150349	0.2566751	-24.60334
73.91666	2.072647	0.1576145	-24.59641
73.93333	2.102473	0.1531072	-24.58948
73.95	2.180175	0.1801238	-24.60334
73.96667	2.35349	0.193632	-24.62412
73.98333	2.186299	0.202642	-24.6172
74	2.275783	0.2341703	-24.59641
74.01667	2.281829	0.202642	-24.59641
74.03333	2.108585	0.2116476	-24.61027
74.05	2.18615	0.2206576	-24.60334
74.06667	2.108555	0.2161503	-24.60334
74.08334	2.341403	0.2161548	-24.60334
74.1	2.156491	0.1891293	-24.6172
74.11667	2.108484	0.1801283	-24.63798
74.13333	1.989007	0.202642	-24.63105
74.15	2.281585	0.1486	-24.6172
74.16666	2.204193	0.1801238	-24.6172
74.18333	2.168327	0.2296631	-24.63105
74.2	2.174158	0.139599	-24.61027
74.21667	2.156318	0.1846265	-24.59641
74.23333	2.11453	0.2116476	-24.61027
74.25	2.012918	0.15761	-24.64491
74.26667	2.036709	0.202642	-24.65877
74.28333	1.983002	0.2251603	-24.64491
74.3	2.239845	0.2071493	-24.63105

74.31667	2.216095	0.184631	-24.63798
74.33334	2.38334	0.1666155	-24.64491
74.35	2.084782	0.1531027	-24.64491
74.36667	2.048736	0.202642	-24.64491
74.38333	2.096475	0.1395945	-24.65877
74.4	2.156294	0.175621	-24.68648
74.41666	2.114476	0.2386686	-24.68648
74.43333	2.192136	0.1350962	-24.67262
74.45	2.156312	0.166611	-24.67955
74.46667	2.144338	0.193632	-24.69341
74.48333	2.084662	0.1981393	-24.70727
74.5	1.953176	0.1801283	-24.72112
74.51667	2.024736	0.1576055	-24.7142
74.53333	2.341403	0.2296631	-24.70034
74.55	2.114596	0.1981393	-24.72112
74.56667	2.311554	0.1936365	-24.73498
74.58334	2.090709	0.184631	-24.72805
74.6	2.144326	0.1936365	-24.73498
74.61667	2.150307	0.1846265	-24.74191
74.63333	2.228033	0.1891338	-24.73498
74.65	2.162292	0.2386641	-24.73498
74.66666	2.413178	0.2161593	-24.74191
74.68333	2.108669	0.1531072	-24.72805
74.7	2.036799	0.1981348	-24.7142
74.71667	2.012876	0.1350917	-24.72112
74.73333	2.000849	0.1801238	-24.73498
74.75	2.090506	0.1981348	-24.74191
74.76667	2.168207	0.1441017	-24.74884
74.78333	1.989043	0.15761	-24.76962
74.8	2.209959	0.2161503	-24.79734
74.81667	2.024921	0.15761	-24.80427
74.83334	2.108454	0.2071448	-24.76962
74.85	2.251866	0.1621127	-24.74884
74.86667	2.132466	0.2161503	-24.75577
74.88333	2.21009	0.1801283	-24.74884
74.9	2.150325	0.2251558	-24.72112
74.91666	2.263803	0.2071493	-24.72805
74.93333	2.096636	0.1891338	-24.75577
74.95	2.233965	0.1621127	-24.77655
74.96667	2.054783	0.2341613	-24.78348
74.98333	2.114411	0.2341703	-24.78348
75	2.269742	0.1486045	-24.79041
75.01667	1.989114	0.2026375	-24.77655
75.03333	2.138292	0.1621127	-24.75577
75.05	2.198057	0.1711182	-24.73498
75.06667	2.078688	0.1350917	-24.72805
75.08334	2.04876	0.1801192	-24.74884

75.1	1.977057	0.2161503	-24.77655
75.11667	2.102372	0.139599	-24.77655
75.13333	2.006967	0.1891293	-24.76962
75.15	2.21591	0.1531072	-24.78348
75.16666	2.204175	0.1486045	-24.79041
75.18333	1.995023	0.175621	-24.79041
75.2	1.971029	0.1125779	-24.79041
75.21667	1.959067	0.1395945	-24.79041
75.23333	1.988911	0.1711182	-24.79041
75.25	2.209959	0.1486	-24.78348
75.26667	2.108567	0.1981348	-24.76962
75.28333	2.26375	0.1756255	-24.77655
75.3	1.989102	0.1711137	-24.79734
75.31667	2.084543	0.1531072	-24.8112
75.33334	2.054687	0.1846265	-24.80427
75.35	2.365303	0.1170807	-24.79734
75.36667	2.19829	0.1891293	-24.80427
75.38333	1.917346	0.1440972	-24.79041
75.4	2.08446	0.2296586	-24.77655
75.41666	2.144344	0.1531072	-24.78348
75.43333	2.287738	0.175621	-24.79734
75.45	2.162322	0.1305889	-24.78348
75.46667	1.839716	0.1846265	-24.79041
75.48333	2.084412	0.211643	-24.80427
75.5	2.192076	0.184631	-24.79734
75.51667	2.156336	0.139599	-24.80427
75.53333	2.138405	0.1170762	-24.79734
75.55	2.096552	0.1531027	-24.79041
75.56667	2.054693	0.1395945	-24.78348
75.58334	2.048676	0.1440972	-24.76962
75.6	1.93521	0.175621	-24.74191
75.61667	1.994916	0.2296586	-24.75577
75.63333	2.186066	0.2296631	-24.78348
75.65	2.084656	0.139599	-24.79041
75.66666	2.084579	0.1260862	-24.80427
75.68333	2.024837	0.1711182	-24.82505
75.7	2.102497	0.1305889	-24.83198
75.71667	1.971047	0.1305889	-24.81812
75.73333	2.16813	0.202642	-24.79734
75.75	2.204193	0.1981348	-24.80427
75.76667	2.054717	0.1981393	-24.80427
75.78333	2.18615	0.175621	-24.80427
75.8	2.108526	0.1936365	-24.81812
75.81667	2.13834	0.1891293	-24.81812
75.83334	2.120421	0.1801283	-24.79734
75.85	1.863585	0.1215834	-24.78348
75.86667	2.126223	0.1756165	-24.77655

75.88333	2.018958	0.1981393	-24.76962
75.9	2.05461	0.175621	-24.77655
75.91666	1.977069	0.1621127	-24.78348
75.93333	2.215892	0.1621127	-24.79041
75.95	2.013025	0.1486	-24.79734
75.96667	2.180037	0.1621127	-24.80427
75.98333	2.066726	0.1801238	-24.78348
76	2.036739	0.1756255	-24.7627
76.01667	1.994993	0.1486	-24.77655
76.03333	2.269683	0.1486045	-24.79041
76.05	2.359381	0.1846265	-24.78348
76.06667	2.060901	0.1395945	-24.7627
76.08334	1.947237	0.15761	-24.7627
76.1	2.227842	0.1891293	-24.78348
76.11667	1.809818	0.1666155	-24.79041
76.13333	2.078473	0.1486045	-24.77655
76.15	2.084597	0.09456238	-24.78348
76.16666	2.156246	0.166611	-24.80427
76.18333	2.138345	0.15761	-24.82505
76.2	2.180211	0.1801238	-24.82505
76.21667	2.072665	0.1260862	-24.81812
76.23333	2.108436	0.166611	-24.80427
76.25	2.120415	0.1531072	-24.79041
76.26667	1.989072	0.1395945	-24.78348
76.28333	2.096403	0.1395945	-24.77655
76.3	2.108514	0.1576055	-24.76962
76.31667	2.078574	0.1170807	-24.76962
76.33334	2.227949	0.1621127	-24.78348
76.35	1.959228	0.1395945	-24.79734
76.36667	2.120314	0.1170807	-24.79041
76.38333	2.030836	0.1846265	-24.7627
76.4	2.174117	0.1846265	-24.74884
76.41666	1.929361	0.139599	-24.75577
76.43333	1.970993	0.1711182	-24.75577
76.45	2.144183	0.1801238	-24.74191
76.46667	2.120475	0.1666155	-24.74884
76.48333	2.084579	0.211643	-24.74884
76.5	2.168219	0.202642	-24.74191
76.51667	2.007003	0.1891338	-24.75577
76.53333	2.060632	0.1170807	-24.79041
76.55	2.090488	0.1440972	-24.80427
76.56667	2.090559	0.1711182	-24.81812
76.58334	2.060722	0.1666155	-24.81812
76.6	2.096463	0.1711182	-24.78348
76.61667	1.977099	0.1350917	-24.74884
76.63333	2.012786	0.1846265	-24.74884
76.65	2.03083	0.1440972	-24.77655

76.66666	1.988971	0.1531027	-24.80427
76.68333	2.096445	0.1621127	-24.81812
76.7	2.180163	0.1305889	-24.81812
76.71667	2.030866	0.1215834	-24.79041
76.73333	2.132329	0.1125734	-24.76962
76.75	2.132377	0.166611	-24.7627
76.76667	2.048772	0.1621082	-24.77655
76.78333	2.162221	0.1801238	-24.8112
76.8	1.905366	0.1350962	-24.80427
76.81667	1.803736	0.1981348	-24.77655
76.83334	2.162054	0.1891338	-24.7627
76.85	1.971142	0.2116476	-24.77655
76.86667	2.221849	0.16662	-24.79041
76.88333	2.084704	0.09005962	-24.78348
76.9	1.935252	0.1350872	-24.79734
76.91666	1.929152	0.1531027	-24.79041
76.93333	2.102455	0.1215834	-24.7627
76.95	1.965102	0.1395945	-24.74884
76.96667	2.018737	0.1440972	-24.75577
76.98333	2.072599	0.1440972	-24.75577
77	2.060698	0.175621	-24.74191
77.01667	2.084573	0.1260862	-24.74884
77.03333	1.899415	0.1395945	-24.74884
77.05	2.102342	0.1531072	-24.74191
77.06667	1.899391	0.166611	-24.75577
77.08334	2.012798	0.1350917	-24.79041
77.1	2.030776	0.1666155	-24.80427
77.11667	2.221915	0.1486	-24.81812
77.13333	1.977159	0.1395945	-24.81812
77.15	1.851534	0.175621	-24.78348
77.16666	2.191957	0.1350917	-24.74884
77.18333	2.12642	0.1846265	-24.74884
77.2	2.06669	0.1305889	-24.77655
77.21667	1.905354	0.1215834	-24.80427
77.23333	2.102401	0.1801192	-24.81812
77.25	1.988935	0.1125779	-24.81812
77.26667	1.994975	0.1531027	-24.79041
77.28333	1.773892	0.15761	-24.76962
77.3	2.126217	0.1440972	-24.7627
77.31667	2.000968	0.1305889	-24.77655
77.33334	2.108394	0.1350917	-24.8112
77.35	2.180103	0.1621082	-24.80427
77.36667	1.923416	0.1395945	-24.77655
77.38333	1.994856	0.1576055	-24.7627
77.4	2.048671	0.1305889	-24.77655
77.41666	2.090554	0.1080706	-24.79041
77.43333	1.893399	0.1485955	-24.79734

77.45	1.833639	0.1260862	-24.79041
77.46667	1.97088	0.1350872	-24.7627
77.48333	2.030782	0.1486	-24.74884
77.5	2.018857	0.1486	-24.75577
77.51667	1.905259	0.1350917	-24.75577
77.53333	1.911299	0.1440972	-24.74191
77.55	1.875379	0.175621	-24.74884
77.56667	2.126253	0.1531027	-24.74884
77.58334	1.851629	0.1486	-24.74191
77.6	1.988858	0.1666155	-24.75577
77.61667	2.006877	0.1621127	-24.79041
77.63333	1.959055	0.1350917	-24.80427
77.65	2.251812	0.1576055	-24.81812
77.66666	2.078694	0.2071448	-24.81812
77.68333	2.144302	0.1621127	-24.78348
77.7	2.096528	0.1395945	-24.74884
77.71667	2.132407	0.1440972	-24.74884
77.73333	2.066655	0.1801283	-24.77655
77.75	1.941215	0.1711182	-24.80427
77.76667	1.875391	0.1666155	-24.81812
77.78333	2.018743	0.1170807	-24.81812
77.8	2.012918	0.1080706	-24.79041
77.81667	1.994844	0.1756165	-24.76962
77.83334	2.221897	0.1215834	-24.7627
77.85	2.174308	0.166611	-24.77655
77.86667	1.953152	0.1350917	-24.8112
77.88333	2.054681	0.09906514	-24.80427
77.9	2.132305	0.1305889	-24.77655
77.91666	1.983098	0.1486	-24.7627
77.93333	1.863507	0.1531072	-24.77655
77.95	1.988852	0.1215834	-24.79041
77.96667	1.797809	0.1621082	-24.79734
77.98333	2.030591	0.175621	-24.79041
78	2.042744	0.1080706	-24.7627
78.01667	2.090554	0.1486	-24.74884
78.03333	2.066667	0.1305889	-24.75577
78.05	2.06065	0.1621127	-24.75577
78.06667	2.054741	0.1125779	-24.74191
78.08334	2.221891	0.09906514	-24.74884
78.1	2.072737	0.13959	-24.74884
78.11667	2.066648	0.1440972	-24.74191
78.13333	2.054663	0.1395945	-24.75577
78.15	1.953212	0.15761	-24.79041
78.16666	2.078515	0.1260862	-24.80427
78.18333	2.13828	0.1531027	-24.81812
78.2	2.108526	0.1486	-24.81812
78.21667	2.144279	0.15761	-24.78348

78.23333	2.204116	0.1170762	-24.74884
78.25	2.198141	0.1486	-24.74884
78.26667	2.216047	0.1531072	-24.77655
78.28333	2.048772	0.1125779	-24.80427
78.3	1.87551	0.1621082	-24.81812
78.31667	2.042636	0.1441017	-24.81812
78.33334	2.162179	0.1395945	-24.79041
78.35	2.078676	0.1305889	-24.76962
78.36667	1.923284	0.1711137	-24.7627
78.38333	2.072528	0.1531072	-24.77655
78.4	1.845672	0.1215834	-24.8112
78.41666	1.988804	0.166611	-24.80427
78.43333	1.839626	0.1666155	-24.77655
78.45	2.132204	0.1531072	-24.7627
78.46667	1.887543	0.1305889	-24.77655
78.48333	1.982835	0.1035679	-24.79041
78.5	1.827641	0.1846265	-24.79734
78.51667	2.072486	0.1260862	-24.79041
78.53333	2.060674	0.1305889	-24.7627
78.55	2.048736	0.1395945	-24.74884
78.56667	1.845643	0.1756165	-24.75577
78.58334	1.994844	0.1080751	-24.75577
78.6	1.857526	0.1080706	-24.74191
78.61667	2.000825	0.1350872	-24.74884
78.63333	1.99491	0.1260862	-24.74884
78.65	2.036697	0.1350917	-24.74191
78.66666	2.120379	0.1395945	-24.75577
78.68333	2.198141	0.1576055	-24.79041
78.7	1.923326	0.1486	-24.80427
78.71667	2.03074	0.1531027	-24.81812
78.73333	2.018767	0.1486045	-24.81812
78.75	2.060668	0.1891293	-24.78348
78.76667	2.000926	0.1621127	-24.74884
78.78333	2.120373	0.1531027	-24.74884
78.8	2.078676	0.05854033	-24.77655
78.81667	1.971065	0.1215789	-24.80427
78.83334	1.905259	0.1440972	-24.81812
78.85	2.120314	0.1305889	-24.81812
78.86667	2.120469	0.1486	-24.79041
78.88333	2.012894	0.1621082	-24.76962
78.9	2.012876	0.1260862	-24.7627
78.91666	1.97698	0.1395945	-24.77655
78.93333	2.156163	0.1666155	-24.8112
78.95	1.995035	0.1531027	-24.80427
78.96667	1.91725	0.1350917	-24.77655
78.98333	2.042654	0.1486045	-24.7627
79	1.977075	0.1305889	-24.77655

79.01667	1.851522	0.1621082	-24.79041
79.03333	1.929164	0.1080751	-24.79734
79.05	2.144159	0.1531027	-24.79041
79.06667	1.953242	0.1666155	-24.7627
79.08334	2.036679	0.139599	-24.74884
79.1	2.006883	0.1576055	-24.75577
79.11667	1.988935	0.1305889	-24.75577
79.13333	1.929229	0.1260862	-24.74191
79.15	2.078568	0.1035724	-24.74884
79.16666	1.869476	0.1621082	-24.74884
79.18333	1.785805	0.1531072	-24.74191
79.2	1.935043	0.1215834	-24.75577
79.21667	1.875468	0.1035679	-24.79041
79.23333	2.07245	0.1035679	-24.80427
79.25	2.048778	0.09906514	-24.81812
79.26667	2.048748	0.1350917	-24.81812
79.28333	2.072587	0.1125779	-24.78348
79.3	1.881419	0.1260817	-24.74884
79.31667	2.096391	0.1440972	-24.74884
79.33334	2.024831	0.1531027	-24.77655
79.35	2.06065	0.15761	-24.80427
79.36667	1.935258	0.1260862	-24.81812
79.38333	1.732092	0.1621127	-24.81812
79.4	2.11416	0.1125779	-24.79041
79.41666	1.899427	0.1125734	-24.76962
79.43333	2.054657	0.1350917	-24.7627
79.45	1.953122	0.1350917	-24.77655
79.46667	2.030723	0.1170807	-24.8112
79.48333	1.977045	0.1080706	-24.80427
79.5	1.923219	0.09456238	-24.77655
79.51667	1.785823	0.1260817	-24.7627
79.53333	1.929104	0.09906514	-24.77655
79.55	1.953075	0.1305889	-24.79041
79.56667	2.000813	0.09906514	-24.79734
79.58334	1.857544	0.1711137	-24.79041
79.6	2.090446	0.175621	-24.7627
79.61667	2.114405	0.1441017	-24.74884
79.63333	2.006967	0.1395945	-24.75577
79.65	1.923237	0.1531072	-24.75577
79.66666	1.976974	0.1981348	-24.74191
79.68333	1.941101	0.1080751	-24.74884
79.7	2.233852	0.1395945	-24.74884
79.71667	1.953242	0.1486	-24.74191
79.73333	1.95311	0.1440972	-24.75577
79.75	1.905295	0.0720486	-24.79041
79.76667	2.156222	0.1170762	-24.80427
79.78333	1.893405	0.1350917	-24.81812

79.8	2.012769	0.1395945	-24.81812
79.81667	2.120386	0.1486	-24.78348
79.83334	1.965155	0.1215834	-24.74884
79.85	2.144183	0.1531027	-24.74884
79.86667	1.881491	0.1035679	-24.77655
79.88333	1.893345	0.1756165	-24.80427
79.9	2.078497	0.1215834	-24.81812
79.91666	2.048676	0.1215834	-24.81812
79.93333	2.006937	0.1170762	-24.79041
79.95	2.048671	0.1395945	-24.76962
79.96667	2.108454	0.1170762	-24.7627
79.98333	2.006913	0.09456238	-24.77655
