# Supplementary material for: Radioactive Labeling of Milk-Derived Exosomes with 99mTc and In Vivo Tracking by SPECT Imaging
Source: Nanomaterials (Basel). 2020 May 30;10(6):1062. doi: 10.3390/nano10061062 (PMC7352469; doi:10.3390/nano10061062)
Supplement: Supplementary file 1 [file nanomaterials-10-01062-s001.zip › Supplementary files_Figure1_2D_3_4_5 available data.pdf]

For ensuring and maintaining the transparency and reproducibility of the results recorded, authors have provided the following research data, openly available as supplementary information:

Table S1. Experimental data from Figure 1B. Radioefficiency of exosomes labeled with 99mTc (VII) vs. 99mTc (IV):

^{99m}**Tc (VII) (% Radioefficiency)**	^{99mTc} **(IV) (% Radioefficiency)**
0.380572	99.53368

Table S2. Experimental data from Figure 1C. Effect of the exosome protein concentration in the radiochemical yield:

**10 ug** (% radiochemical yield)	**30 ug** (% radiochemical yield)	**75 ug** (% radiochemical yield)
0.042337	25.8549	35.8119
0.0965251	20.0012	27.73979
0.0681485	22.69181	46.4049

Table S3. Experimental data from Figure 1D. Determination of optimum concentration of SnCl2 as a reducing agent:

**[$SnCl_2$] = 0.0004 M** (% radiochemical yield)	**[$SnCl_2$] = 0.002 M** (% radiochemical yield)
8.947528	25.8549

**[$SnCl_2$] = 0.004 M** (% radiochemical yield)	**[$SnCl_2$] = 0.006 M** (% radiochemical yield)
22.69181	0.4212573

**[$SnCl_2$] = 0.008 M** (% radiochemical yield)	**[$SnCl_2$] = 0.01 M** (% radiochemical yield)
0.4316547	0.09624639

Table S4. Experimental data from Figure 2D. Longitudinal in vitro stability study of [^{99}mTc]-Exo:

Time (h)	Measure 1 (% stability)	Measure 2 (% stability)	Measure 3 (% stability)
0	99.99348	99.98031	99.98821
1	99.9828	99.99847	99.98371
2	99.98698	99.98277	99.97607
3	99.98872	99.99452	99.99451
4	99.98582	99.98961	99.9865
5	99.9835	99.98859	99.98277
12	99.98089	99.98689	99.98226
21	99.31057	99.81034	99.22935
24	98.88326	99.85741	99.11706
44	99.17173	99.87185	98.51602
48	98.5209	99.46484	97.79107

Table S5. Experimental data from Figure 3C. Ex vivo biodistribution study 24 h after injection:

Organs	Mouse 1 (%ID/g)	Mouse 2 (%ID/g)	Mouse 3 (%ID/g)
Brain	0.02507709	0.02317714	0.03338972
Tyroid	1.032505	1.57446	1.186318
Lungs	0.2957729	0.4062359	0.1747051
Heart	0.1318073	0.1398794	0.07554109
Stomach	2.242374	1.415491	3.285615
Liver	31.87944	32.67875	45.3077
Spleen	18.12445	40.43972	34.7402
Kidneys	1.6454	1.533811	0.990088
Intestines	0.2536879	0.0361366	0.08906547
Feces	4.602346	0.4823191	0.4324131
Muscle	0.06785137	0.06988291	0.04076531
Skin	0.1717769	0.206202	0.180345
Blood	0.2443977	0.1803119	0.2041214

Table S6. Experimental data from Figure 3D. In vivo blood half-life:

Time (min)	%ID/g (normalized)
5	100
15	24.9982
30	20.16352
45	14.12024
60	12.81787
75	14.15323
90	9.541121
105	15.31007
120	9.729397
150	13.30491
180	6.308547
210	7.293179
240	7.951927
270	5.657072
300	10.70875
360	12.5868
1355	9.090429
1450	6.554768

Table S7. Experimental data from Figure 4C. Ex vivo biodistribution study 24 h after injection:

Organs	Mouse 1 (%ID/g)	Mouse 2 (%ID/g)	Mouse 3 (%ID/g)
Brain	0.08444156	0.05708802	0.0175102
Tyroid	14.4406	22.85811	28.8508
Lungs	7.037856	0.9599242	0.3848855
Heart	0.3154105	0.2748781	0.1789648
Stomach	16.44037	28.20265	9.849386
Liver	11.56481	15.89863	5.893948
Spleen	11.40166	9.057914	5.376181
Kidneys	7.592789	5.635682	5.653645
Intestines	1.038048	1.241049	1.518446
Feces	1.453404	1.214827	0.6756303
Muscle	0.1481501	0.1315616	0.1982758
Skin	0.9428526	0.1988679	0.4412295
Blood	3.007046	0.2301117	0.1922312
Bone	0.501948	0.4495765	0.249477

Table S8. Experimental data from Figure 4D. In vivo blood half-life:

Time (min)	%ID/g (normalized)
15	100
30	36.17852
45	35.49108
60	29.80623
120	33.84724
150	28.0876
270	24.29885
1440	0.3685673

Table S9. Experimental data from Figure 5C. Ex vivo biodistribution study 24 h after injection:

Organs	Mouse 1 (%ID/g)	Mouse 2 (%ID/g)	Mouse 3 (%ID/g)
Brain	0.002349962	0.005423833	0.006460233
Tyroid	0.056358	0.04513213	0.06977542
Lungs	0.03088487	0.007491822	0.0067587
Heart	0.01093877	0.0253785	0.06421413
Stomach	0.4226386	0.2638863	0.07102636
Liver	0.0563076	0.01067482	0.009019142
Spleen	0.01566947	0.006133917	0.004281671
Kidneys	0.0204488	0.04698562	0.05342975
Intestines	0.03634911	0.02953024	0.01783494
Feces	0.7556344	0.1409586	0.03605567
Muscle	0.005287857	0.006198084	0.002054
Skin	0.0082722	0.006784981	0.007175661
Blood	0.02904076	0.005553125	0.004469065
Head	0.4154179	0.1999761	0.3107402

Table S10. Experimental data from Figure 5D. In vivo blood half-life:

Time (min)	%ID/g (normalized)
1	100
9	9.926032
21	8.092739
29	3.884516
93	7.649045
107	4.166147
121	4.899386
130	3.499564
147	4.899386
